# Transcriptome Profiling of Human Monocyte-Derived Macrophages Upon CCL2 Neutralization Reveals an Association Between Activation of Innate Immune Pathways and Restriction of HIV-1 Gene Expression

**DOI:** 10.3389/fimmu.2020.02129

**Published:** 2020-09-18

**Authors:** Daniela Angela Covino, Karolina Elżbieta Kaczor-Urbanowicz, Jing Lu, Maria Vincenza Chiantore, Gianna Fiorucci, Maria Fenicia Vescio, Laura Catapano, Cristina Purificato, Clementina Maria Galluzzo, Roberta Amici, Mauro Andreotti, Maria Cristina Gauzzi, Matteo Pellegrini, Laura Fantuzzi

**Affiliations:** ^1^National Center for Global Health, Istituto Superiore di Sanità, Rome, Italy; ^2^UCLA Section of Oral Biology, Division of Oral Biology & Medicine, Center for Oral and Head/Neck Oncology Research, Center for the Health Sciences, UCLA School of Dentistry, University of California at Los Angeles, Los Angeles, CA, United States; ^3^UCLA Institute for Quantitative and Computational Biosciences, University of California at Los Angeles, Los Angeles, CA, United States; ^4^Department of Infectious Diseases, Istituto Superiore di Sanità, Rome, Italy; ^5^Institute of Molecular Biology and Pathology, CNR, Rome, Italy

**Keywords:** HIV-1, macrophage, CCL2 (MCP-1), innate response, RNA-sequencing, transcriptional profile, miR-155, NF-κB

## Abstract

Macrophages are key targets of human immunodeficiency virus type 1 (HIV-1) infection and main producers of the proinflammatory chemokine CC chemokine ligand 2 (CCL2), whose expression is induced by HIV-1 both *in vitro* and *in vivo*. We previously found that CCL2 neutralization in monocyte-derived macrophages (MDMs) strongly inhibited HIV-1 replication affecting post-entry steps of the viral life cycle. Here, we used RNA-sequencing to deeply characterize the cellular factors and pathways modulated by CCL2 blocking in MDMs and involved in HIV-1 replication restriction. We report that exposure to CCL2 neutralizing antibody profoundly affected the MDM transcriptome. Functional annotation clustering of up-regulated genes identified two clusters enriched for antiviral defense and immune response pathways, comprising several interferon-stimulated, and restriction factor coding genes. Transcripts in the clusters were enriched for RELA and NFKB1 targets, suggesting the activation of the canonical nuclear factor κB pathway as part of a regulatory network involving miR-155 up-regulation. Furthermore, while HIV-1 infection caused small changes to the MDM transcriptome, with no evidence of host defense gene expression and type I interferon signature, CCL2 blocking enabled the activation of a strong host innate response in infected macrophage cultures, and potently inhibited viral genes expression. Notably, an inverse correlation was found between levels of viral transcripts and of the restriction factors APOBEC3A (apolipoprotein B mRNA editing enzyme catalytic polypeptide-like 3 A), ISG15, and MX1. These findings highlight an association between activation of innate immune pathways and HIV-1 restriction upon CCL2 blocking and identify this chemokine as an endogenous factor contributing to the defective macrophage response to HIV-1. Therapeutic targeting of CCL2 may thus strengthen host innate immunity and restrict HIV-1 replication.

## Introduction

While CD4^+^ T cells are the primary targets of human immunodeficiency virus type 1 (HIV-1), macrophages are also infected *in vivo* by R5- and dual-tropic viruses (1, 2). Although the precise contribution of these cells to the pathogenesis of HIV-1 infection is still a matter of debate, their increased resistance to virus-induced cytopathic effect and reduced susceptibility to some antiretroviral drugs suggest they may contribute to residual viremia under combined antiretroviral therapy (cART). Recent discoveries in humanized mice and non-human primates models have indeed highlighted macrophage involvement in both viral persistence and development of either HIV-1 or simian immunodeficiency virus infection–associated comorbidities (3–7). Macrophages may thus represent an obstacle to cure HIV-1 infection and efforts defining the mechanisms and factors controlling HIV-1 replication in these cells may aid devising new treatments to interfere with viral persistence.

Innate immune responses are key determinants of the outcome of HIV-1 infection and influence critical events at the earliest stages of acute infection, to determine the rate of virus replication and spreads (8). Macrophages are potent cells of the innate immune system that initiate and regulate wide-ranging immunological responses. A body of evidence suggests that the role of macrophages in cellular host defense may be compromised by HIV-1 infection, which also appears to be ineffective in triggering innate immune activation in these as well as in other cells (9, 10). By interfering with innate responses, HIV-1 can circumvent host antiviral signaling and establish persistent viral reservoirs. Understanding how these protective responses are blocked in physiologically relevant models of HIV-1 infection and whether and how these defects can be reversed is therefore of great importance for developing novel therapeutic strategies aimed at eradicating the HIV-1 reservoir.

CC chemokine ligand 2 [CCL2; formerly monocyte chemotactic protein-1 (MCP-1)] is a main inflammatory chemoattractant directing the mobilization and homing of monocytes/macrophages and effector T lymphocytes to sites of inflammation. Elevated expression of CCL2 and of its receptor CC chemokine receptor 2 (CCR2) is found in diseases characterized by great numbers of infiltrating leukocytes and chronic inflammation (11). High CCL2 and CCR2 levels in HIV-1–infected individuals undergoing cART are tightly linked to increased inflammation and immune activation as well as to the development of comorbidities (12). CCL2 is produced by a variety of cell types, with monocytes/macrophages representing the major source among leukocytes. We previously reported that the expression of this chemokine was up-regulated during monocyte differentiation to macrophages (13) and further increased upon HIV-1 infection (14) or exposure to viral proteins (15–18). Induction of CCL2 expression was also reported by others (19). In monocyte-derived macrophages (MDMs), CCL2 promoted viral replication, as demonstrated by the finding that its blocking by neutralizing antibody (Ab) inhibited HIV-1 replication through impairment of viral DNA accumulation (14, 20). This effect did not involve the activity of the macrophage host restriction factor SAM and HD domain containing deoxynucleoside triphosphate triphosphohydrolase 1 (SAMHD1), while it was associated with an increased expression of the host restriction factor apolipoprotein B mRNA editing enzyme catalytic polypeptide-like 3A (APOBEC3A) (20).

In this study, we exploited a whole-genome transcriptome profiling approach to deeper characterize the cellular pathways and factors modulated by CCL2 blocking in both uninfected and HIV-1–infected macrophages and potentially involved in the restriction of viral replication. Our results reveal that the macrophage innate response to HIV-1 can be strengthened by blocking the chemokine CCL2. These findings are relevant for the development of novel therapeutic strategies aimed at eradicating the HIV-1 reservoir.

## Materials and Methods

### Ethics Statements

Healthy donor Buffy coats were obtained from Centro Trasfusionale–Sapienza University of Rome not specifically for this study. Informed consent was not asked because data were analyzed anonymously. Data from healthy donors were treated by Centro Trasfusionale according to the Italian law on personal data management “Codice in Materia di Protezione dei dati Personali” (Testo unico D.L. June 30, 2003 n. 196).

### Monocytes Isolation and Differentiation to MDMs

Monocytes were isolated from the peripheral blood of healthy donors by Ficoll–Paque density centrifugation followed by immunomagnetic selection using CD14^+^ microbeads (MACS monocyte isolation kit, Miltenyi Biotec) according to manufacturer’s instructions. This procedure yields a 95% to 98% pure population of monocytes, as assessed by fluorescence-activated cell sorter analysis of lineage-specific surface markers (CD14, CD3, CD56, CD19, and CD1a). Freshly isolated monocytes were seeded in 48-well cluster plates at 1 × 10^6^ cells per well in 1 mL of endotoxin-free IMDM (Lonza) supplemented with 2 mM L-glutamine, 2 mM penicillin/streptomycin, and 10% fetal bovine serum (Hyclone) and cultured for 6 days to allow differentiation to MDMs without the addiction of growth factors. The medium was then replaced with 0.5 mL fresh medium, and MDMs were treated with a rabbit polyclonal Ab directed against CCL2 as well as a control Ab (PeproTech) at the concentration of 2.5 μg/mL as previously reported (14, 20). Lipopolysaccharide contamination of the anti-CCL2 Ab was excluded by checking its endotoxin activity by the Kinetic-QCL Kinetic Chromogenic Limulus amebocyte assay (Lonza; detection limit, 0.00500 endotoxin U/mL). The endotoxin content determined was <0.1 endotoxin U/mL.

### HIV-1 Infection

Monocyte-derived macrophages were treated with anti-CCL2 or control Ab for 4 h and then infected with 3,000 tissue culture infectious dose (TCID_50_) per well of the CCR5-dependent HIV-1_BaL_ strain pelleted virus (Advanced Biotechnologies), corresponding to a MOI ≈ 0.03. After 2 h, cells were washed and maintained in complete medium either in the presence or in the absence of anti-CCL2 or control Ab as previously reported (14, 20).

### RNA-Sequencing and Data Analysis

Three RNA sequencing (RNA-seq) datasets were generated using MDMs obtained from 8 different donors choose among 12 based on RNA quality (Supplementary Figure S1). Total RNA samples were isolated with the RNeasy Mini kit (Qiagen) following the manufacturer’s instructions. RNA concentration and integrity were analyzed by a NanoDrop 2000 (Thermo Fisher Scientific) and a TapeStation (Agilent). All samples used for sequencing had an A280/260 value ≥2.0 and an RNA integrity number ≥8. Total RNA was subjected to poly (A) selection followed by reverse transcription. For datasets 1 (donors 6, 7, and 8) and 3 (donors 9, 11, and 12), RNA-seq libraries were created with the Illumina Neoprep instrument and sequenced using the Illumina Hiseq 4000 platform. Samples were sequenced two times in multiplexed lanes and reads of the same sample from the two runs were pooled together. For dataset 2 (donors 2 and 4), RNA-seq libraries were created with the Illumina Truseq RNA sample pre kit and sequenced using the Illumina Hiseq 2500 platform. Samples were sequenced three times in multiplexed lanes. Reads of the same sample from three runs were pooled together. Tophat (21) (version 2.0.6) together with bowtie (version 0.12.8) were used to align reads to human genome GRCh37/hg19 with Ensembl 75 gene annotation. Only uniquely mapped reads were used to count reads aligned to each gene. Reads were quantified by htseq-count (22) (version 0.5.3p9) with Ensembl 75 gene sets. Gene differential expression analysis was performed using DESeq2 (23) (version 1.4.5). Genes that had no reads across all samples were discarded. Genes with an adjusted *p* value (*p*_adj_) of less than 0.1 were classified as significantly differentially expressed, and those with more than a twofold change (FC) in expression were used for functional analysis, unless otherwise indicated. Tophat2 was used to align the RNA-seq reads that failed to map to the human genome to the HIV-1BaL genome (accession no. AB221005) to obtain estimates of viral gene expression.

The Database for Annotation, Visualization and Integrated Discovery (DAVID; version 6.8) was used to perform functional annotation and functional annotation clustering of differentially expressed genes (DEGs) (24, 25). A false discovery rate (FDR) of 0.05 was selected as the cutoff criterion for functional annotation. The classification stringency setting used for functional annotation clustering was medium with default setting for function grouping, except for EASE, which was lowered to 0.00003 to reduce inclusion of non-significant terms into the clusters. Annotation Clusters of significantly overrepresented groups with terms having an FDR < 5% were accepted for further consideration. Transcriptional Regulatory Relationships Unraveled by Sentence-based Text mining (TRRUST version 2.0), a manually curated database of human transcriptional regulatory networks, was used to obtain the candidate key transcription factors (TFs) regulating a set of DEGs (26).

Hierarchical clustering and heatmaps were done using the pheatmap package in R software (version 1.0.12)^1^. ClustVis^2^ was used to perform principal component analysis (PCA) (27). Box plots were generated using the boxplot function in R (version 3.6.2) (28)^3^. Volcano plots were generated using EnhancedVolcano in R software (version 1.5.4)^4^. Venn diagrams were generated using the online tools^5^ (29). Cytoscape (version 3.7.2.)^6^ was employed to visualize expression data in specific molecular networks (30). GraphPad Prism version 8.4.1 (GraphPad Software, Inc.) and Excel (Microsoft Corp.) were used for statistical analyses and graphs drawing.

### MicroRNA Expression Profiling and Data Analysis

Total RNA was isolated with the miRNeasy mini kit (Qiagen) that allows detecting small RNAs, following the manufacturer’s procedure. The extracted RNA (500 ng) was retrotranscribed by using the TaqMan Micro-RNA Reverse Transcription Kit and the Megaplex RT Primers (Applied Biosystems). TaqMan Array MicroRNA A Card v2.0 was used to analyze the expression of multiple miRNA sequences. The miRNA polymerase chain reaction (PCR) array is a set of optimized real-time PCR assays, in 384-well plates, which allows simultaneous assays for pathway-focused sets of human miRNA sequences. The TaqMan Array was processed by the ViiA 7 Real Time PCR System and the results were analyzed by the Thermo Fisher Cloud (Applied Biosystems). Moreover, linear structural equation models (SEMs) with miRNAs FCs as dependent variables, type of treatment as independent variable, and donor clustered standard errors was carried out by the maximum likelihood method. This analysis was carried out in Stata 13 (StataCorp LLC). Hierarchical clustering of miRNA array data was performed using SPSS version 24 (IBM Corp.). TargetScan (31), a web server that search for predicted miRNA targets, was used to predict targets of miR-155.

### Analysis of miR-155 by Quantitative RT-PCR

The expression profile of miR-155 was confirmed using quantitative RT-PCR (qPCR) performed on the same RNA samples used in the array. Total RNA (10 ng) was retrotranscribed using the TaqMan Reverse Transcription Kit (Applied Biosystems), followed by qPCR amplification with the Universal PCR Master Mix (Applied Biosystems) on an ABI Prism 7500 PCR cycler (Applied Biosystems). The TaqMan Small RNA assay for hsa-miR-155-5p (assay ID 002623; same primers and probe present in the TaqMan Card) was used. As endogenous control, primers with TaqMan probe for U6 snRNA (assay ID 001973) were used. To assess the role of nuclear factor κB (NF-κB) signaling in miR-155 expression, MDMs from three additional donors were treated with the inhibitor of I kappa B kinase BMS-345541 (10 μM; Sigma–Aldrich) for 1 h prior to anti-CCL2 Ab (2.5 mg/mL) exposure for 4 h. Total RNA was extracted with the Total RNA Purification Plus Micro Kit (Norgen), and then retro-transcribed and amplified as described above. The inhibitor did not exhibit any toxicity at the used concentration, as assessed by MTT assay (data not shown).

### Analysis of Cellular Genes by qRT-PCR

The expression profile of selected cellular genes was confirmed using qPCR performed on RNA samples isolated from two donors employed in RNA-seq and five additional donors. For these latter samples, total RNA was isolated with the total RNA Purification Plus Micro Kit (Norgen). RNA (400 ng) was retrotranscribed into cDNA by using poly d(N)6 (GE Healthcare) and real-time PCR was performed on an ABI Prism 7500 PCR cycler (Applied Biosystems) in a 20 μL reaction mix containing 1 μL of cDNA. Validated PCR primers and TaqMan MGB probe (6FAM-labeled) for NFKB1 (Hs.PT.58.38905484), NFKBIA (Hs.PT.58.15498666.g), STAT1 (Hs.PT.58.15049687), IRF1 (Hs.PT.58.26847423), IRF7 (Hs.PT.58.24613215.g), CCR5 (Hs.PT.58.3437570), CCL3 (Hs.PT.58.27485430.g), CCL4 (Hs99999148_m1), CCL5 (Hs.PT.58.1724551), APOBEC3A (Hs.PT.58.45326850), ISG15 (Hs.PT.58.39185901.g), and MX1 (Hs.PT.58.40261042) were used (NFKB1, NFKBIA, STAT1, IRF1, IRF7, CCR5, CCL3, CCL5, APOBEC3A, ISG15, and MX1 from Integrated DNA Technologies, CCL4 from Applied Biosystems). As endogenous control, primers and TaqMan probe for human GAPDH (Hs.PT.49a.2918858.g; Integrated DNA Technologies) were used. Thermal cycler conditions were previously reported (17). Relative quantification was performed by using the comparative Ct method as previously described (17).

### Analysis of HIV-1 Transcripts by qRT-PCR

The expression profile of selected HIV-1 genes was confirmed using qPCR performed on RNA samples isolated from one donor employed in RNA-seq (dataset 3) and two additional donors. For these latter samples, total RNA was isolated with the Total RNA Purification Plus Micro Kit (Norgen). Residual genomic DNA was eliminated using ezDNase (Invitrogen) according to the manufacturer’s procedures. RNA (500 ng) was retrotranscribed into cDNA using poly d(N)6 (GE Healthcare) and the Superscript II reverse transcriptase enzyme (Life Technologies). RT-minus controls were included in each experiment to provide a negative control for subsequent PCR reactions. Real-time PCR was performed on an ABI Prism 7500 PCR cycler (Applied Biosystems) in a 20 μL reaction mix containing 1 μL of cDNA. Specific primers and probe for the HIV-1 gag gene were previously reported (32), and those for the HIV-1 env gene were as follow: forward GTCTCTCTCTCCACCTTCTTCT, reverse TAGGCAGGGATACTCACCATTA, probe/56-FAM/TCGTTTCAG/ZEN/ACCCACCTCCCAG/3IABkFQ/. Primers with TaqMan probe for human GAPDH (Hs.PT.49a.2918858.g; Integrated DNA Technologies) were used as endogenous control. Thermal cycler conditions were as follows: 1 × 20 s at 95°C followed by 40 cycles of denaturation (3 s at 95°C) and extension (30 s at 57°C). Each sample was run in triplicate to ensure accurate FC estimation. Relative gene expression was calculated by the ΔΔCt method as previously reported (17).

## Results

### Whole Transcriptome Sequencing Analysis of MDMs Upon CCL2 Neutralization

**FIGURE 1:**
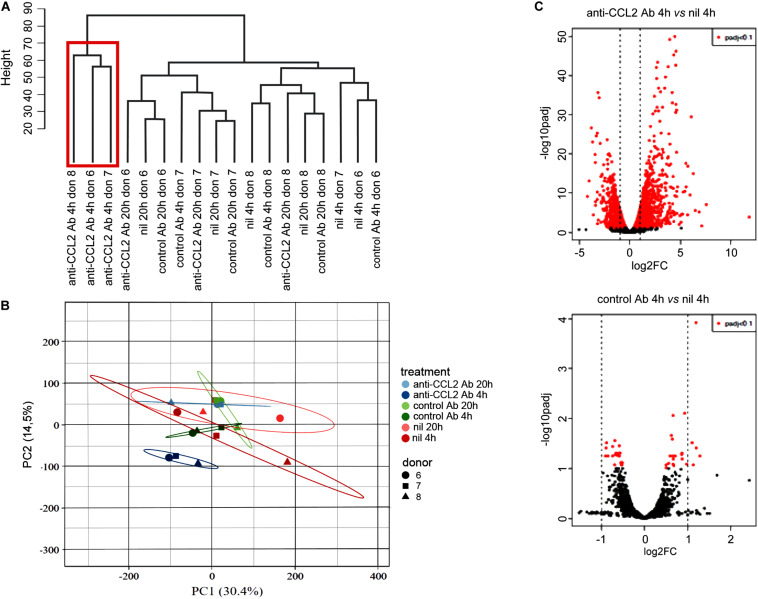
Effect of CCL2 neutralization on the MDM transcriptome. MDMs obtained from three donors were treated or not with anti-CCL2 or control Ab (2.5 μg/mL) for 4 or 20 h. Total RNA was extracted, subjected to poly(A) selection followed by reverse transcription, generation of cDNA libraries, and sequencing (dataset 1). **(A)** Hierarchical clustering of RNA-seq data. **(B)** Principal component analysis of RNA-seq data. Unit variance scaling was applied to rows; SVD with imputation was used to calculate principal components; *n* = 18 data points. Prediction ellipses are such that with probability of 0.95, a new observation from the same group will fall inside the ellipse. **(C)** Volcano plot representations showing significances versus fold changes of differential expression analysis of genes in the anti-CCL2 Ab 4 h versus nil 4 h and control Ab 4 h versus nil 4 h comparisons. The red points mark the significantly differentially expressed genes (*p*_adj_ < 0.1), and the vertical lines indicate the twofold change threshold.

Genome-wide transcriptome analysis using RNA-seq was employed to characterize the effect of CCL2 neutralization on macrophage global gene expression. MDMs obtained from three different donors were exposed to anti-CCL2 or control Ab and compared to untreated samples (*n* = 18 total samples; Supplementary Figure S1; dataset 1). Cells were collected at 4 and 20 h posttreatment, and total RNA was isolated, subjected to poly (A) selection followed by reverse transcription, generation of cDNA libraries, and sequencing. The 18 samples were sequenced in two multiplexed lanes. Reads from the two different lanes were pooled together. The descriptive statistics of the RNA-seq reads of dataset 1 are shown in Supplementary Figure S2. We obtained an average of 39 million reads per sample, with an average of 95% of reads mapped to the human genome, of whom 80% to 85% were uniquely mapped (Supplementary Figure S2A). Among uniquely mapped reads, only 10% were assigned to intragenic regions (Supplementary Figure S2B), and an average of 33 million reads were assigned to protein-coding genes (Supplementary Figure S2C). Cluster analysis of dataset 1 showed that MDMs treated with anti-CCL2 Ab for 20 h grouped with control samples (untreated, nil, and control Ab–treated cells), whereas MDMs exposed to anti-CCL2 Ab for 4 h clustered together apart from control samples (Figure 1A), indicating a stronger response upon 4 h of CCL2 neutralization and a limited effect of control Ab. PCA of RNA-seq data confirmed the clustering of MDMs exposed to anti-CCL2 Ab for 4 h (Figure 1B). Principal component 1 (PC1) and PC2, respectively, explained 30.4% and 14.5% of the total variance in the RNA-seq data.

Figure 1C shows volcano plot representations of DEGs in the anti-CCL2 Ab 4 h versus nil 4 h and control Ab 4 h versus nil 4 h comparisons. Differential expression analysis using a threshold *p*_adj_ < 0.1 and log2 FC (log2FC) ≥ 1 or ≤ -1 identified a total of 1,558 transcripts in MDMs exposed to anti-CCL2 Ab for 4 h, of which 844 and 714 were, respectively, up- and down-regulated. Complete lists of these genes are reported in Supplementary Tables S1, S2. Of the 844 up-regulated genes, 5 (IL7R, GBP1, IFI44L, MET, and PDPN) were also induced by control Ab treatment. However, since the FC of anti-CCL2 Ab with respect to control Ab was ≥2, these genes were not excluded from subsequent analyses. By contrast, control Ab treatment did not down-modulate any gene.

In order to gain insight into the biological meaning behind the large number of DEGs in anti-CCL2-treated MDMs, we performed an enrichment analysis using Gene Ontology (GO) terms (biological processes, BPs; molecular functions, MFs; cellular components, CCs), functional categories (up keywords) and pathway (KEGG) databases using the DAVID online tool. As shown in Supplementary Table S3, 34 BPs, 4 MFs, 10 CCs, 17 up keywords, and 10 KEGG pathways were significantly enriched (FDR < 0.05) in the list of up-regulated genes, whereas only one BP and two up-keywords were significantly enriched in the down-regulated transcripts. The up-regulated genes were enriched for immune pathways, intracellular signaling cascades associated with the immune system, and leukocyte migration processes, while GO:0043547∼positive regulation of GTPase activity was the only biological process enriched in the down-regulated genes.

### CCL2 Neutralization Activates the Innate Antiviral Defense Response in MDMs

**FIGURE 2:**
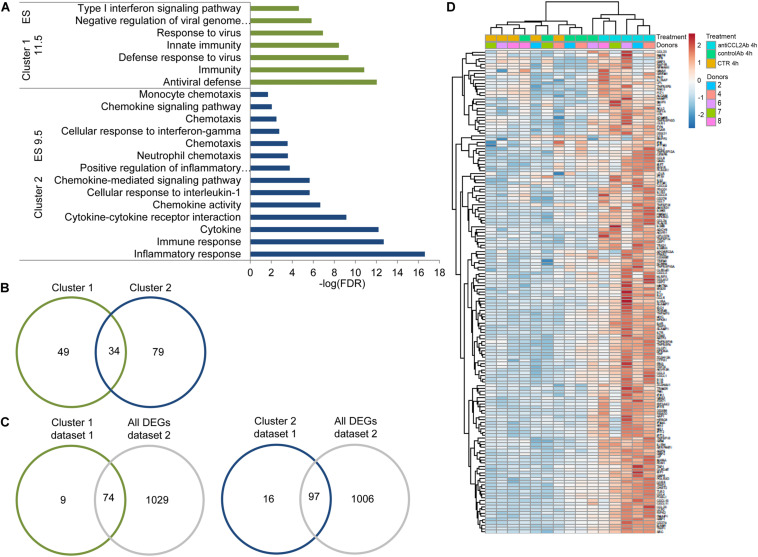
CCL2 neutralization activates the innate antiviral defense response in MDMs. **(A)** Functional annotation clustering by DAVID of the genes up regulated in MDMs exposed to anti-CCL2 Ab for 4 h (dataset 1). **(B)** Venn diagram showing the overlay of genes included in clusters 1 and 2. **(C)** Venn diagrams showing the overlay of genes included in cluster 1 or 2 (dataset 1) with dataset 2. **(D)** Heatmap with unsupervised hierarchical clustering of the 162 genes included in clusters 1 and 2 (datasets 1 and 2).

To gain an overview of the nature of the functional pathways enriched by CCL2 neutralization, we performed functional annotation clustering of the up-regulated DEGs using DAVID. This tool clusters related groups and orders these clusters according to their significance (as determined by their EASE scores, a modified Fisher exact *p* value). As shown in Figure 2A and Supplementary Table S4, this analysis revealed two clusters of up-regulated genes, which included items related to antiviral defense/innate immunity (cluster 1, enrichment score 11.5, 83 genes) and immune/inflammatory response (cluster 2, enrichment score 9.5, 113 genes). Overall, terms in the clusters underscored the regulation and activation of the immune system following CCL2 neutralization. The two clusters accounted for 21 of the 75 significantly enriched terms in the up-regulated genes. Since there was a partial overlap among the genes in each cluster, the total number of transcripts included in the two clusters was 162 (Figure 2B and Table 1), which accounted for 19% of all the up-regulated genes. Clusters 1 and 2 included, respectively, 49 and 79 unique genes (Figure 2B). To confirm these results, we compared the lists of genes included in clusters 1 and 2 with a different RNA-seq dataset generated using MDM samples obtained from two different donors and exposed to anti-CCL2 or control Ab for 4 h (Supplementary Figure S1; dataset 2). The descriptive statistics of the RNA-seq reads of dataset 2 are shown in Supplementary Figure S3. Cluster analysis of dataset 2 confirmed that MDMs exposed to anti-CCL2 Ab for 4 h clustered together apart from control samples (Supplementary Figure S4A). A volcano plot representation of DEGs in the anti-CCL2 Ab 4 h versus nil 4 h comparison in dataset 2 is shown in Supplementary Figure S4B. The overlap among the two lists of genes revealed that most of the genes in the clusters (89% and 86% in clusters 1 and 2, respectively) were up-regulated also in dataset 2 (Figure 2C). In particular, 45 and 68 of the 49 and 79 unique genes in cluster 1 (92%) and 2 (86%), respectively, were in common with dataset 2 (Table 1). Strikingly, the functional annotation clustering of the up-regulated genes at 4 h of anti-CCL2 Ab treatment in dataset 2 showed that the two clusters with the highest significance were similar to those obtained with dataset 1 and included items related to antiviral defense, innate immunity, immune response, and inflammatory response (Supplementary Table S4). A heatmap with the 162 genes included in the two clusters, generated with datasets 1 and 2, is shown in Figure 2D. Interestingly, 18 of these genes were up-regulated (*p*_adj_ < 0.1; log2FC ≥ 1) also in MDMs treated with anti-CCL2 Ab for 20 h (Table 1), and the functional annotation clustering using this list of genes generated two clusters with terms related to antiviral defense, innate immunity, immune response and inflammatory response (Supplementary Table S5). Notably, cluster 1 (Figure 2A) comprised several interferon-stimulated genes (ISGs), such as DDIT4, GBP1, IFI44L, IFIT3, IRF1, IRF7, ISG20, OAS3, OASL, and the restriction factor coding genes APOBEC3A, EIF2AK2, GBP5, HERC5, IFITM1, IFITM3, ISG15, MX1, RSAD2, and TRIM25, some of which (APOBEC3A, IFITM1, ISG15, and RSAD2) were also up-regulated after 20 h of anti-CCL2 Ab exposure (Table 1). Cluster 2 included genes encoding chemokines and cytokines (some of which were in common with cluster 1) as well as members of the tumor necrosis factor superfamily (TNFSF) and TNF receptor superfamily (TNFRSF), which play crucial roles in both innate and adaptive immune responses.

**TABLE 1:** List of genes included in clusters 1 and 2 of Figure 2A.

Cluster	Genes
1	ALCAM, APOBEC3A*, BATF3, BCL2, BNIP3, C1S, CD300E, CFB, CLEC4D, CLEC4E, CTPS1, DDIT4, DDX21, DDX58, EIF2AK2, FYN, GBP1, GBP3, HERC5, HMGA1, IDO1*, IFI44L, IFIH1, IFIT2, IFIT3*, IFIT5, IFITM1*, IRF1, IRF7, ISG15*, ISG20, KCNN4, MX1, MX2, OASL, PDCD1, PLSCR1, PMAIP1, PML, POLR3D, RIPK2, RSAD2*, SLAMF1, SLAMF7, TAP1, TBX21, TRIM25, XAF1, ZC3HAV1
1 and 2	C3, CCL4, CCL5, CCL8, CD40, CSF1, CXCL10*, CXCL9, ETS1, FFAR2*, FOSL1, GBP2, GBP5, IFI6, IFITM3, *IL1RAP, IL27, IL2RA, IL36B, IL36G, IL36RN, IL4R, LYN*, NLRP3, OAS3*, OLR1, OTUD7B, SRC, STAT1, TLR2, TNF, TNFSF18, *TNIP1, ZC3H12A*
2	*ACKR4, ACVR1, ACVR2A, ADCY6*, ADGRE2, ADGRE5, *ANKRD1, AQP9*, BMP6, CCL2*, CCL20, CCL23*, CCL24*, CCL3, CCL4L2, CCL7, CCR5, CD274, CD276, CEBPB, CHST2, CLCF1, CSF2, CSF3, CXCL1, CXCL11, CXCL3, CXCL5, CXCL8, EBI3*, EDN1, ENPP2, *FCAR, FLT1, GEM*, GNAI1, GREM1, HIF1A, ICAM1, IL1A, IL1B, IL1R2*, IL1RN, IL32, IL7, IL7R, INHBA, KDM6B, LCP2, LDLR, LIF, LPL, MYC, NAMPT, NFKB1, NFKBIA, NFKBID, PDE4B, PDGFRB, PLAUR, PNP, RAC2, RAP1B, SEMA3C, SERPINE1*, TNFAIP3, TNFRSF10A, TNFRSF10D, TNFRSF12A, TNFRSF18*, TNFRSF4*, TNFRSF9*, TNFSF14, TNFSF15, TNIP2, TNIP3, TREM1, WNT5A, ZEB1

Genes in italic are in common with dataset 2. *Genes up-regulated also after 20h of anti-CCL2 Ab exposure.

### An NF-κB/miR-155 Regulatory Network Triggers MDM Immune Responses Upon CCL2 Neutralization

We next aimed at identifying the key regulatory interactions among TFs and microRNAs (miRNAs) underlying the modulation of the immune response by CCL2 neutralization. We first analyzed the list of the 162 genes included in clusters 1 and 2 (Figure 2A) using the TRRUST database to identify the TFs associated with the regulation of these transcripts following exposure to anti-CCL2 Ab. The top 10 ranked TFs are shown in Table 2. Interestingly, RELA and NFKB1 represented the two TFs having maximum FDR and coverage of DEGs (43 and 42 genes, respectively), indicating the activation of the canonical NF-κB pathway following CCL2 blocking. NFKB1 and RELA targets were almost completely overlapping, except for STAT1 that is target only of RELA. An enrichment for NFKB1 and RELA targets was found in the genes included in both clusters, although more target genes were found in cluster 2 and most of those in cluster 1 were in common with cluster 2 (Table 2).

**TABLE 2:** Top ten transcription factor signatures enrichment by TRRUST of the 162 genes upregulated by anti-CCL2 Ab treatment at 4h included in clusters 1 and 2 of Figure 2A.

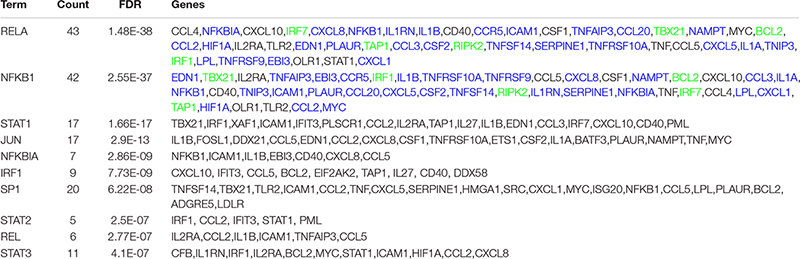

*RELA and NFKB1 targets present in cluster 1 or 2 only are respectively in green and blue.*

We then performed miRNA expression profiling of MDMs obtained from three different donors and exposed to anti-CCL2 or control Ab for 4 h (Supplementary Figure S1). Hierarchical clustering and PCA analysis showed no clear separation between the different groups of samples (Supplementary Figures S5A,B), indicating a significant variability among donors in the response to CCL2 neutralization. Differential expression analysis using a threshold *p* < 0.05 and FC > 2 identified one significantly down-regulated (miR-501-3p) and 4 significantly up-regulated (miR-155-5p, miR-146b-5p, miR-221-3p, miR-200a-3p) miRNAs in anti-CCL2 Ab 4 h versus nil 4 h (Supplementary Figure S5C). Differential expression of miR-155-5p and miR-221-3p, was also found in the anti-CCL2 Ab 4 h versus control Ab 4 h comparison (Supplementary Figure S5C). FC and *p* value of the differentially expressed miRNAs are showed in Table 3. Furthermore, miR-155 up-regulation was also confirmed by SEM analysis (FC = 16.3, *p* = 0.018).

**TABLE 3:** Differentially expressed miRNAs following 4h of anti-CCL2 Ab treatment of MDMs.

	anti-CCL2 Ab vs nil		anti-CCL2 Ab vs control Ab	
miRNA	FC	*p*-value	FC	*p*-value
**Up-regulated**				
hsa-miR-200a-3p	22.40	0.022	n.s.	n.s.
hsa-miR-155-5p	19.10	0.009	10.52	0.026
hsa-miR-146b-5p	5.68	0.040	n.s.	n.s.
hsa-miR-221-3p	4.28	0.010	3.88	0.035
Down-regulated				
hsa-miR-501-3p	0.21	0.027	n.s.	n.s.

**FIGURE 3:**
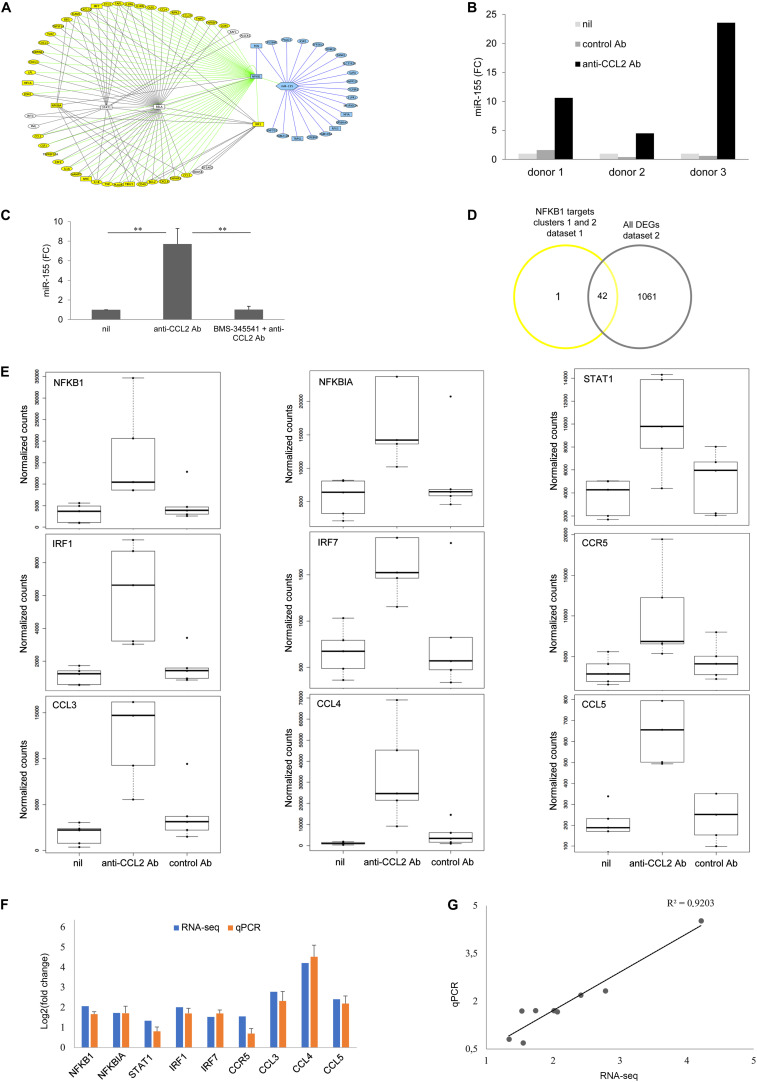
CCL2 neutralization triggers an NF-κB/miR-155 regulatory network to regulate immune responses. **(A)** Predicted interaction network showing the regulatory relationships of miR-155, NF-κB, and their target genes in clusters 1 and 2. TFs, gene, and miR are represented as rectangular, oval, and hexagonal nodes, respectively. The edge colors represent different relationships: blue for the repression of miR-155 and FOS to genes or NFKB1, respectively; green for the regulation of NFKB1 to genes or miR-155; and black for the regulation of other TFs to genes or TFs. Nodes in yellow correspond to NFKB1 targets, and nodes in blue correspond to miR-155 targets. **(B)** Analysis by qPCR of miR-155 levels in MDMs treated or not with anti-CCL2 Ab for 4 h. The results obtained with three different donors are shown. **(C)** Effect of NF-κB inhibition on miR-155 level. MDMs were treated with BMS-345541 (10 μM) for 1 h prior to anti-CCL2 Ab (2.5 μg/mL) exposure for 4 h. Total RNA was then extracted and retrotranscribed, and miR-155 was amplified by qPCR. Data represent mean values (+SE) of the results obtained with three donors. **p < 0.01 (anti-CCL2 Ab vs. control; BMS-345541 + anti-CCL2 vs. anti-CCL2 Ab). **(D)** Venn diagram showing the overlay between NFKB1 target genes in clusters 1 and 2 (dataset 1) with dataset 2. **(E)** Box plots of DESeq2 normalized counts of NFKB1, NFKBIA, STAT1, IRF1, IRF7, CCR5, CCL3, CCL4, and CCL5 transcripts following 4-h exposure to anti-CCL2 Ab. Each condition has five biological replicates (datasets 1 and 2). **(F)** Analysis by qPCR of the levels of NFKB1, NFKBIA, STAT1, IRF1, IRF7, CCR5, CCL3, CCL4, and CCL5 in MDMs treated or not with anti-CCL2 Ab for 4 h. The *y* axis represents the log2(fold change) values derived from RNA-seq (blue) and qPCR (red). **(G)** Correlation between qPCR and RNA-seq data for the genes reported in panel F. The correlation of the fold changes was calculated by the Pearson correlation coefficient. Results of panels **(F,G)** are based on RNA-seq data from dataset 1 and qPCR data from four donors.

We thus focused on miR-155 and its correlation with the identified TF signatures. It was previously shown that NF-κB activates miR-155 expression, which then acts as an amplifier and positive regulator to ensure robust and strong NF-κB activity (33). To investigate the functional significance of miR-155 up-regulation, we performed an integrated analysis to build a miR-155-mRNA interaction network. TargetScan predicted a total of 556 genes as potential targets of miR-155 based on the complementary target sequence. The prediction was further filtered by inverse correlation between miR-155 and transcripts expression, leading to the identification of 21 genes among those down-regulated by CCL2 blocking (Figure 3A). Interestingly, one of the down-regulated genes target of miR-155 was FOS, a TF identified as repressor of NFKB1 transcription in the TRRUST database (Table 4). We thus used Cytoscape to construct a positive NF-κB–miR-155 regulatory network involving miR-155, NF-κB, miR-155 targets, and NFKB1/RELA regulated targets in the clusters (Figure 3A). To validate this network, we first employed qPCR to confirm miR-155 up-regulation in anti-CCL2 Ab–treated MDMs. As shown in Figure 3B, qPCR performed on samples from the same donors used in the array confirmed miR-155 increase following exposure to anti-CCL2 Ab. We then assessed the effect of NF-κB inhibition on anti-CCL2 Ab-mediated up-regulation of miR-155. The results of these experiments further confirmed miR-155 up-regulation following CCL2 neutralization in three different donors and demonstrated that pre-treatment of MDMs with BMS-345541, a highly selective inhibitor of I kappa B kinase that blocks NF-κB–dependent transcription, completely inhibited miR-155 up-modulation by anti-CCL2 Ab treatment (Figure 3C). We then confirmed NFKB1 and NFKB1/RELA target genes differential expression by using dataset 2 and found that 42 (out of 43) of these genes (including NFKB1 itself) were also present in this independent dataset (Figures 3D,E). These genes comprised the TFs STAT1, IRF1 and IRF7 and the regulator of NF-κB activity NFKBIA, which are all part of the network of Figure 3A, as well as CCR5 and its ligands CCL3, CCL4, and CCL5, which play key roles in HIV-1 infection (Figure 3E). The differential expression of these genes was validated by qPCR employing samples from one of the donors used in RNA-seq (dataset 1) and three additional donors. As shown in Figures 3F,G, the qPCR data were highly concordant with the RNA-Seq data, with a correlation coefficient of 0.959 (*p* < 0.0001).

**TABLE 4:** Transcription factors that regulate NFKB1 by TRRUST.

TF	Mode of regulation	Reference (PMID)
APEX1	Activation	17045925
AR	Repression	18386814
BCL3	Repression	11387332
BCL3	Unknown	14573596, 7896265
BCL6	Repression	15611242
BTF3	Repression	17312387
EGR1	Unknown	10671503
EP300	Unknown	9560267
ETV3	Repression	18025162
FHL2	Unknown	18752053
FOS	Repression	20188076
GATA3	Unknown	19686049
HDAC1	Unknown	24448807
HDAC9	Repression	15964798
HOXA9	Repression	18068911
HSF1	Unknown	18689673
LYL1	Repression	10023675
NFKB1	Unknown	19469019
NFKBIA	Activation	9500973
NFKBIA	Repression	11278471, 18606063, 20173029
NFKBIA	Unknown	15713458, 17148610
NR1H4	Activation	21364590
NR1I2	Repression	22248096
NR3C1	Repression	17016446
PARP1	Unknown	16026317
PIR	Unknown	14573596
RBPJ	Unknown	14570916
RELA	Activation	20596645
RELA	Unknown	19469019
RUNX1	Unknown	19686049
SP1	Activation	20538607, 9151857
SP3	Activation	20538607
SP4	Activation	20538607
SRSF1	Repression	19183244
TAL1	Repression	16778171
TNFAIP3	Repression	19124729
TNFAIP3	Unknown	24039598
TP53	Unknown	15988033
TRIM22	Unknown	21651891
USF1	Unknown	19686049
ZNF382	Repression	20682794

### CCL2 Neutralization Enables the Activation of Host Defense Genes in HIV-1–Infected MDM Cultures

The transcriptional profiling of MDMs exposed to anti-CCL2 Ab highlighted the induction of a strong innate defense response after 4 h of treatment. On this basis, we chose this pre-treatment time point in the subsequent RNA-seq experiments aimed at characterizing the outcome of CCL2 neutralization in HIV-1–infected macrophage cultures. MDMs obtained from three different donors were exposed to anti-CCL2 or control Ab for 4 h and then infected with HIV-1. Cells were collected at days 1 and 4 postinfection (p.i.). For each donor, a control untreated sample (nil) and a sample with HIV-1 in the absence of any treatment were used to assess the effect of the virus on the MDM transcriptome at both time points (*n* = 24 total samples; Supplementary Figure S1; dataset 3). These samples were sequenced in two multiplexed lanes and reads from the 2 different lanes were pooled together. The descriptive statistics of the RNA-seq reads of dataset 3 are shown in Supplementary Figure S6. We obtained an average of 35 million reads per sample, with an average of 95% of reads mapped to the human genome, of whom 80 to 85% were uniquely mapped (Supplementary Figure S6A). Among uniquely mapped reads, only 10% were assigned to intragenic regions (Supplementary Figure S6B), and an average of 30 million reads were assigned to protein-coding genes (Supplementary Figure S6C). Hierarchical clustering analysis of dataset 3 showed that similarity in expression profiles is largely determined by the donor of origin (i.e., the donor of origin is the primary source of heterogeneity), suggesting that the variation among donors in this dataset is stronger than the effect of HIV-1 infection or anti-CCL2 Ab treatment (Supplementary Figure S7A). This was confirmed by PCA analysis, which showed no clear separation between the different groups of samples (Supplementary Figure S7B).

**FIGURE 4:**
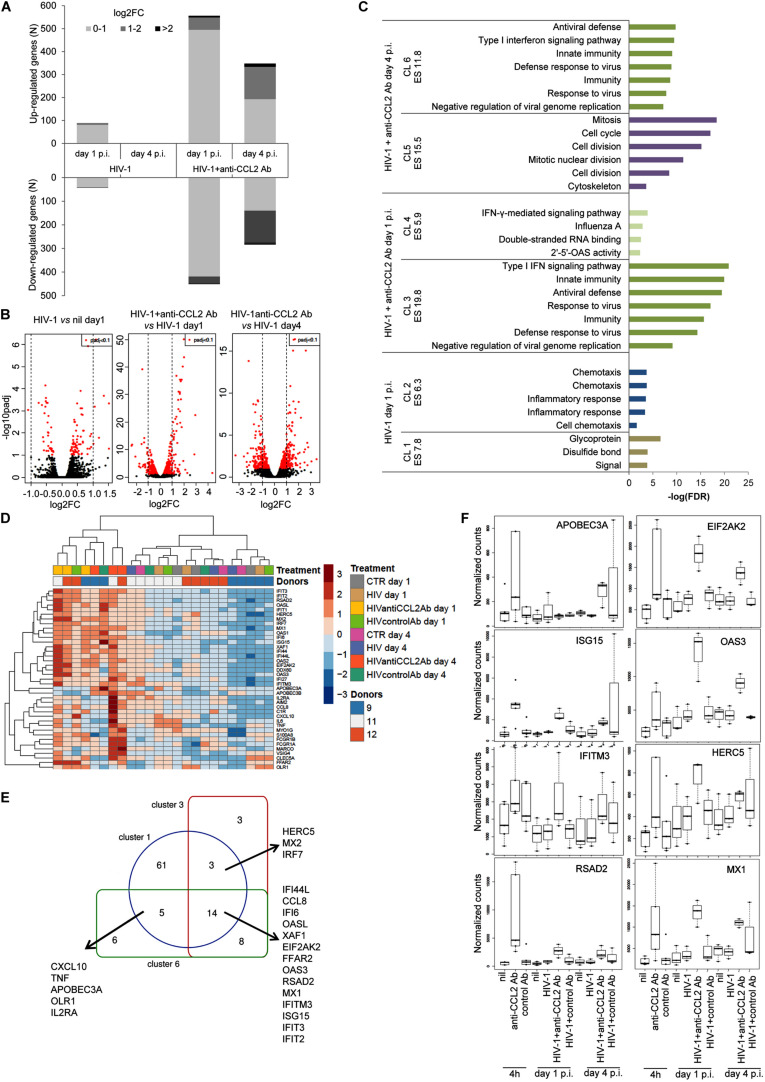
Effect of CCL2 neutralization on the HIV-1–infected MDM transcriptome. MDMs obtained from three donors were treated or not with anti-CCL2 or control Ab (2.5 μg/mL) for 4 h and then infected with HIV-1_BaL_. Total RNA was extracted at days 1 and 4 p.i., subjected to poly(A) selection followed by reverse transcription, generation of cDNA libraries, and sequencing (dataset 3). **(A)** Number of differentially expressed genes in HIV-1–infected MDMs exposed or not to anti-CCL2 Ab. **(B)** Volcano plot representations showing significances versus fold changes of differential expression analysis of genes in the HIV-1 day 1 versus nil day 1, HIV-1 + anti-CCL2 Ab day 1 versus HIV-1 day 1 and HIV-1 + anti-CCL2 Ab day 4 versus HIV-1 day 4 comparisons. The red points mark the significantly differentially expressed genes (*p*_adj_ < 0.1), and the vertical lines indicate the twofold change threshold. **(C)** Functional annotation clustering by DAVID of the genes up-regulated in HIV-1–infected MDMs exposed or not to anti-CCL2 Ab at days 1 and 4 p.i. For the HIV-1 + anti-CCL2 Ab condition, the graph reports the two clusters with the highest significance. **(D)** Heatmap with unsupervised hierarchical clustering of the 39 genes included in clusters 3 and 6. **(E)** Venn diagram showing the overlay of genes included in cluster 3 (HIV-1 + anti-CCL2 Ab day 1 p.i.), cluster 6 (HIV-1 + anti-CCL2 Ab day 4 p.i.), and cluster 1 of Figure 2A (anti-CCL2 Ab 4 h). **(F)** Box plots of DESeq2 normalized counts of APOBEC3A, EIF2AK2, OAS3, HERC5, IFITM3, ISG15, MX1, and RSAD2 transcripts in uninfected MDMs following 4-h exposure to anti-CCL2 Ab (datasets 1 and 2) and HIV-1–infected MDMs following exposure to anti-CCL2 Ab at days 1 and 4 p.i. (dataset 3). Each condition has three and five biological replicates for HIV-1–infected and uninfected MDMs, respectively.

As shown in Figures 4A,B, HIV-1 infection caused remarkably small changes to the host cell transcriptome. In particular, 132 genes were differentially expressed at day 1 p.i. (*p*_adj_ < 0.1), of which 89 and 43 were, respectively, up- and down-regulated, and only 8 of the former (NLRP12, PLA2G16, ANKRD22, GGT5, STAC, HSD11B1, CCL24, FPR2) and 2 of the latter (CCDC152 and RCN3) displayed a log2FC ≥ 1 or ≤–1, respectively (Supplementary Table S6). In contrast, we did not observe DEGs with *p*_adj_ < 0.1 at day 4 p.i. Conversely, the presence of anti-CCL2 Ab induced more pronounced changes in MDM transcriptional profile (Figures 4A,B). In particular, we identified a total of 1,007 (556 up-regulated and 451 down-regulated) and 632 (348 up-regulated and 284 down-regulated) DEGs (*p*_adj_ < 0.1) at days 1 and 4 p.i. in HIV-1–infected MDM cultures upon anti-CCL2 Ab treatment. Of these transcripts, 93 at day 1 p.i. (61 up-regulated and 32 down-regulated) and 299 at day 4 p.i. (155 up-regulated and 144 down-regulated) had a log2FC ≥ 1 or ≤ −1 (Supplementary Tables S7, S8). Furthermore, the functional annotation clustering analysis by DAVID of the 89 transcripts up-regulated by HIV-1 at day 1 p.i. showed enrichment of functionally related groups of genes involved in cell chemotaxis and inflammatory response (cluster 2, enrichment score 6.3, number of genes 16), but not for host defense gene expression and no evidence of type I interferon (IFN) signature (Figure 4C and Supplementary Table S9). Conversely, the functional annotation clustering of the 61 transcripts up-modulated by anti-CCL2 Ab treatment at day 1 p.i. revealed two clusters, with the most enriched one being related to antiviral defense and type I IFN signaling (cluster 3, enrichment score 19.8, 28 genes). A similar functional cluster was also found by analyzing the 155 genes induced by CCL2 neutralization at day 4 p.i. (cluster 6, enrichment score 11.8, 33 genes) (Figure 4C and Supplementary Table S9). Clusters 3 and 6 overall comprised 39 different genes, which are shown in the heatmap of Figure 4D. Figure 4E shows the overlay among cluster 3 (HIV-1 + anti-CCL2 Ab day 1 p.i.), cluster 6 (HIV-1 + anti-CCL2 Ab day 4 p.i.) and cluster 1 of Figure 2A (anti-CCL2 Ab treatment 4 h). Interestingly, 22 genes were up-regulated in both uninfected and HIV-1–infected MDM cultures, among which the HIV-1 restriction factor coding genes APOBEC3A, EIF2AK2, OAS3, HERC5, IFITM3, ISG15, and RSAD2 (Figure 4F). The functional annotation clustering of the genes up-regulated by CCL2 neutralization at day 4 p.i. also revealed a cluster related to cell division and cytoskeleton organization (cluster 5, enrichment score 15.5, 44 genes), which could be associated to the morphological changes we previously observed in MDMs exposed to anti-CCL2 Ab (14).

### CCL2 Neutralization in HIV-Infected MDMs Down-Regulates Viral Gene Expression

To assess the effect of CCL2 neutralization on the HIV-1 transcriptome, reads that failed to align to hg19 were mapped to the HIV-1BaL genome using Bowtie 1 to quantify viral mRNAs. Cell-associated HIV-1 RNA was detected at both days 1 and 4 p.i., but it was considerably less abundant at the former time p.i. A median of 205 (116–397) and 11,045 (5,090–25,741) reads per sample mapped to HIV-1 at days 1 and 4 p.i., respectively.

None of the existing RNA-seq data analysis packages have reliable tools for precise splice variant measurement from standard RNA-seq datasets (50–100 base pair reads) of complex overlapping sequences as is the case of HIV-1. Thus, due to the difficulties in assigning RNA-seq reads aligned to HIV-1 genome in the overlapping regions of tat and rev, we did not quantify these two transcripts.

**FIGURE 5:**
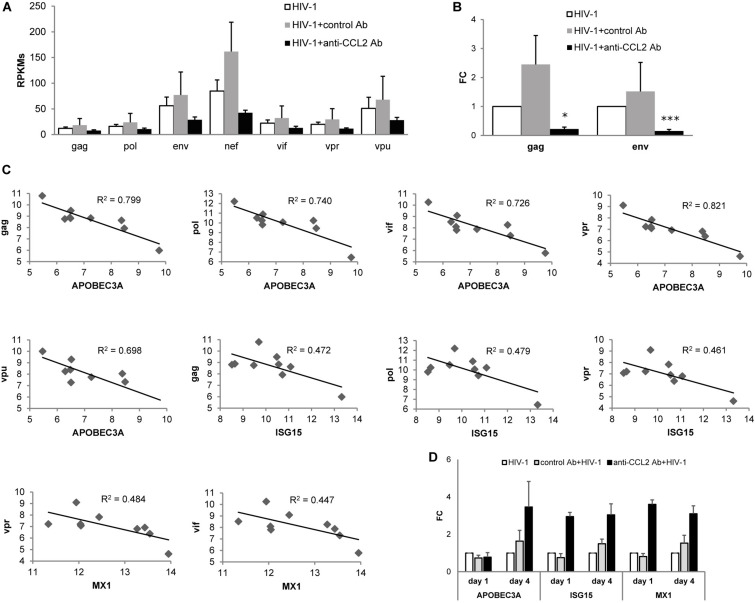
Effect of CCL2 neutralization on the HIV-1 transcriptome. **(A)** RPKM (reads per kilo base per million mapped reads) values (mean values + SE) for HIV-1 transcripts in MDMs treated or not with anti-CCL2 Ab at day 4 p.i. (dataset 3). **(B)** Analysis by qPCR of HIV-1 gag and rev transcripts levels. Data represent mean values (+SE) of the results obtained with three donors. *p < 0.05; ***p < 0.005 (HIV-1 + anti-CCL2 Ab vs. HIV-1 + control Ab). **(C)** Correlation between log2 (DESeq2 normalized counts + 1) for APOBEC3A, ISG15 or MX1 and HIV-1 transcripts in MDMs treated or not with anti-CCL2 or control Ab at day 4 p.i. (dataset 3). **(D)** Analysis by qPCR of the levels of APOBEC3A, ISG15, and MX1 in MDMs treated or not with anti-CCL2 or control Ab at days 1 and 4 p.i. Data represent mean values (+SE) of the results obtained with three donors.

As shown in Figure 5A, treatment with anti-CCL2 Ab determined a strong down-regulation of transcripts coding for structural (env, gag, and pol) as well as regulatory (nef, vif, vpr, and vpu) proteins at day 4 p.i. We thus analyzed the effect of CCL2 neutralization on the expression of gag and env genes by qPCR employing samples from one of the donors used in RNA-seq (dataset 3) and two additional donors. As shown in Figure 5B, this analysis confirmed the downregulation of env and gag transcripts following anti-CCL2 Ab treatment.

We then evaluated whether an association exists between the increase of host defense genes expression and the restriction of viral transcripts levels observed in MDMs following CCL2 blocking. Pearson’s correlation coefficients were calculated to assess the relationship between the level of HIV-1 transcripts in the different conditions and the restriction factors whose expression was increased upon CCL2 neutralization (Figure 4F). A strong inverse correlation was found between APOBEC3A and gag (coefficient = −0.894; *p* = 0.001), pol (coefficient = −0.860; *p* = 0.003), vif (coefficient = −0.852; *p* = 0.004), vpr (coefficient = −0.906; *p* = 0.001), or vpu (coefficient = −0.835; *p* = 0.005), and a moderate inverse correlation was observed between ISG15 and gag (coefficient = −0.687; *p* = 0.041), pol (coefficient = −0.692; *p* = 0.039), or vpr (coefficient = −0.679; *p* = 0.044), and MX1 and vif (coefficient = −0.669; *p* = 0.049) or vpr (coefficient = −0.696; *p* = 0.037) (Figure 5C). A moderate inverse correlation was also found between nef and OAS3 (coefficient = −0.570) or EIF2AK2 (coefficient = −0.418), RSAD2 and vpr (coefficient = −0.522), and HERC and gag (coefficient = −0.596), pol (coefficient = −0.632), vif (coefficient = −0.0.518), vpr (coefficient = −0.589) or vpu (coefficient = −0.542), but they were not statistically significant. As shown in Figure 5D, the differential expression profile of APOBEC3A, ISG15 and MX1 was confirmed by qPCR employing samples from one of the donors used in RNA-seq (dataset 3) and two additional donors.

## Discussion

In this study, we used RNA-seq to perform a deep characterization of the consequences of CCL2 blocking on the macrophage transcriptome. Our results demonstrated a stronger effect of a short (4 h) compared to a long (20 h) time exposure to anti-CCL2 Ab on the MDM gene expression profile. Functional analysis of the early up-regulated genes identified two enriched functionally related gene clusters, which included transcripts coding for several antiviral factors, thus uncovering the induction of a robust innate immune response following CCL2 neutralization. This protective response was reproduced in an independent set of samples, and was still observed at longer time of exposure to anti-CCL2 Ab, in keeping with our previous results (20). Interestingly, we described an NF-κB–miR-155–positive regulatory network potentially underlying the modulation of the immune response by CCL2 blocking. MiR-155 is a multifunctional miRNA enriched in cells of the immune system and essential for the immune response. In innate immune cells such as monocytes, macrophages, and dendritic cells, it can play either positive or negative roles in the control of the inflammatory response, which likely depend on cell type–associated differences in the pool of miR-155 direct/indirect targets being expressed and subjected to repression (34). Of note, miR−155 has been found to suppress HIV-1 infection in activated macrophages by targeting the host-dependency factors ADAM10, TNPO3, Nup153, and LEDGF/p75 (35). However, these genes were not differentially expressed in the RNA-seq datasets herein reported. Thus, based on the excellent correlation we found between RNA-seq and qPCR data, we believe these factors are unlikely to contribute to HIV-1 resistance in our experimental model. The miR-155 gene contains binding sites for multiple TFs, including NF-κB and IRFs (36). Our results indeed demonstrated that miR-155 up-regulation following CCL2 neutralization was mediated by NF-κB. Furthermore, one miR-155 target gene identified among those down-regulated upon anti-CCL2 Ab treatment, was c-Fos, a TF that can repress NFKB1 transcription and inhibit NF-κB activation (37). A positive correlation between miR-155 up-regulation and NF-κB activation has been described in macrophages as well as in other cell types (33, 38–40). In macrophages, miR-155 function was found to be counterbalanced by miR-146, thus suggesting that the two miRNAs and NF-κB signaling form a complex network of cross-regulations, which control gene transcription and modulate inflammatory responses (33).

The results herein reported demonstrated that activation of innate immune pathways following CCL2 neutralization also occurred in HIV-1–infected macrophage cultures. The virus itself had a very limited effect on MDM gene expression and did not induce a type I IFN signature, which has otherwise been observed upon CCL2 blocking. Previous studies addressing the consequences of HIV-1 infection on the macrophage transcriptome reported either differential expression of several genes (41–46) or small changes to the host cell transcriptome (9, 47). In addition, stimulation of ISGs expression by HIV-1 infection was observed in some studies (41, 43, 46, 47) but not in others (9, 44, 45). The lack of induction of innate immune responses was also detected in cell lines of different origin following single-cycle HIV-1 infection (10), and the expression of some antiviral genes in macrophages was even found to be down-regulated upon HIV-1 infection (48). Most of these studies were carried out using different types of microarrays, with the only exception of the work by Deshiere et al., which employed an RNA-seq approach. Several possible technical issues may explain inconsistency among results. In particular, the features of viral stocks used to infect macrophages may have influenced the outcomes. Here, we have utilized pelleted virus to minimize the risk of contamination with soluble factors, such as cytokines, which are released from the host cells used to produce the virus and that may elicit confounding immunological responses. Purified virus was likewise employed by Tsang et al., which reported very small changes in the MDM transcriptome at days 1 and 7 p.i. without evidence of innate immune cellular activation. Another key aspect concerns the parameters used for DEGs quantitation, which greatly influence analysis outcomes. Because most previous studies based this measure on *p* value, the omission of a more restrictive FDR estimate may have overrated the number of DEGs in HIV-1–infected cells. Variability in gene expression among donors may have also impacted the outcomes. Indeed, in some studies, differences in gene expression were only detected using paired sample analysis (i.e., intradonor) (44, 49). In our study, the high donor variability in dataset 3 might have contributed at least in part to underestimate the effect of HIV-1 infection on the MDM transcriptome. This heterogeneity in the macrophage response to HIV-1 may reflect differences in susceptibility to infection and/or level of constitutive activation of individual donor cells. Finally, the kinetic of samplings may have also influenced the outcome of viral infection on host gene transcription. In this regard, a stronger effect of HIV-1 infection was reported at early times p.i. (i.e., <24 h), whereas a reduced transcriptional response was observed at later time points (i.e., 3–5 days p.i.) in some studies (42, 44). Here, the time points to sample were set based on our previous observation that CCL2 neutralization strongly influenced the kinetics of HIV-1 DNA accumulation in MDMs. In particular, while a marked increase of viral DNA was observed in both untreated and control Ab–treated cells at 7 vs. 4 days p.i., similar levels of HIV-1 DNA were detected at these two time points in anti-CCL2 Ab–treated MDMs (20). These results suggested that the molecular pathways underlying viral restriction were likely triggered at very early time points after infection, and we thus performed RNA-seq at days 1 and 4 p.i. A presumably very low proportion of infected MDMs at these early time points p.i. likely contributed at least in part to the limited effect of HIV-1 on the macrophage transcriptome. Although we show here that the innate response elicited by CCL2 neutralization was stronger at 1 compared to 4 days p.i., a different expression of some antiviral factors may explain the observed kinetic of viral restriction.

Several DEGs may act as restriction factors, thus playing a role in HIV-1 replication inhibition in anti-CCL2 Ab–treated MDMs. Restriction factors are structurally and functionally diverse cellular proteins that are part of the innate response and may target HIV-1 replication at essentially each step of the replication cycle (50, 51). Interestingly, the expression of some of these genes inversely correlated with viral transcripts levels. The most significant correlation was found for APOBEC3A. This enzyme is a myeloid specific member of the APOBEC3 family of cytidine deaminases playing crucial roles in antiviral innate immunity. In monocytes, APOBEC3A, together with APOBEC3G, represents a potent innate barrier to HIV-1 infection, which diminishes during differentiation to macrophages, resulting in a more susceptible population of target cells (52). APOBEC3G is encapsidated into virions and blocks viral replication upon entry in newly infected cells primarily by causing C-to-U deamination on the single stranded viral DNA during reverse transcription, leading to either the degradation of viral DNA by cellular repair mechanisms or the hypermutation of the viral genome. Conversely, APOBEC3A can restrict infection directly in the target cells where it is endogenously expressed, and it was indeed identified as a specific inhibitor of the early phases of HIV-1 infection in macrophages (53). The results herein reported strengthen our view that CCL2/CCR2 blocking regulates the expression of this innate intracellular viral antagonist. In fact, our previous studies demonstrated an increase of APOBEC3A expression in uninfected (after 20-h exposure to anti-CCL2 Ab) and HIV-1–infected (14 days p.i.) MDMs upon CCL2 neutralization, as well as in PBMCs of HIV-1–infected subjects treated with the CCR5/CCR2 inhibitor cenicriviroc. This effect was associated with reduced levels of viral DNA accumulation in macrophages and of the inflammatory marker soluble CD14 in infected individuals (20, 54). It is worth noting that APOBEC3A restricts HIV-1 replication acting at postentry steps of the virus life cycle, which are indeed those demonstrated to be affected by CCL2 blocking. Although correlation does not necessarily indicate causation, these results support the hypothesis that enhanced expression of at least some of these antiviral factors may provide protective effects limiting HIV-1 replication in macrophages.

IFNs are main inducers of APOBEC3 expression (55). Our previous work suggested that CCL2 neutralization-mediated increase of APOBEC3A expression in MDMs was type I IFN independent (20, 56). In the RNA-seq datasets herein reported, we did not observe IFN transcripts upregulation upon CCL2 blocking. However, we cannot exclude that low IFN induction could be detected in MDMs at very early times of anti-CCL2 Ab treatment (<4 h). A STAT1-regulated IFN-like response in the absence of IFN expression was reported in fibroblasts derived from Hutchinson–Gilford progeria syndrome patients (57). Non-canonical IFN-independent activation of STAT1 was reported by other groups (58, 59), although it can be unrelated to ISG induction (58). Similarly, IRF1 was shown to be able to maintain antiviral genes expression and regulate antiviral responses in the absence of IFNs (60, 61). Interestingly, we found STAT1 and IRF1 signatures in the genes up-modulated by CCL2 neutralization (Table 2). Clarifying whether IFN-dependent or independent pathways or both contribute to the activation of innate immune responses elicited upon CCL2 neutralization will require additional work.

HIV-1 can circumvent host antiviral signaling and establish persistent viral reservoirs by blocking antiviral responses in several ways, which include the impairment of receptors involved in pathogen detection, downstream signaling cascades required for IFN up-regulation, and expression or function of key antiviral proteins (62). Our previous results demonstrated that HIV-1 infection leads to increased CCL2 expression (14, 20), and here we report that CCL2 blocking strengthens the host immune response. Enhancing antiviral responses while controlling immune cell activation is an attractive strategy to control HIV-1 replication. The results herein reported suggest that the protective effect of CCL2 neutralization may be determined by a balance between proinflammatory and antiviral responses. Actually, NF-κB–mediated inflammation may lead to the activation of HIV-1 target cells, but CCL2 blocking boosts innate antiviral responses, thus restricting HIV-1 replication. In addition, these results improve our understanding on how the innate immune response can be blocked in macrophages, demonstrate that these defects can be reversed, and are therefore of great importance to develop novel therapeutic strategies aimed at eradicating the HIV-1 reservoir.

## Data Availability Statement

The datasets presented in this study can be found in online repositories. The names of the repository/repositories and accession number(s) can be found at: https://www.ebi.ac.uk/arrayexpress/, E-MTAB-9414.

## Ethics Statement

Healthy donor Buffy coats were obtained from Centro Trasfusionale–Sapienza University of Rome not specifically for this study. Informed consent was not asked because data were analyzed anonymously. Data from healthy donors were treated by Centro Trasfusionale according to the Italian law on personal data management “Codice in Materia di Protezione dei dati Personali” (Testo unico D.L. June 30, 2003 n. 196).

## Author Contributions

DAC, MVC, and GF designed and performed the experiments, analyzed the results, and edited the manuscript. KEK-U, JL, and MFV analyzed the data and designed the figures. LC, CP, CMG, RA, and MA performed the experiments. MCG and MP assisted in the experimental design and data interpretation and edited the manuscript. LF conceived the experiments, analyzed the data, supervised the project, and wrote and edited the manuscript. All authors reviewed and approved the final manuscript.

## Conflict of Interest

The authors declare that the research was conducted in the absence of any commercial or financial relationships that could be construed as a potential conflict of interest.

## Footnotes

1 https://cran.r-project.org/web/packages/pheatmap

2 https://biit.cs.ut.ee/clustvis/

3 https://www.rdocumentation.org/packages/graphics/versions/3.6.2/topics/boxplot

4 https://www.rdocumentation.org/packages/EnhancedVolcano/versions/1.5.4

5 http://bioinfogp.cnb.csic.es/tools/venny/index.html

6 https://cytoscape.org/

## Abbreviations

Ab: antibody
APOBEC3: apolipoprotein B mRNA editing enzyme catalytic polypeptide 3
cART: combined antiretroviral therapy
CCL2: CC chemokine ligand 2
CCR2: CC chemokine receptor 2
DAVID, database for Annotation: Visualization and Integrated Discovery
DEG: differentially expressed gene
EIF2AK2, interferon-induced: double-stranded RNA-activated protein kinase 2
FDR: false discovery rate
HERC5: HECT and RLD domain containing E3 ubiquitin protein ligase 5
HIV-1: human immunodeficiency virus type 1
IFITM3: interferon induced transmembrane protein 3
ISG: interferon-stimulated gene
MDM: monocyte-derived macrophage
miR: microRNA
NF- κ B: nuclear factor kappa B
NFKB1: nuclear factor kappa B subunit 1
OAS: 2′-5′-oligoadenylate Synthetase
PCA: principal component analysis
qPCR: quantitative RT-PCR
RELA: v-rel avian reticuloendotheliosis viral oncogene homolog A
RNA-seq: RNA sequencing
RSAD2: radical S-adenosyl methionine domain containing 2
SAMHD1: SAM and HD domain containing deoxynucleoside triphosphate triphosphohydrolase 1
SEM: structural equation model
TCID_50_: tissue culture infectious dose 50
TF: transcription factor
TRRUST: transcriptional Regulatory Relationships Unraveled by Sentence-based Text mining.
