# Supplementary material for: Transcriptome Profiling of Human Monocyte-Derived Macrophages Upon CCL2 Neutralization Reveals an Association Between Activation of Innate Immune Pathways and Restriction of HIV-1 Gene Expression
Source: Front Immunol. 2020 Sep 18;11:2129. doi: 10.3389/fimmu.2020.02129 (PMC7531389; doi:10.3389/fimmu.2020.02129)
Supplement: Supplementary file 1 [file Data_Sheet_1.zip › Supplementary tables/Covino et al_Supplementary Table 1.pdf]

Supplementary Table 1. List of up-regulated genes in MDMs exposed to anti-CCL2 Ab for 4 hours.

gene	*log2FC*	*pvalue*	*padj*	*FC*
MMP10	11.84	9.35E-06	1.28E-04	3668.58
IRG1	7.58	3.09E-09	9.42E-08	190.71
ADGRG7	7.11	4.42E-03	2.30E-02	138.14
TNIP3	6.87	4.32E-07	8.03E-06	116.97
IL36G	6.48	1.21E-07	2.52E-06	89.44
NDP	6.29	8.57E-18	1.18E-15	78.07
XIRP1	6.10	7.94E-33	3.68E-30	68.37
SERPINB2	5.96	6.79E-12	3.58E-10	62.28
CCL20	5.75	9.70E-20	1.54E-17	53.66
GFPT2	5.72	2.06E-06	3.28E-05	52.57
NDRG4	5.58	3.22E-14	2.43E-12	47.90
INHBA	5.55	1.66E-20	2.93E-18	47.00
OLIG2	5.46	5.66E-13	3.55E-11	44.03
MMP1	5.29	1.11E-12	6.58E-11	39.01
IL1B	5.23	1.87E-06	3.02E-05	37.52
IL1A	5.15	1.56E-06	2.56E-05	35.46
VGF	5.07	4.05E-04	3.25E-03	33.63
NIPAL1	4.97	4.77E-06	7.00E-05	31.34
ANKRD1	4.89	2.13E-03	1.29E-02	29.55
NT5E	4.73	2.96E-14	2.27E-12	26.49
TRIM9	4.65	7.12E-13	4.39E-11	25.19
FAM19A3	4.65	1.21E-06	2.03E-05	25.04
IL36RN	4.63	1.04E-34	5.92E-32	24.74
TNC	4.63	1.59E-23	4.15E-21	24.72
TM4SF1	4.62	1.27E-11	6.29E-10	24.60
IL7R	4.58	3.41E-36	2.25E-33	23.97
TRAF1	4.57	1.46E-50	6.12E-47	23.78
SSTR2	4.56	4.85E-34	2.43E-31	23.66
LRRC32	4.55	9.34E-47	1.95E-43	23.42
EREG	4.46	4.86E-59	6.09E-55	22.06
RRAD	4.46	9.21E-12	4.75E-10	21.99
COL1A1	4.41	2.83E-06	4.39E-05	21.21

SLAMF1	4.39	1.27E-06	2.13E-05	20.93
HS3ST3B1	4.38	3.19E-07	6.13E-06	20.88
C1QTNF1	4.37	2.52E-04	2.20E-03	20.65
ELOVL7	4.36	1.84E-49	5.77E-46	20.49
LAD1	4.31	8.39E-14	6.04E-12	19.78
HEY1	4.29	5.15E-17	5.93E-15	19.57
CXCL11	4.24	1.89E-09	5.98E-08	18.90
IDO1	4.22	1.78E-07	3.62E-06	18.58
CCL4	4.21	1.41E-36	9.80E-34	18.45
IL1RN	4.19	2.05E-39	1.79E-36	18.29
SERPINE2	4.19	5.51E-07	1.00E-05	18.27
APCDD1L	4.14	5.81E-14	4.24E-12	17.67
TNFSF18	4.09	5.72E-06	8.24E-05	17.00
G0S2	4.04	1.13E-05	1.51E-04	16.50
EDN1	4.04	2.26E-20	3.94E-18	16.41
EGLN3	4.03	2.94E-14	2.27E-12	16.30
CXCL5	3.97	9.51E-26	2.91E-23	15.71
LOC440896	3.97	8.36E-04	5.95E-03	15.65
S1PR3	3.96	1.31E-08	3.45E-07	15.55
TNFAIP6	3.96	1.07E-06	1.83E-05	15.55
FERMT2	3.95	1.18E-53	7.38E-50	15.48
ETV7	3.95	4.31E-08	9.92E-07	15.44
PTX3	3.90	6.30E-20	1.03E-17	14.89
COL5A3	3.90	1.19E-09	3.99E-08	14.88
IL2RA	3.89	4.17E-04	3.33E-03	14.87
CCL24	3.86	2.01E-28	7.64E-26	14.55
LOC100128993	3.81	2.08E-07	4.14E-06	14.07
SLCO4A1	3.80	1.87E-22	4.20E-20	13.96
AK4	3.79	3.70E-46	6.62E-43	13.83
CSF2	3.77	3.69E-07	6.99E-06	13.61
NIPAL4	3.75	6.07E-22	1.25E-19	13.43
CCL8	3.74	7.60E-05	7.94E-04	13.38
CYP7B1	3.74	6.40E-04	4.74E-03	13.38
CXCL10	3.73	2.22E-05	2.74E-04	13.30
LRTM2	3.73	1.63E-04	1.53E-03	13.26
PHF24	3.73	1.94E-06	3.12E-05	13.25

SOCS3	3.72	4.96E-05	5.55E-04	13.14
RSAD2	3.70	3.71E-06	5.59E-05	13.03
ANGPTL4	3.70	5.59E-17	6.31E-15	13.00
ITGB8	3.70	2.59E-05	3.12E-04	12.99
ABTB2	3.69	1.08E-04	1.07E-03	12.94
DUSP5	3.65	2.35E-20	3.99E-18	12.54
PLOD2	3.63	1.36E-04	1.31E-03	12.40
BAALC-AS2	3.63	9.47E-12	4.84E-10	12.40
IL36B	3.61	1.06E-05	1.43E-04	12.24
GBP5	3.61	3.85E-15	3.35E-13	12.21
GCNT4	3.60	3.71E-05	4.26E-04	12.16
ZSWIM5	3.57	2.76E-05	3.28E-04	11.90
GPRC5A	3.57	3.15E-13	2.06E-11	11.84
SOCS1	3.55	1.15E-03	7.71E-03	11.74
SH2D4A	3.53	1.10E-04	1.08E-03	11.57
RND3	3.52	1.61E-43	2.24E-40	11.45
CXCL8	3.52	1.42E-17	1.84E-15	11.44
CKB	3.50	5.63E-14	4.13E-12	11.31
OASL	3.47	4.43E-15	3.80E-13	11.07
KANK1	3.45	1.17E-40	1.33E-37	10.91
SRSF12	3.44	3.38E-05	3.92E-04	10.88
N4BP3	3.42	1.46E-09	4.75E-08	10.70
DYRK3	3.36	2.34E-11	1.10E-09	10.25
OLFM2	3.31	1.46E-23	3.88E-21	9.95
C17orf96	3.28	2.42E-17	2.91E-15	9.70
MMP19	3.24	2.19E-24	6.25E-22	9.46
MYEOV	3.24	2.11E-08	5.24E-07	9.45
CCL7	3.24	2.02E-35	1.20E-32	9.44
CILP2	3.24	4.72E-09	1.37E-07	9.44
EHD1	3.23	8.10E-19	1.19E-16	9.41
SERPINE1	3.23	1.32E-18	1.91E-16	9.38
CD274	3.23	9.23E-05	9.40E-04	9.36
DNAAF1	3.21	1.60E-04	1.50E-03	9.27
LAMA5	3.19	1.61E-17	2.06E-15	9.14
MIR155HG	3.19	2.31E-17	2.81E-15	9.10
FOSL1	3.18	7.01E-22	1.42E-19	9.03

LOC731424	3.17	6.51E-04	4.81E-03	9.01
CEMIP	3.15	2.44E-07	4.78E-06	8.87
F3	3.14	9.89E-23	2.30E-20	8.84
GBP1	3.14	7.71E-15	6.48E-13	8.83
IFIT3	3.13	3.63E-04	2.97E-03	8.76
MGC12916	3.13	1.32E-11	6.49E-10	8.75
CHST2	3.12	3.51E-14	2.62E-12	8.72
EHD2	3.12	6.35E-11	2.71E-09	8.68
PTGS2	3.12	3.98E-03	2.11E-02	8.68
CSF3	3.10	8.32E-03	3.83E-02	8.56
IRAK2	3.09	1.18E-28	4.77E-26	8.50
ZNF462	3.07	5.44E-10	1.97E-08	8.39
ACKR4	3.05	1.77E-03	1.10E-02	8.31
MET	3.05	1.27E-14	1.03E-12	8.31
C6orf223	3.04	8.39E-07	1.47E-05	8.25
HCAR3	3.03	5.86E-08	1.31E-06	8.18
SLC2A3	3.03	1.63E-39	1.57E-36	8.15
UPB1	3.00	4.90E-06	7.17E-05	8.03
SEC14L2	2.99	4.14E-13	2.65E-11	7.94
CD80	2.96	3.56E-09	1.07E-07	7.76
GBP2	2.96	1.14E-31	5.10E-29	7.76
PTRF	2.95	1.28E-10	5.20E-09	7.71
MFSD2A	2.94	1.83E-17	2.26E-15	7.70
CCND1	2.91	3.68E-23	9.42E-21	7.50
IFIT2	2.89	3.16E-03	1.77E-02	7.42
TMEM217	2.89	1.00E-11	5.06E-10	7.40
PTP4A3	2.89	7.92E-05	8.21E-04	7.39
CCL2	2.88	9.81E-12	4.98E-10	7.34
EBF1	2.87	6.81E-04	5.01E-03	7.32
TLE1	2.87	3.61E-16	3.71E-14	7.32
DUSP4	2.86	7.86E-09	2.17E-07	7.28
SYPL2	2.86	5.09E-07	9.36E-06	7.24
MSC	2.85	1.38E-40	1.44E-37	7.22
NR4A3	2.84	2.58E-34	1.35E-31	7.17
LIF	2.84	4.42E-12	2.42E-10	7.15
JAG1	2.81	7.20E-13	4.42E-11	7.04

PRR16	2.81	2.04E-07	4.07E-06	7.02
NXN	2.81	4.68E-05	5.26E-04	7.01
WTAP	2.80	9.83E-23	2.30E-20	6.96
HOMER1	2.79	9.40E-13	5.65E-11	6.93
CCL3	2.78	2.35E-14	1.85E-12	6.89
SLC39A8	2.78	2.89E-24	8.05E-22	6.85
PBX4	2.76	1.98E-10	7.70E-09	6.80
HAPLN3	2.76	6.96E-06	9.75E-05	6.79
SGPP2	2.76	1.70E-13	1.17E-11	6.78
ELFN1	2.76	4.74E-07	8.75E-06	6.76
CAV1	2.75	2.34E-04	2.08E-03	6.71
ADM	2.74	6.70E-16	6.67E-14	6.70
PHLDB1	2.74	1.32E-47	3.30E-44	6.67
RASGRP1	2.73	3.31E-08	7.77E-07	6.61
DPYSL4	2.70	9.17E-04	6.40E-03	6.49
FCAR	2.69	1.75E-35	1.10E-32	6.46
BIRC3	2.69	5.39E-11	2.33E-09	6.45
GCH1	2.68	4.55E-04	3.55E-03	6.41
MYO1B	2.68	3.13E-11	1.42E-09	6.39
FEZ1	2.66	5.67E-04	4.28E-03	6.33
DUSP6	2.64	3.92E-43	4.91E-40	6.25
SLC2A14	2.63	2.26E-03	1.34E-02	6.21
CXCL1	2.63	2.79E-03	1.60E-02	6.20
INSIG1	2.63	5.74E-46	8.99E-43	6.18
EBI3	2.62	1.48E-08	3.85E-07	6.14
MAMLD1	2.62	2.64E-22	5.61E-20	6.13
HMGN2P46	2.60	5.45E-03	2.71E-02	6.08
IL27	2.60	7.34E-05	7.71E-04	6.06
EGR3	2.58	2.48E-14	1.94E-12	5.98
ATP2B1	2.58	1.20E-24	3.51E-22	5.97
PDE4B	2.57	6.64E-11	2.81E-09	5.95
ATP8B2	2.57	1.48E-15	1.39E-13	5.95
CNKSR3	2.54	5.87E-07	1.06E-05	5.80
PDGFA	2.54	2.51E-34	1.35E-31	5.80
LINC00346	2.53	2.06E-07	4.11E-06	5.79
ICAM1	2.53	4.52E-12	2.46E-10	5.76

SPINK1	2.50	2.18E-03	1.31E-02	5.67
CMPK2	2.49	1.64E-11	7.95E-10	5.63
LPAR3	2.49	1.10E-03	7.41E-03	5.60
TLR2	2.48	2.40E-22	5.19E-20	5.57
STON2	2.48	1.37E-08	3.58E-07	5.56
PMAIP1	2.47	4.58E-11	2.01E-09	5.53
PDCD1	2.47	1.25E-08	3.31E-07	5.53
TMEM88	2.46	2.82E-03	1.61E-02	5.50
B4GALT5	2.46	4.43E-25	1.32E-22	5.49
USP18	2.43	5.37E-03	2.68E-02	5.38
CSF1	2.43	9.10E-21	1.68E-18	5.38
BCL2A1	2.43	2.76E-22	5.76E-20	5.37
TMEM132A	2.42	2.85E-07	5.49E-06	5.36
ACSL4	2.42	2.45E-38	1.92E-35	5.35
CCL5	2.42	4.38E-10	1.63E-08	5.33
DOT1L	2.40	1.66E-26	5.48E-24	5.29
ANKRD33B	2.39	1.83E-05	2.31E-04	5.26
CD83	2.39	8.00E-28	2.95E-25	5.26
RNF144B	2.39	1.25E-12	7.36E-11	5.26
TNF	2.39	1.43E-09	4.68E-08	5.25
ETV3L	2.39	2.11E-05	2.61E-04	5.25
ICAM5	2.39	2.72E-08	6.55E-07	5.25
ITGB3	2.38	6.49E-13	4.04E-11	5.21
ARRDC4	2.35	5.69E-18	8.01E-16	5.11
IFI44L	2.35	3.21E-03	1.78E-02	5.09
ISG20	2.35	6.05E-10	2.15E-08	5.09
BAALC	2.35	5.74E-10	2.06E-08	5.09
GJB2	2.34	9.77E-03	4.35E-02	5.06
SLC7A5	2.34	4.66E-12	2.51E-10	5.05
TNFRSF4	2.34	1.80E-14	1.43E-12	5.05
ISG15	2.33	1.53E-15	1.43E-13	5.04
ESPL1	2.32	7.92E-22	1.57E-19	5.00
ARHGAP23	2.32	1.10E-12	6.54E-11	4.99
CYP27B1	2.31	1.68E-12	9.66E-11	4.97
SMARCA1	2.31	1.85E-05	2.33E-04	4.97
TBX21	2.31	5.44E-06	7.90E-05	4.95

TNFRSF9	2.30	5.68E-10	2.05E-08	4.91
RNF175	2.29	1.00E-09	3.45E-08	4.87
SLC7A11	2.28	4.11E-16	4.19E-14	4.86
LRP12	2.28	1.34E-13	9.32E-12	4.86
C15orf48	2.28	1.31E-33	6.33E-31	4.85
USP12	2.28	2.46E-13	1.65E-11	4.85
SLC2A1	2.28	5.74E-16	5.76E-14	4.84
HIVEP2	2.27	1.29E-09	4.26E-08	4.83
KIAA1644	2.27	2.85E-04	2.44E-03	4.82
LINC00622	2.26	5.04E-04	3.87E-03	4.78
SOD2	2.25	1.35E-11	6.60E-10	4.77
FHL2	2.25	8.70E-04	6.16E-03	4.75
CASP5	2.25	1.47E-03	9.48E-03	4.74
TNFSF14	2.24	1.40E-07	2.89E-06	4.73
HILPDA	2.23	5.08E-11	2.21E-09	4.70
BATF2	2.23	3.14E-13	2.06E-11	4.69
ETS2	2.23	2.56E-08	6.21E-07	4.68
CCL4L2	2.22	3.53E-03	1.93E-02	4.67
LINC01215	2.22	1.41E-04	1.35E-03	4.67
CDC42EP2	2.22	7.73E-07	1.36E-05	4.67
DDX58	2.22	4.59E-03	2.37E-02	4.67
GEM	2.22	7.06E-19	1.05E-16	4.66
MPP6	2.22	3.72E-12	2.04E-10	4.65
CXCL9	2.21	1.75E-04	1.62E-03	4.64
TNIP1	2.21	1.67E-14	1.34E-12	4.62
SRRM3	2.21	2.28E-03	1.35E-02	4.62
CAV2	2.20	4.18E-04	3.34E-03	4.61
MAOA	2.18	1.05E-15	1.01E-13	4.53
NEURL3	2.18	1.07E-04	1.06E-03	4.52
PLAUR	2.17	8.67E-20	1.39E-17	4.50
MDGA1	2.17	7.11E-04	5.18E-03	4.50
AQP9	2.16	1.22E-14	9.97E-13	4.46
AKR1C1	2.15	1.03E-02	4.51E-02	4.44
GBP4	2.15	2.85E-06	4.42E-05	4.44
SLC41A2	2.14	7.78E-18	1.08E-15	4.42
HIC1	2.14	5.53E-07	1.00E-05	4.40

ZNF697	2.14	1.08E-17	1.46E-15	4.40
SMOX	2.14	1.42E-19	2.20E-17	4.39
TNFAIP3	2.13	2.29E-11	1.07E-09	4.39
BMP6	2.13	7.37E-04	5.36E-03	4.37
NOCT	2.13	1.64E-05	2.10E-04	4.36
HELZ2	2.12	2.60E-07	5.03E-06	4.36
PDPN	2.12	2.47E-07	4.81E-06	4.36
RHCG	2.12	1.08E-02	4.70E-02	4.34
LRRC16B	2.10	6.50E-03	3.11E-02	4.29
GPC1	2.10	1.38E-10	5.52E-09	4.28
MSANTD3	2.09	8.84E-24	2.41E-21	4.26
MX1	2.09	2.77E-02	9.58E-02	4.26
PNP	2.08	2.53E-13	1.69E-11	4.22
CLEC4E	2.07	1.20E-10	4.92E-09	4.21
TNFAIP8	2.07	3.62E-10	1.35E-08	4.21
NTN1	2.07	1.03E-03	7.06E-03	4.20
FSCN1	2.07	9.53E-08	2.06E-06	4.20
NFKB1	2.06	4.43E-13	2.82E-11	4.16
IL1R2	2.05	3.93E-07	7.39E-06	4.15
AFAP1	2.05	8.85E-03	4.01E-02	4.14
GLIS3	2.05	9.73E-09	2.66E-07	4.13
PTGER2	2.05	4.74E-12	2.54E-10	4.13
PTPRE	2.04	1.98E-30	8.55E-28	4.12
C1orf21	2.04	3.76E-06	5.65E-05	4.11
JARID2	2.03	2.57E-17	3.06E-15	4.09
IL7	2.03	8.10E-03	3.75E-02	4.08
DNAJB5	2.02	2.35E-20	3.99E-18	4.06
PDK1	2.01	3.56E-19	5.44E-17	4.04
IRF1	2.01	5.27E-10	1.91E-08	4.04
CSPG4	2.01	3.35E-06	5.08E-05	4.03
MB21D2	2.01	1.26E-10	5.16E-09	4.03
LOC100289511	2.01	8.34E-10	2.90E-08	4.02
ECE1	2.00	1.24E-19	1.94E-17	3.99
NFKBID	1.99	3.97E-13	2.55E-11	3.98
ACHE	1.99	9.40E-05	9.53E-04	3.98
STAT4	1.98	1.44E-05	1.87E-04	3.95

KLF9	1.98	3.67E-15	3.22E-13	3.94
PDSS1	1.98	1.37E-06	2.29E-05	3.94
IER3	1.97	1.79E-08	4.55E-07	3.93
DUSP8	1.97	3.20E-03	1.78E-02	3.92
SPAG5	1.97	6.11E-09	1.73E-07	3.91
CLEC4D	1.97	2.48E-04	2.17E-03	3.91
MTF1	1.97	4.76E-13	3.01E-11	3.90
MX2	1.96	8.16E-03	3.77E-02	3.89
FSD1L	1.96	9.61E-06	1.31E-04	3.88
ATF3	1.95	1.30E-20	2.36E-18	3.86
NAMPT	1.94	1.99E-02	7.42E-02	3.84
STX1A	1.93	1.77E-09	5.70E-08	3.82
UBTD2	1.93	1.28E-17	1.68E-15	3.81
ETNK2	1.93	1.62E-02	6.37E-02	3.80
AQP3	1.92	3.72E-09	1.11E-07	3.77
BTG1	1.91	1.30E-26	4.42E-24	3.77
CTHRC1	1.91	2.81E-03	1.60E-02	3.75
SGMS2	1.91	2.25E-22	4.94E-20	3.75
GRAMD1A	1.91	2.27E-02	8.21E-02	3.75
MIR210HG	1.90	1.09E-07	2.32E-06	3.74
BTG3	1.90	4.60E-12	2.49E-10	3.72
KIAA0226L	1.90	2.80E-16	2.95E-14	3.72
MMP12	1.89	3.76E-07	7.11E-06	3.72
PHLDA1	1.89	8.35E-15	6.93E-13	3.70
C7orf60	1.89	6.68E-12	3.53E-10	3.70
RTN4RL2	1.88	1.72E-06	2.83E-05	3.69
SDC4	1.88	7.64E-17	8.55E-15	3.68
PHLDA2	1.88	1.86E-05	2.34E-04	3.68
ST3GAL5-AS1	1.88	1.03E-02	4.50E-02	3.67
LOC143666	1.87	6.07E-07	1.09E-05	3.66
ARC	1.87	1.76E-02	6.78E-02	3.66
RHBDL3	1.87	4.58E-03	2.36E-02	3.65
MAP2K3	1.86	2.81E-21	5.41E-19	3.64
SLAMF7	1.86	1.94E-07	3.91E-06	3.64
ZC3H12A	1.86	1.87E-06	3.02E-05	3.63
KMO	1.84	4.76E-17	5.58E-15	3.57

SLC39A14	1.83	1.09E-05	1.46E-04	3.56
BATF3	1.83	5.28E-04	4.03E-03	3.56
RASSF5	1.83	2.09E-15	1.89E-13	3.56
RGS16	1.83	3.42E-07	6.54E-06	3.55
MYO10	1.83	2.15E-12	1.22E-10	3.55
TSC22D1	1.82	3.65E-11	1.63E-09	3.54
ENO2	1.81	2.62E-09	8.05E-08	3.50
IGLON5	1.80	7.31E-05	7.69E-04	3.49
ADAM19	1.80	7.12E-03	3.36E-02	3.49
RGCC	1.80	4.57E-07	8.46E-06	3.49
CRIM1	1.80	5.27E-07	9.65E-06	3.48
DDX60L	1.80	9.40E-13	5.65E-11	3.48
OTUD4	1.80	3.48E-12	1.93E-10	3.48
ABL2	1.79	3.36E-15	2.97E-13	3.47
MIR4435-2HG	1.79	2.30E-05	2.81E-04	3.46
HIVEP3	1.79	1.16E-16	1.27E-14	3.46
VEGFA	1.79	3.06E-14	2.34E-12	3.46
OTUD7B	1.79	2.37E-06	3.73E-05	3.45
DDX21	1.79	7.32E-13	4.47E-11	3.45
KCNJ2	1.78	2.12E-05	2.62E-04	3.44
LOC100126784	1.78	1.29E-02	5.37E-02	3.43
SHANK1	1.78	1.53E-02	6.09E-02	3.43
LIMS2	1.77	2.29E-05	2.80E-04	3.41
METTL1	1.77	3.03E-16	3.16E-14	3.41
CD44	1.77	4.62E-12	2.49E-10	3.41
TNFRSF18	1.77	1.84E-09	5.85E-08	3.40
LINC00152	1.76	1.02E-07	2.18E-06	3.39
BAHCC1	1.76	1.61E-08	4.13E-07	3.39
C19orf26	1.76	3.93E-03	2.10E-02	3.39
MAFF	1.76	1.94E-11	9.18E-10	3.39
ITPRIP	1.76	1.05E-09	3.60E-08	3.38
ZSWIM4	1.75	3.37E-08	7.88E-07	3.37
IFITM1	1.75	1.15E-04	1.13E-03	3.37
HES4	1.75	9.83E-04	6.78E-03	3.37
PIM1	1.74	1.44E-05	1.87E-04	3.35
SNHG15	1.74	1.12E-08	3.04E-07	3.34

EHF	1.74	2.71E-02	9.43E-02	3.34
NFKBIA	1.73	1.64E-09	5.32E-08	3.32
TNIP2	1.73	4.54E-15	3.87E-13	3.31
DDIT4	1.73	1.67E-10	6.54E-09	3.31
TMEM2	1.72	4.34E-15	3.75E-13	3.30
KCNN4	1.72	1.60E-15	1.49E-13	3.29
MAGI2-AS3	1.72	1.13E-07	2.39E-06	3.28
ATP13A3	1.71	3.64E-18	5.19E-16	3.28
DMWD	1.71	4.72E-04	3.65E-03	3.28
MYC	1.71	3.57E-12	1.97E-10	3.27
IFITM3	1.71	1.93E-05	2.42E-04	3.27
HBEGF	1.71	3.10E-08	7.34E-07	3.26
POU2F2	1.70	1.15E-07	2.42E-06	3.26
DRAM1	1.70	5.41E-08	1.22E-06	3.24
CLCF1	1.70	1.17E-07	2.46E-06	3.24
DCHS1	1.69	1.43E-02	5.80E-02	3.23
CHST15	1.69	4.07E-07	7.63E-06	3.23
TJP1	1.69	9.91E-05	9.97E-04	3.23
OLR1	1.69	1.37E-10	5.50E-09	3.22
CSRNP1	1.69	6.40E-05	6.84E-04	3.22
REL	1.68	7.98E-05	8.26E-04	3.22
PDGFRB	1.68	1.15E-02	4.90E-02	3.19
SPSB1	1.67	1.02E-15	9.82E-14	3.19
TNFRSF12A	1.67	2.64E-09	8.07E-08	3.19
LAMB3	1.67	5.46E-06	7.90E-05	3.18
OGFRL1	1.67	3.41E-07	6.52E-06	3.18
XAF1	1.67	1.60E-04	1.51E-03	3.18
TARP	1.67	1.72E-02	6.65E-02	3.18
RNF145	1.67	1.53E-20	2.73E-18	3.18
STX11	1.66	3.39E-09	1.03E-07	3.17
LPL	1.66	3.27E-08	7.68E-07	3.17
GREM1	1.66	1.65E-06	2.70E-05	3.17
SYNJ2	1.66	7.42E-05	7.76E-04	3.17
PNPLA1	1.66	1.46E-03	9.45E-03	3.16
SPRY2	1.66	1.69E-08	4.33E-07	3.16
CISH	1.66	2.11E-04	1.90E-03	3.16

P2RX7	1.66	6.42E-06	9.08E-05	3.16
ABCB4	1.66	6.47E-09	1.82E-07	3.16
ZHX2	1.65	6.09E-08	1.36E-06	3.15
P4HA2	1.65	2.13E-09	6.67E-08	3.14
GZMB	1.65	2.10E-02	7.73E-02	3.14
RHOF	1.65	1.41E-06	2.34E-05	3.14
TCONS_00029157	1.64	2.74E-02	9.50E-02	3.13
FOSL2	1.64	4.00E-09	1.18E-07	3.12
GPSM1	1.64	7.53E-08	1.65E-06	3.12
EGR2	1.64	1.37E-17	1.79E-15	3.11
FYN	1.64	6.05E-10	2.15E-08	3.11
SLC7A1	1.63	1.11E-12	6.58E-11	3.10
ZEB1	1.63	8.24E-04	5.88E-03	3.10
ACSL1	1.63	2.64E-05	3.16E-04	3.09
KBTBD8	1.62	4.88E-11	2.14E-09	3.08
RASL11A	1.62	1.29E-03	8.53E-03	3.07
RASGEF1A	1.62	6.81E-04	5.01E-03	3.07
MT2A	1.61	2.49E-04	2.18E-03	3.06
CD40	1.61	7.98E-06	1.10E-04	3.05
KREMEN1	1.61	2.40E-05	2.93E-04	3.05
EIF4E	1.61	2.46E-15	2.20E-13	3.04
PANX1	1.60	1.43E-14	1.16E-12	3.04
HMGCS1	1.60	1.04E-10	4.30E-09	3.03
NOP16	1.60	2.42E-13	1.63E-11	3.03
SRC	1.60	9.71E-14	6.95E-12	3.03
KYNU	1.60	1.18E-06	1.99E-05	3.02
B4GALT1	1.59	2.17E-08	5.36E-07	3.02
BNIP3	1.59	6.28E-09	1.77E-07	3.02
PTPN12	1.59	2.51E-12	1.40E-10	3.01
GSTM3	1.59	4.63E-03	2.38E-02	3.01
CCNA1	1.59	4.72E-04	3.65E-03	3.01
NUMB	1.59	1.27E-18	1.85E-16	3.00
SLC16A10	1.58	1.20E-03	7.96E-03	3.00
PTPN1	1.58	1.70E-09	5.50E-08	2.99
MYOF	1.58	4.22E-17	4.99E-15	2.99

MCOLN2	1.58	1.28E-05	1.68E-04	2.99
WNT5A	1.58	1.91E-03	1.17E-02	2.98
STK17A	1.57	1.74E-13	1.19E-11	2.97
STAC2	1.57	1.60E-02	6.31E-02	2.97
SLC2A6	1.57	1.52E-05	1.97E-04	2.96
NEU4	1.57	1.59E-03	1.01E-02	2.96
TUBB2A	1.57	4.14E-11	1.85E-09	2.96
AMZ1	1.57	9.38E-05	9.52E-04	2.96
EIF2AK2	1.56	2.41E-05	2.93E-04	2.95
CD69	1.56	1.18E-02	4.98E-02	2.95
ARL10	1.56	1.71E-04	1.59E-03	2.95
PIM3	1.56	3.34E-07	6.39E-06	2.94
PNPT1	1.55	5.14E-05	5.72E-04	2.94
KBTBD12	1.55	4.20E-04	3.34E-03	2.93
ARNTL2	1.55	2.02E-06	3.24E-05	2.92
CCR5	1.55	5.64E-14	4.13E-12	2.92
RAPGEF2	1.54	2.19E-06	3.46E-05	2.92
TTC39B	1.54	4.23E-10	1.58E-08	2.91
ITGAV	1.54	1.22E-11	6.11E-10	2.91
FNIP2	1.54	2.08E-09	6.52E-08	2.91
ACSL5	1.54	6.94E-15	5.88E-13	2.91
HERC6	1.54	2.49E-05	3.02E-04	2.91
APOBEC3A	1.54	9.58E-04	6.64E-03	2.91
HMGCR	1.54	1.05E-10	4.35E-09	2.91
CHSY1	1.54	1.12E-09	3.79E-08	2.91
PSTPIP2	1.54	1.33E-04	1.29E-03	2.90
YRDC	1.54	2.24E-08	5.47E-07	2.90
LRRC8B	1.53	7.66E-12	4.00E-10	2.90
ZNF674-AS1	1.53	2.99E-03	1.69E-02	2.89
ADGRE5	1.53	1.15E-17	1.54E-15	2.89
IRF7	1.53	2.46E-06	3.86E-05	2.89
SNX10	1.53	2.19E-10	8.46E-09	2.89
ARL5B	1.53	2.33E-04	2.07E-03	2.88
RIPK2	1.52	1.88E-07	3.79E-06	2.87
OAS3	1.52	3.64E-06	5.50E-05	2.87
IFIH1	1.52	3.28E-04	2.74E-03	2.87

CFB	1.52	2.39E-04	2.11E-03	2.87
IFIT5	1.52	4.32E-05	4.90E-04	2.87
PCDHGC3	1.52	4.35E-04	3.43E-03	2.86
DTX4	1.52	3.36E-10	1.27E-08	2.86
GNAI1	1.52	4.03E-03	2.13E-02	2.86
LBH	1.51	6.70E-03	3.19E-02	2.85
FAM83G	1.51	1.19E-06	2.01E-05	2.85
SRXN1	1.51	2.27E-10	8.71E-09	2.85
PRKAG2	1.51	5.70E-09	1.63E-07	2.84
TNFAIP8L3	1.51	2.84E-04	2.43E-03	2.84
ABCB5	1.51	2.33E-02	8.39E-02	2.84
TRIM36	1.50	6.20E-06	8.82E-05	2.84
MYBPH	1.50	2.41E-03	1.41E-02	2.83
ALCAM	1.50	3.75E-09	1.11E-07	2.83
CBLB	1.50	1.37E-05	1.79E-04	2.83
HLX	1.50	1.40E-08	3.64E-07	2.82
HIVEP1	1.49	3.64E-08	8.47E-07	2.82
FFAR2	1.49	1.26E-03	8.31E-03	2.81
TCF7L2	1.49	8.06E-05	8.32E-04	2.80
CLIP2	1.48	5.88E-10	2.11E-08	2.80
DIXDC1	1.48	8.91E-04	6.26E-03	2.79
TSPAN5	1.48	1.68E-02	6.55E-02	2.78
TP53INP2	1.48	1.14E-08	3.07E-07	2.78
ASAP2	1.47	2.18E-04	1.96E-03	2.77
TNFRSF10A	1.47	1.34E-07	2.79E-06	2.77
TMEM26	1.47	4.19E-04	3.34E-03	2.76
TRAF3IP2	1.46	3.61E-09	1.09E-07	2.76
STARD4	1.46	4.73E-08	1.07E-06	2.76
NEDD4L	1.46	1.04E-09	3.56E-08	2.76
ARL4A	1.46	2.18E-07	4.31E-06	2.76
TNFSF15	1.46	4.06E-09	1.19E-07	2.75
ZCCHC2	1.46	2.45E-11	1.14E-09	2.75
TREM1	1.46	1.73E-06	2.83E-05	2.75
HSD11B1	1.46	3.50E-03	1.92E-02	2.75
URB2	1.46	1.46E-10	5.75E-09	2.75
CFAP58-AS1	1.46	5.41E-04	4.12E-03	2.75

ADCY6	1.46	9.24E-04	6.45E-03	2.74
NCS1	1.45	3.13E-14	2.38E-12	2.74
TJP2	1.45	2.15E-05	2.65E-04	2.73
ACVR2A	1.45	1.83E-08	4.62E-07	2.73
SPAG1	1.45	3.65E-05	4.20E-04	2.73
HERC5	1.44	1.80E-03	1.12E-02	2.72
GRAMD3	1.44	3.93E-04	3.17E-03	2.72
VLDLR	1.44	3.09E-03	1.73E-02	2.71
FNDC3B	1.44	2.11E-08	5.24E-07	2.71
ST3GAL4	1.43	6.27E-05	6.73E-04	2.70
FLT1	1.43	1.79E-09	5.71E-08	2.70
NRIP3	1.43	3.93E-07	7.39E-06	2.69
PMEPA1	1.43	8.98E-09	2.47E-07	2.69
AMPD3	1.43	5.60E-06	8.10E-05	2.69
RDX	1.43	1.67E-08	4.28E-07	2.69
PLEK	1.43	6.68E-14	4.84E-12	2.69
CDS1	1.42	6.57E-03	3.14E-02	2.68
LCP2	1.42	9.53E-07	1.65E-05	2.68
PLSCR1	1.42	1.16E-06	1.97E-05	2.67
RNF19A	1.42	1.19E-13	8.38E-12	2.67
LRIG1	1.42	5.44E-05	5.97E-04	2.67
ZNFX1	1.41	1.35E-07	2.80E-06	2.66
FZD7	1.41	1.81E-05	2.30E-04	2.66
MIR17HG	1.41	2.41E-02	8.61E-02	2.66
BCL2	1.41	2.92E-06	4.51E-05	2.65
BID	1.41	5.55E-10	2.00E-08	2.65
APOL6	1.41	1.26E-05	1.66E-04	2.65
DDR1	1.41	2.12E-05	2.62E-04	2.65
CARD10	1.41	2.85E-02	9.78E-02	2.65
NFKBIZ	1.41	9.70E-05	9.80E-04	2.65
SLC5A10	1.40	5.87E-03	2.89E-02	2.64
CCL23	1.40	2.92E-03	1.65E-02	2.64
GTPBP4	1.40	1.39E-08	3.62E-07	2.64
MATK	1.40	1.43E-07	2.94E-06	2.63
SAV1	1.39	6.87E-07	1.22E-05	2.63
GBP3	1.39	1.64E-03	1.03E-02	2.62

STAT1	1.39	1.09E-09	3.70E-08	2.62
OLIG1	1.39	3.62E-03	1.97E-02	2.61
PEAK1	1.38	1.94E-11	9.18E-10	2.61
MTFP1	1.38	8.83E-08	1.91E-06	2.61
RPS6KA3	1.38	3.51E-11	1.58E-09	2.60
ago-02	1.38	3.54E-09	1.07E-07	2.60
RUNX1	1.38	2.38E-13	1.61E-11	2.60
HTRA3	1.38	1.04E-02	4.53E-02	2.60
ABLIM1	1.37	3.13E-05	3.67E-04	2.59
HIF1A	1.37	3.24E-06	4.94E-05	2.59
GRB10	1.37	1.03E-05	1.40E-04	2.58
ITPR3	1.37	5.40E-04	4.11E-03	2.58
MAK16	1.37	2.16E-10	8.36E-09	2.58
MN1	1.36	3.48E-03	1.91E-02	2.57
MCOLN3	1.36	2.12E-06	3.37E-05	2.57
FAM57A	1.36	1.95E-08	4.89E-07	2.57
TEX10	1.36	6.92E-11	2.92E-09	2.57
TAP1	1.36	3.90E-07	7.35E-06	2.57
ANKRD16	1.36	5.68E-05	6.19E-04	2.56
POU2AF1	1.36	3.96E-03	2.11E-02	2.56
FAM101B	1.36	1.73E-08	4.41E-07	2.56
SH2D3A	1.35	2.58E-02	9.06E-02	2.56
LINC01588	1.35	9.13E-04	6.38E-03	2.55
FAM210A	1.35	3.60E-11	1.62E-09	2.55
TMEM158	1.35	1.17E-06	1.98E-05	2.54
RCAN1	1.34	2.09E-05	2.60E-04	2.54
TXN	1.34	6.22E-08	1.38E-06	2.54
NIP7	1.34	4.42E-11	1.95E-09	2.54
SPHK1	1.34	9.29E-12	4.77E-10	2.53
RASGEF1B	1.34	1.03E-13	7.30E-12	2.53
LUCAT1	1.33	6.41E-06	9.08E-05	2.52
NCR3LG1	1.33	2.33E-03	1.37E-02	2.52
MEI1	1.33	5.43E-03	2.70E-02	2.52
ADAM17	1.33	1.59E-08	4.10E-07	2.52
PSMA6	1.33	7.14E-10	2.51E-08	2.51
POLR3D	1.33	1.96E-08	4.92E-07	2.51

C1S	1.32	2.53E-02	8.95E-02	2.50
SMAGP	1.32	7.25E-03	3.42E-02	2.49
LDHA	1.32	9.42E-13	5.65E-11	2.49
SLC43A3	1.32	3.85E-13	2.48E-11	2.49
FAM129B	1.31	1.72E-11	8.24E-10	2.49
HK2	1.31	1.51E-11	7.36E-10	2.49
SHANK3	1.31	5.83E-03	2.87E-02	2.49
LINC01004	1.31	1.58E-04	1.49E-03	2.49
IL32	1.31	3.21E-03	1.78E-02	2.49
PARP9	1.31	3.10E-04	2.61E-03	2.48
EVC2	1.31	2.65E-02	9.26E-02	2.48
POMZP3	1.31	1.96E-02	7.34E-02	2.48
SLC9B2	1.31	1.74E-02	6.72E-02	2.48
SQLE	1.31	1.08E-07	2.30E-06	2.47
CD276	1.31	3.24E-12	1.80E-10	2.47
BZW1	1.31	2.07E-12	1.18E-10	2.47
ARL8B	1.30	1.58E-08	4.07E-07	2.47
ARAP2	1.30	1.95E-05	2.44E-04	2.47
CEP170	1.30	5.41E-08	1.22E-06	2.47
SPAG9	1.30	3.73E-09	1.11E-07	2.47
KIF21A	1.30	3.63E-03	1.97E-02	2.47
UAP1	1.30	4.08E-09	1.20E-07	2.47
ALDH1B1	1.30	4.22E-08	9.74E-07	2.46
ZBTB10	1.30	3.08E-04	2.59E-03	2.46
PCGF5	1.30	1.19E-11	5.95E-10	2.46
LINC00936	1.30	2.22E-04	1.99E-03	2.46
EAF1	1.30	2.23E-09	6.94E-08	2.46
ARHGAP31	1.30	2.58E-09	7.94E-08	2.46
SERPINB8	1.30	5.18E-10	1.89E-08	2.46
LSS	1.30	2.32E-05	2.84E-04	2.46
MICALL1	1.30	4.28E-07	7.97E-06	2.46
ADGRE2	1.30	1.16E-06	1.97E-05	2.46
ANXA5	1.29	2.26E-11	1.06E-09	2.44
MTHFD2	1.29	8.09E-08	1.76E-06	2.44
GSN-AS1	1.29	1.03E-02	4.50E-02	2.44
NAV2	1.28	3.85E-04	3.12E-03	2.44

LOC100133091	1.28	1.43E-02	5.82E-02	2.43
TPM4	1.28	1.45E-12	8.43E-11	2.43
UBE2J1	1.28	2.46E-07	4.81E-06	2.43
ARFGAP3	1.28	4.57E-10	1.69E-08	2.43
TRIP10	1.28	4.02E-05	4.58E-04	2.43
SIK1	1.28	3.03E-06	4.67E-05	2.43
DSE	1.28	8.46E-07	1.48E-05	2.42
DCUN1D3	1.28	1.29E-03	8.50E-03	2.42
HDAC9	1.27	7.94E-05	8.22E-04	2.42
KIAA0020	1.27	2.93E-10	1.12E-08	2.42
CEBPB	1.27	1.62E-07	3.32E-06	2.41
SLC25A32	1.27	5.91E-09	1.68E-07	2.41
CDK17	1.27	4.64E-08	1.06E-06	2.41
ELOVL5	1.27	1.07E-09	3.63E-08	2.41
HDGFRP3	1.27	1.71E-04	1.59E-03	2.41
CXCL3	1.26	1.46E-03	9.45E-03	2.40
ACTN1	1.26	4.27E-10	1.59E-08	2.40
PRDM8	1.26	1.12E-02	4.81E-02	2.40
BYSL	1.26	4.19E-08	9.68E-07	2.40
AEN	1.26	1.13E-08	3.05E-07	2.39
VSTM4	1.26	2.55E-02	8.98E-02	2.39
FAM126A	1.26	2.73E-06	4.25E-05	2.39
GPR153	1.25	7.55E-05	7.90E-04	2.39
CEP170B	1.25	7.52E-10	2.64E-08	2.38
MSMO1	1.25	4.03E-09	1.19E-07	2.38
COL6A1	1.25	1.15E-03	7.71E-03	2.38
TRMT6	1.25	1.47E-06	2.43E-05	2.37
ANKLE2	1.25	1.20E-08	3.20E-07	2.37
SPRED2	1.25	1.22E-07	2.55E-06	2.37
B3GNT5	1.24	6.75E-09	1.89E-07	2.37
ETS1	1.24	8.97E-04	6.29E-03	2.37
SBNO2	1.24	9.09E-04	6.37E-03	2.37
BACH1	1.24	3.85E-04	3.12E-03	2.37
TXNIP	1.24	1.17E-07	2.46E-06	2.37
SRGN	1.24	1.66E-11	8.01E-10	2.36
ADPRHL2	1.24	1.46E-10	5.75E-09	2.36

ZC3HAV1	1.24	6.19E-05	6.65E-04	2.36
LOXL2	1.24	1.31E-02	5.42E-02	2.36
UPP1	1.24	3.75E-09	1.11E-07	2.36
NRP1	1.23	4.69E-08	1.07E-06	2.35
NUP188	1.23	5.04E-10	1.85E-08	2.35
UTP15	1.23	2.23E-08	5.47E-07	2.35
LDLR	1.23	1.10E-04	1.08E-03	2.35
TRIM24	1.23	4.68E-06	6.90E-05	2.34
ARID5B	1.22	2.06E-06	3.29E-05	2.34
ZNF654	1.22	7.30E-07	1.29E-05	2.33
SLC4A5	1.22	1.38E-07	2.87E-06	2.33
CLIC4	1.22	6.01E-05	6.51E-04	2.33
TUBB6	1.22	1.97E-09	6.22E-08	2.33
FAM107B	1.22	1.96E-07	3.93E-06	2.33
OTUD1	1.22	4.13E-04	3.31E-03	2.33
CHML	1.22	4.61E-03	2.37E-02	2.33
CD38	1.22	6.06E-03	2.96E-02	2.33
SH3BP5	1.22	4.55E-08	1.04E-06	2.33
LINC-PINT	1.22	2.23E-05	2.74E-04	2.32
IL1RAP	1.21	1.53E-05	1.97E-04	2.32
PTP4A1	1.21	2.50E-07	4.87E-06	2.32
TAF13	1.21	9.32E-07	1.62E-05	2.31
KDM6B	1.21	2.14E-03	1.29E-02	2.31
TCTEX1D4	1.21	1.69E-02	6.56E-02	2.31
VIM	1.21	2.10E-10	8.14E-09	2.31
ETV3	1.21	4.02E-03	2.13E-02	2.31
ALDH1A2	1.21	9.95E-03	4.40E-02	2.31
NFKB2	1.20	1.21E-03	8.02E-03	2.31
EML4	1.20	1.01E-08	2.75E-07	2.30
NFAT5	1.20	2.23E-05	2.74E-04	2.30
CDYL2	1.20	2.17E-03	1.30E-02	2.30
NIFK	1.20	2.73E-08	6.58E-07	2.29
PI4K2B	1.20	3.60E-04	2.94E-03	2.29
RABGEF1	1.20	1.47E-09	4.80E-08	2.29
ACTG1	1.19	1.06E-07	2.25E-06	2.29
MCL1	1.19	6.94E-06	9.73E-05	2.29

SLC35E4	1.19	1.21E-06	2.03E-05	2.29
PPIF	1.19	1.06E-07	2.25E-06	2.28
NLRP3	1.19	1.45E-05	1.88E-04	2.28
GK	1.19	2.14E-08	5.29E-07	2.28
KHDRBS3	1.19	5.89E-03	2.89E-02	2.28
PNO1	1.19	3.53E-08	8.24E-07	2.28
ZDHHC9	1.19	7.36E-07	1.30E-05	2.28
MLLT4	1.19	3.96E-04	3.20E-03	2.28
UXS1	1.19	9.82E-05	9.90E-04	2.28
PFKP	1.19	6.42E-08	1.42E-06	2.27
CD300E	1.19	9.48E-05	9.61E-04	2.27
PPA1	1.18	4.00E-04	3.22E-03	2.27
ABCE1	1.18	3.69E-07	6.99E-06	2.27
SEMA3C	1.18	3.50E-05	4.05E-04	2.26
NAA50	1.18	5.91E-07	1.06E-05	2.26
PHACTR2	1.17	3.29E-06	5.01E-05	2.26
ST3GAL6-AS1	1.17	4.40E-03	2.29E-02	2.26
NFATC1	1.17	2.21E-09	6.91E-08	2.25
ATP1A1	1.17	4.95E-09	1.43E-07	2.25
RASAL2-AS1	1.17	1.47E-02	5.94E-02	2.25
MESDC1	1.17	2.98E-06	4.59E-05	2.24
NBN	1.16	2.50E-03	1.46E-02	2.24
MYBPC3	1.16	2.00E-02	7.45E-02	2.24
LYN	1.16	1.85E-04	1.70E-03	2.24
SUSD6	1.16	6.29E-06	8.93E-05	2.24
SP140	1.16	9.65E-03	4.30E-02	2.24
TSR1	1.16	1.04E-06	1.78E-05	2.23
BLZF1	1.16	5.36E-05	5.91E-04	2.23
PSD3	1.16	2.42E-03	1.42E-02	2.23
MITF	1.16	4.18E-06	6.23E-05	2.23
THBD	1.16	1.52E-06	2.52E-05	2.23
DCP1A	1.15	1.72E-05	2.20E-04	2.23
NAA15	1.15	6.43E-08	1.42E-06	2.22
MRAS	1.15	2.35E-06	3.70E-05	2.22
TCAF2	1.15	8.45E-05	8.69E-04	2.22
MYADM	1.15	7.57E-10	2.65E-08	2.22

TC2N	1.15	2.04E-02	7.57E-02	2.22
NCK2	1.15	3.67E-06	5.53E-05	2.21
ENTPD7	1.15	8.48E-08	1.84E-06	2.21
PML	1.15	6.16E-04	4.60E-03	2.21
ST3GAL2	1.14	4.70E-07	8.68E-06	2.21
SP110	1.14	3.23E-03	1.79E-02	2.21
RAB8B	1.14	4.36E-07	8.09E-06	2.21
IL4I1	1.14	1.11E-05	1.49E-04	2.20
C1orf122	1.14	1.46E-04	1.39E-03	2.20
PKD2	1.14	1.01E-06	1.74E-05	2.20
RHBDF1	1.14	2.52E-04	2.20E-03	2.20
PHLPP2	1.14	1.77E-05	2.24E-04	2.20
MAPKAPK2	1.13	2.17E-08	5.36E-07	2.19
TSPAN33	1.13	7.62E-04	5.50E-03	2.19
TDRD7	1.13	3.42E-04	2.83E-03	2.19
BAZ1A	1.13	2.25E-04	2.01E-03	2.19
KLF10	1.13	6.91E-09	1.92E-07	2.19
RRS1	1.13	1.68E-07	3.41E-06	2.19
RAP1B	1.13	4.28E-08	9.86E-07	2.19
BHLHE40	1.13	1.20E-09	4.02E-08	2.18
ZFYVE16	1.13	9.33E-09	2.56E-07	2.18
PERP	1.12	1.62E-02	6.37E-02	2.18
FJX1	1.12	3.16E-04	2.66E-03	2.17
ENPP2	1.12	9.29E-05	9.45E-04	2.17
PPRC1	1.12	1.36E-06	2.28E-05	2.17
KLF6	1.12	7.03E-08	1.54E-06	2.17
NPC1	1.12	1.87E-09	5.96E-08	2.17
FEM1C	1.12	9.87E-07	1.70E-05	2.17
C3	1.12	3.30E-04	2.75E-03	2.17
HESX1	1.11	2.12E-02	7.81E-02	2.16
PPP1R15B	1.11	1.13E-04	1.11E-03	2.16
CD226	1.11	1.80E-04	1.66E-03	2.15
FILIP1L	1.10	3.07E-03	1.73E-02	2.15
GNPNAT1	1.10	4.40E-07	8.15E-06	2.15
CCNJ	1.10	5.72E-06	8.24E-05	2.15
ABCC1	1.10	4.59E-09	1.33E-07	2.15

METRNL	1.10	1.78E-06	2.90E-05	2.15
CIRH1A	1.10	2.04E-08	5.10E-07	2.14
EPT1	1.10	1.54E-05	1.99E-04	2.14
SGTB	1.10	9.17E-05	9.35E-04	2.14
FZD6	1.10	6.91E-03	3.27E-02	2.14
LONRF1	1.09	2.66E-05	3.17E-04	2.14
PPP3CC	1.09	4.49E-04	3.51E-03	2.14
C14orf169	1.09	3.53E-07	6.73E-06	2.14
SFXN1	1.09	5.13E-06	7.48E-05	2.13
MLLT6	1.09	5.08E-05	5.67E-04	2.13
ANPEP	1.09	4.06E-09	1.19E-07	2.13
DTX3L	1.09	5.49E-04	4.16E-03	2.13
MTRR	1.09	8.44E-08	1.83E-06	2.13
MPZL1	1.09	3.41E-04	2.83E-03	2.13
TP53BP2	1.09	3.11E-06	4.78E-05	2.13
EZH2	1.09	2.30E-03	1.36E-02	2.13
NOC3L	1.09	1.38E-05	1.81E-04	2.12
IL4R	1.09	6.26E-07	1.12E-05	2.12
CYP51A1	1.08	2.05E-08	5.10E-07	2.12
PLEKHO1	1.08	5.61E-06	8.10E-05	2.12
SEH1L	1.08	2.98E-08	7.09E-07	2.12
MSC-AS1	1.08	1.03E-05	1.39E-04	2.12
FGFR1	1.08	5.56E-04	4.21E-03	2.12
NLE1	1.08	5.56E-05	6.08E-04	2.11
PTPRJ	1.08	1.06E-04	1.06E-03	2.11
HMGA1	1.08	2.60E-05	3.12E-04	2.11
BRIX1	1.08	5.75E-06	8.27E-05	2.11
LY6E	1.07	1.25E-05	1.66E-04	2.11
MOB3C	1.07	7.86E-05	8.16E-04	2.10
RIF1	1.07	2.99E-04	2.54E-03	2.10
STK38L	1.07	1.32E-04	1.28E-03	2.10
SPRED1	1.07	5.75E-07	1.04E-05	2.10
PSD4	1.07	4.04E-05	4.60E-04	2.10
FAM208B	1.07	1.62E-04	1.52E-03	2.10
FAM129A	1.07	1.03E-02	4.52E-02	2.10
TNFRSF10D	1.07	4.82E-04	3.72E-03	2.10

STARD8	1.07	3.02E-08	7.17E-07	2.09
RAP2C	1.06	2.86E-05	3.40E-04	2.09
NXT1	1.06	1.30E-05	1.71E-04	2.09
ITGA5	1.06	5.58E-09	1.60E-07	2.08
PITPNB	1.06	3.18E-08	7.50E-07	2.08
SH2B3	1.05	1.01E-08	2.75E-07	2.08
PPARD	1.05	1.07E-07	2.27E-06	2.08
MMP14	1.05	9.10E-04	6.37E-03	2.08
CDKN2B	1.05	4.76E-04	3.68E-03	2.07
CNIH4	1.05	1.64E-04	1.53E-03	2.07
IFI6	1.05	3.67E-04	2.99E-03	2.07
FUT4	1.05	2.59E-05	3.12E-04	2.07
DPH3	1.05	2.15E-07	4.26E-06	2.07
SGCB	1.05	4.89E-03	2.48E-02	2.07
TSHZ3	1.05	2.05E-06	3.27E-05	2.07
SNX9	1.05	8.18E-08	1.78E-06	2.07
FAM60A	1.05	5.03E-03	2.54E-02	2.06
YWHAG	1.04	1.18E-07	2.47E-06	2.06
GOLT1B	1.04	1.24E-07	2.57E-06	2.06
BEND3	1.04	2.43E-04	2.14E-03	2.06
DGKH	1.04	9.04E-04	6.34E-03	2.05
MYO1E	1.04	1.21E-06	2.03E-05	2.05
TWISTNB	1.04	6.54E-06	9.23E-05	2.05
TRIM25	1.04	3.64E-03	1.97E-02	2.05
LARP1B	1.03	1.07E-04	1.06E-03	2.05
LTV1	1.03	2.01E-05	2.51E-04	2.04
RELB	1.03	9.72E-05	9.80E-04	2.04
DNAH17	1.03	1.36E-02	5.56E-02	2.04
KITLG	1.03	5.15E-06	7.51E-05	2.04
NR4A1	1.03	2.53E-03	1.47E-02	2.04
ACVR1	1.03	2.35E-04	2.08E-03	2.04
FBXL5	1.03	7.20E-06	1.01E-04	2.04
SLC37A3	1.02	6.69E-03	3.19E-02	2.03
ATP10A	1.02	2.13E-03	1.29E-02	2.03
ABCA1	1.02	3.48E-08	8.13E-07	2.03
LMNB1	1.02	4.26E-04	3.38E-03	2.03

DMPK	1.02	2.32E-02	8.36E-02	2.03
FLNA	1.02	4.05E-08	9.38E-07	2.03
TRG-AS1	1.02	1.68E-03	1.06E-02	2.03
NABP1	1.02	5.18E-08	1.17E-06	2.03
IDI2	1.02	4.92E-03	2.50E-02	2.02
PDE4DIP	1.02	8.71E-04	6.16E-03	2.02
C10orf10	1.01	2.28E-03	1.35E-02	2.02
GPRIN1	1.01	4.77E-03	2.44E-02	2.02
LOC101926963	1.01	1.51E-03	9.71E-03	2.02
VASP	1.01	9.54E-07	1.65E-05	2.02
STRIP2	1.01	1.55E-03	9.91E-03	2.02
PEA15	1.01	1.41E-07	2.91E-06	2.01
SH2B2	1.01	2.01E-03	1.22E-02	2.01
CLDN12	1.01	2.20E-04	1.97E-03	2.01
RAC2	1.01	7.88E-05	8.18E-04	2.01
ATF5	1.01	2.09E-05	2.60E-04	2.01
RHOC	1.01	8.65E-06	1.19E-04	2.01
MAP1LC3A	1.00	1.60E-02	6.31E-02	2.00
CTPS1	1.00	9.01E-06	1.23E-04	2.00
SRGAP1	1.00	5.40E-03	2.69E-02	2.00
PPP1R13B	1.00	2.24E-03	1.33E-02	2.00

Differentially expressed genes (padj < 0.1, log2FC ≥ 1) in the anti-CCL2 Ab 4h vs nil 4h comparison (dataset 1)
