# Supplementary material for: Transcriptome Profiling of Human Monocyte-Derived Macrophages Upon CCL2 Neutralization Reveals an Association Between Activation of Innate Immune Pathways and Restriction of HIV-1 Gene Expression
Source: Front Immunol. 2020 Sep 18;11:2129. doi: 10.3389/fimmu.2020.02129 (PMC7531389; doi:10.3389/fimmu.2020.02129)
Supplement: Supplementary file 1 [file Data_Sheet_1.zip › Supplementary tables/Covino et al_Supplementary Table 2.pdf]

Supplementary Table 2. List of down-regulated genes in MDMs exposed to anti-CCL2 Ab for 4 hours.

gene	*log2FC*	*pvalue*	*padj*	*FC*
WIPI1	-1.00	5.97E-07	1.07E-05	0.50
APBB1IP	-1.00	9.66E-07	1.67E-05	0.50
TPRN	-1.00	4.86E-04	3.74E-03	0.50
ASB16-AS1	-1.00	6.14E-03	2.98E-02	0.50
C1orf233	-1.00	3.41E-05	3.95E-04	0.50
HTR7P1	-1.00	1.38E-03	9.01E-03	0.50
PCMTD2	-1.00	8.63E-06	1.19E-04	0.50
VPS26B	-1.00	5.22E-06	7.59E-05	0.50
RGAG4	-1.00	7.46E-07	1.32E-05	0.50
USP20	-1.00	1.23E-05	1.63E-04	0.50
CTNNBIP1	-1.00	1.45E-06	2.41E-05	0.50
HDDC3	-1.00	6.11E-04	4.58E-03	0.50
ERMAP	-1.00	2.61E-05	3.13E-04	0.50
ZFYVE26	-1.01	3.43E-04	2.83E-03	0.50
TTLL12	-1.01	5.12E-05	5.70E-04	0.50
MS4A14	-1.01	6.13E-04	4.58E-03	0.50
ZMAT5	-1.01	3.27E-04	2.73E-03	0.50
C17orf59	-1.01	7.31E-05	7.69E-04	0.50
R3HDM2	-1.01	3.96E-06	5.92E-05	0.50
ACBD4	-1.01	1.32E-02	5.45E-02	0.50
FAM78A	-1.01	1.46E-04	1.39E-03	0.50
IER5L	-1.01	3.70E-03	2.00E-02	0.50
BACE1	-1.01	2.81E-03	1.60E-02	0.50
LOC103344931	-1.01	1.91E-02	7.21E-02	0.49
GLCE	-1.02	2.24E-03	1.33E-02	0.49
OGFOD2	-1.02	7.02E-04	5.12E-03	0.49
ADRBK1	-1.02	1.07E-05	1.45E-04	0.49
SLC9A3R1	-1.02	5.13E-04	3.92E-03	0.49
ZNF253	-1.02	8.84E-03	4.01E-02	0.49
MAN1C1	-1.02	3.82E-04	3.10E-03	0.49
NMRAL1	-1.02	4.74E-06	6.97E-05	0.49
ANO8	-1.03	3.17E-03	1.77E-02	0.49

NCAPD2	-1.03	4.25E-06	6.33E-05	0.49
HENMT1	-1.03	4.77E-03	2.44E-02	0.49
PLA2G15	-1.03	5.20E-05	5.77E-04	0.49
ZNF717	-1.03	1.07E-02	4.65E-02	0.49
EEF2K	-1.03	8.47E-04	6.02E-03	0.49
NLRC4	-1.03	4.70E-04	3.64E-03	0.49
MSH2	-1.03	2.38E-03	1.40E-02	0.49
DOK3	-1.03	1.48E-07	3.05E-06	0.49
GLCCI1	-1.03	6.55E-03	3.13E-02	0.49
COLEC12	-1.03	1.75E-02	6.74E-02	0.49
CBR4	-1.03	2.09E-04	1.89E-03	0.49
ATG2A	-1.04	7.41E-05	7.76E-04	0.49
ZNF449	-1.04	6.26E-04	4.66E-03	0.49
CD180	-1.04	8.08E-04	5.78E-03	0.49
ATP10D	-1.04	7.70E-05	8.02E-04	0.49
TAB1	-1.04	4.12E-07	7.70E-06	0.49
CKLF	-1.04	4.41E-03	2.30E-02	0.49
DISP1	-1.04	9.51E-03	4.25E-02	0.49
C2orf74	-1.04	7.54E-04	5.46E-03	0.49
NMRK1	-1.04	1.04E-03	7.08E-03	0.49
LOC100294145	-1.04	4.88E-04	3.75E-03	0.49
PINK1	-1.04	5.86E-06	8.36E-05	0.49
ZNF75D	-1.05	4.38E-04	3.45E-03	0.48
DEF6	-1.05	6.80E-04	5.00E-03	0.48
XRRA1	-1.05	1.55E-02	6.15E-02	0.48
TARBP1	-1.05	5.28E-03	2.64E-02	0.48
PEX6	-1.05	1.31E-04	1.27E-03	0.48
MAN2A2	-1.05	1.74E-06	2.85E-05	0.48
MGME1	-1.05	5.22E-05	5.79E-04	0.48
CMTM7	-1.05	1.67E-05	2.14E-04	0.48
SCIMP	-1.05	4.24E-04	3.37E-03	0.48
GLTSCR2	-1.05	2.25E-08	5.49E-07	0.48
IGF1	-1.05	1.03E-04	1.03E-03	0.48
NUP85	-1.05	7.18E-08	1.57E-06	0.48
SLC22A5	-1.05	2.28E-04	2.03E-03	0.48
FCHO2	-1.06	6.16E-04	4.60E-03	0.48

AMDHD2	-1.06	6.60E-08	1.45E-06	0.48
CORO2A	-1.06	5.70E-05	6.21E-04	0.48
CD300LF	-1.06	5.37E-07	9.81E-06	0.48
NOTCH1	-1.06	9.39E-04	6.54E-03	0.48
NAPRT	-1.06	7.55E-07	1.33E-05	0.48
FRS3	-1.06	1.62E-03	1.03E-02	0.48
PDCD4	-1.07	2.09E-06	3.32E-05	0.48
CACFD1	-1.07	1.88E-02	7.14E-02	0.48
GIGYF1	-1.07	1.64E-04	1.53E-03	0.48
HERC2P2	-1.07	1.11E-02	4.77E-02	0.48
BDH1	-1.07	4.44E-03	2.31E-02	0.48
MORC2-AS1	-1.07	1.81E-02	6.93E-02	0.48
FAM214A	-1.07	1.06E-03	7.18E-03	0.48
RGS19	-1.07	2.19E-07	4.33E-06	0.48
KCNJ5	-1.07	1.32E-08	3.47E-07	0.48
CALCOCO1	-1.07	7.86E-06	1.09E-04	0.48
NLRX1	-1.07	3.23E-06	4.94E-05	0.48
SDR39U1	-1.07	1.09E-02	4.72E-02	0.48
TRAF3IP3	-1.07	3.92E-03	2.09E-02	0.48
LINC01503	-1.07	4.15E-04	3.32E-03	0.48
ARHGEF18	-1.07	1.78E-06	2.90E-05	0.48
GPR146	-1.08	2.07E-03	1.26E-02	0.47
ZNF524	-1.08	3.58E-04	2.93E-03	0.47
ERCC2	-1.08	1.46E-04	1.39E-03	0.47
CRIPAK	-1.08	1.09E-04	1.07E-03	0.47
BIVM	-1.08	7.37E-03	3.46E-02	0.47
GATSL3	-1.08	1.04E-03	7.08E-03	0.47
TNRC18	-1.08	7.87E-07	1.39E-05	0.47
RECQL5	-1.08	5.44E-05	5.97E-04	0.47
PIDD1	-1.09	4.63E-03	2.38E-02	0.47
PC	-1.09	1.58E-03	1.00E-02	0.47
SIPA1	-1.09	8.75E-05	8.95E-04	0.47
CXCR4	-1.09	3.40E-04	2.82E-03	0.47
ZNF862	-1.09	8.17E-04	5.83E-03	0.47
TSNARE1	-1.09	1.78E-04	1.65E-03	0.47
TNFSF12	-1.09	1.04E-06	1.78E-05	0.47

TOM1L2	-1.10	2.27E-05	2.78E-04	0.47
GRIN3A	-1.10	1.43E-02	5.80E-02	0.47
SLC8B1	-1.10	8.11E-07	1.42E-05	0.47
THRIL	-1.10	3.40E-03	1.87E-02	0.47
KIF27	-1.10	2.29E-02	8.28E-02	0.47
FLYWCH1	-1.10	2.59E-05	3.12E-04	0.47
PTGFRN	-1.10	1.86E-06	3.01E-05	0.47
SYK	-1.10	6.08E-07	1.09E-05	0.47
SLC46A3	-1.10	8.64E-05	8.85E-04	0.47
LRRC37A4P	-1.10	8.01E-04	5.73E-03	0.47
TIGD2	-1.10	1.28E-03	8.46E-03	0.47
ANKMY1	-1.10	5.44E-04	4.13E-03	0.47
CEP131	-1.10	3.22E-05	3.75E-04	0.47
UBE4B	-1.10	2.53E-08	6.16E-07	0.47
LINC01003	-1.10	1.94E-03	1.19E-02	0.47
CAMK2G	-1.11	7.27E-06	1.01E-04	0.46
DHRS4-AS1	-1.11	1.16E-05	1.55E-04	0.46
C15orf27	-1.11	1.97E-02	7.36E-02	0.46
MYO18A	-1.11	3.95E-06	5.92E-05	0.46
C20orf27	-1.11	3.64E-06	5.50E-05	0.46
FRMD4B	-1.11	3.91E-03	2.09E-02	0.46
AP1B1	-1.11	1.08E-07	2.30E-06	0.46
PPFIBP2	-1.11	1.04E-06	1.78E-05	0.46
C19orf54	-1.11	2.29E-05	2.80E-04	0.46
CLEC3B	-1.11	3.19E-03	1.78E-02	0.46
MBOAT1	-1.11	3.21E-05	3.74E-04	0.46
FOXO4	-1.12	1.37E-04	1.31E-03	0.46
HMOX1	-1.12	2.53E-04	2.20E-03	0.46
FCHO1	-1.12	8.73E-04	6.17E-03	0.46
MTUS1	-1.12	3.45E-03	1.90E-02	0.46
LYNX1	-1.12	1.24E-02	5.19E-02	0.46
TP53INP1	-1.12	1.13E-04	1.11E-03	0.46
FRMD4A	-1.12	3.41E-04	2.82E-03	0.46
CC2D1A	-1.12	1.01E-05	1.37E-04	0.46
KLHL21	-1.12	2.78E-08	6.67E-07	0.46
ADCY7	-1.12	1.01E-08	2.75E-07	0.46

SLC25A10	-1.12	8.16E-03	3.77E-02	0.46
ZNF554	-1.12	7.02E-03	3.32E-02	0.46
LYPLAL1	-1.12	5.80E-05	6.30E-04	0.46
LOC100288637	-1.13	6.71E-03	3.19E-02	0.46
ZNF33B	-1.13	1.03E-03	7.07E-03	0.46
VWCE	-1.13	1.49E-02	5.98E-02	0.46
KLHDC2	-1.13	2.17E-06	3.44E-05	0.46
OLFML2B	-1.13	1.93E-03	1.19E-02	0.46
HAGHL	-1.13	2.71E-06	4.23E-05	0.46
ZNF767P	-1.13	3.32E-04	2.77E-03	0.46
TST	-1.13	5.38E-05	5.91E-04	0.46
PAN2	-1.13	5.12E-05	5.70E-04	0.46
KCNK6	-1.13	1.59E-07	3.27E-06	0.46
ZNF821	-1.13	3.26E-04	2.73E-03	0.46
ARHGAP35	-1.13	6.61E-09	1.85E-07	0.46
LINC00476	-1.13	6.25E-03	3.02E-02	0.46
ACSF2	-1.13	5.85E-04	4.40E-03	0.46
PLCD3	-1.13	8.03E-03	3.72E-02	0.46
HSPA7	-1.13	5.64E-07	1.02E-05	0.46
CCDC88C	-1.13	2.59E-06	4.06E-05	0.46
GAS8	-1.14	6.34E-03	3.05E-02	0.46
C20orf194	-1.14	8.90E-07	1.55E-05	0.46
TRIM65	-1.14	9.49E-07	1.65E-05	0.45
SEMA4C	-1.14	3.21E-03	1.78E-02	0.45
SEPT3	-1.14	8.43E-03	3.87E-02	0.45
DTWD2	-1.14	1.77E-05	2.24E-04	0.45
RB1	-1.14	2.80E-09	8.56E-08	0.45
WASH3P	-1.14	2.78E-02	9.61E-02	0.45
USP51	-1.14	6.48E-03	3.11E-02	0.45
AATBC	-1.14	4.47E-03	2.32E-02	0.45
FAM174B	-1.14	1.73E-02	6.67E-02	0.45
NINL	-1.14	9.82E-04	6.78E-03	0.45
LDLRAD4	-1.14	3.81E-08	8.83E-07	0.45
IMPA2	-1.15	1.74E-04	1.61E-03	0.45
JDP2	-1.15	3.24E-06	4.94E-05	0.45
TFAP4	-1.15	5.87E-04	4.41E-03	0.45

GCHFR	-1.15	2.08E-05	2.60E-04	0.45
RNF135	-1.15	3.12E-06	4.78E-05	0.45
CTDSP2	-1.15	1.32E-08	3.47E-07	0.45
LTB4R	-1.16	8.64E-04	6.12E-03	0.45
SH3RF3	-1.16	5.01E-03	2.54E-02	0.45
MIB2	-1.16	1.53E-04	1.45E-03	0.45
PDXP	-1.16	2.03E-06	3.25E-05	0.45
C14orf93	-1.16	8.08E-04	5.78E-03	0.45
C1orf162	-1.16	1.73E-06	2.84E-05	0.45
ALS2CL	-1.16	7.57E-03	3.54E-02	0.45
TMEM42	-1.16	8.81E-05	9.00E-04	0.45
PAQR4	-1.16	3.84E-07	7.24E-06	0.45
SAMD4A	-1.17	8.53E-04	6.06E-03	0.45
ZNF703	-1.17	4.48E-04	3.51E-03	0.45
EZH1	-1.17	1.60E-05	2.05E-04	0.45
ZNF837	-1.17	2.01E-02	7.47E-02	0.45
ANKZF1	-1.17	6.47E-04	4.78E-03	0.44
LRP5L	-1.17	8.81E-03	4.00E-02	0.44
RNF166	-1.17	3.66E-05	4.21E-04	0.44
SORL1	-1.17	1.92E-07	3.87E-06	0.44
LDHD	-1.17	3.84E-03	2.05E-02	0.44
CDCA7L	-1.17	9.62E-04	6.66E-03	0.44
MLPH	-1.17	1.80E-03	1.12E-02	0.44
SPSB2	-1.17	4.43E-05	5.00E-04	0.44
SLC16A13	-1.17	6.21E-03	3.00E-02	0.44
LINC01410	-1.18	3.60E-03	1.96E-02	0.44
BBS2	-1.18	1.36E-06	2.27E-05	0.44
SORBS3	-1.18	4.34E-08	9.94E-07	0.44
BTBD2	-1.18	4.76E-08	1.08E-06	0.44
THRA	-1.18	9.43E-06	1.29E-04	0.44
ASGR1	-1.18	1.18E-05	1.57E-04	0.44
ZNF704	-1.18	1.86E-04	1.70E-03	0.44
CBX7	-1.18	1.84E-04	1.69E-03	0.44
MUM1	-1.18	6.72E-06	9.45E-05	0.44
MAST3	-1.18	1.86E-07	3.77E-06	0.44
TMEM187	-1.18	2.96E-04	2.52E-03	0.44

RIN1	-1.19	1.11E-04	1.09E-03	0.44
IFT172	-1.19	1.82E-04	1.68E-03	0.44
GPR162	-1.19	2.04E-02	7.56E-02	0.44
SEC31B	-1.19	4.29E-03	2.25E-02	0.44
HPS4	-1.19	7.42E-09	2.06E-07	0.44
PLCL2	-1.19	6.31E-07	1.13E-05	0.44
TEF	-1.19	3.86E-06	5.81E-05	0.44
ENKD1	-1.19	1.16E-03	7.76E-03	0.44
TXNRD3	-1.19	2.44E-03	1.43E-02	0.44
MZF1	-1.19	3.53E-04	2.90E-03	0.44
ZNF232	-1.19	1.92E-04	1.75E-03	0.44
CROCC	-1.19	3.09E-05	3.62E-04	0.44
OCEL1	-1.19	3.65E-05	4.20E-04	0.44
TDRKH	-1.19	4.05E-05	4.61E-04	0.44
ITSN1	-1.20	4.52E-06	6.68E-05	0.44
C12orf76	-1.20	1.88E-04	1.72E-03	0.44
FDXR	-1.20	1.74E-03	1.09E-02	0.44
HLTF	-1.20	2.53E-04	2.20E-03	0.44
NREP	-1.20	1.10E-06	1.87E-05	0.44
ZNF710	-1.20	5.03E-09	1.45E-07	0.43
NCF4	-1.20	2.33E-09	7.24E-08	0.43
TGFBR2	-1.20	2.22E-09	6.92E-08	0.43
LTA4H	-1.21	3.97E-04	3.20E-03	0.43
ZC3H6	-1.21	5.10E-05	5.69E-04	0.43
NPRL2	-1.21	1.65E-04	1.54E-03	0.43
SMARCA2	-1.21	1.30E-09	4.27E-08	0.43
PADI2	-1.21	2.63E-04	2.27E-03	0.43
THAP8	-1.21	3.28E-04	2.74E-03	0.43
ORAI3	-1.21	1.34E-05	1.76E-04	0.43
CARD14	-1.21	9.31E-05	9.46E-04	0.43
PELI2	-1.21	4.41E-05	4.99E-04	0.43
ST8SIA4	-1.21	4.18E-09	1.22E-07	0.43
TCP11L2	-1.21	8.32E-04	5.92E-03	0.43
CATSPER1	-1.22	1.78E-03	1.11E-02	0.43
RAD51AP1	-1.22	1.50E-03	9.63E-03	0.43
PGPEP1	-1.22	1.10E-07	2.32E-06	0.43

SIPA1L3	-1.22	1.73E-05	2.21E-04	0.43
CORO1A	-1.22	5.45E-06	7.90E-05	0.43
MAP2K5	-1.22	3.06E-06	4.71E-05	0.43
INPP4A	-1.22	3.27E-09	9.94E-08	0.43
TBC1D24	-1.22	4.93E-07	9.08E-06	0.43
SLC48A1	-1.22	2.06E-04	1.87E-03	0.43
LXN	-1.22	1.33E-08	3.50E-07	0.43
HHEX	-1.23	1.02E-05	1.39E-04	0.43
KCNE3	-1.23	2.79E-06	4.34E-05	0.43
SLC24A4	-1.23	5.34E-05	5.89E-04	0.43
ERBB2	-1.23	1.42E-02	5.78E-02	0.43
RFX3	-1.23	6.81E-03	3.23E-02	0.43
SGSH	-1.24	2.77E-11	1.27E-09	0.42
ANG	-1.24	2.25E-03	1.34E-02	0.42
MGC16275	-1.24	9.04E-03	4.08E-02	0.42
MYCL	-1.24	2.70E-03	1.56E-02	0.42
KLC4	-1.24	4.50E-05	5.07E-04	0.42
AVPI1	-1.24	1.64E-03	1.03E-02	0.42
RTN4R	-1.24	2.26E-05	2.78E-04	0.42
PREX1	-1.24	1.23E-06	2.06E-05	0.42
LYL1	-1.24	5.32E-05	5.88E-04	0.42
LINC00942	-1.24	1.72E-02	6.65E-02	0.42
SUV420H2	-1.24	5.12E-04	3.92E-03	0.42
RASGRP3	-1.24	5.04E-05	5.63E-04	0.42
TNRC6B	-1.24	1.55E-08	4.02E-07	0.42
RPARP-AS1	-1.24	2.81E-03	1.60E-02	0.42
L3MBTL3	-1.25	1.84E-05	2.32E-04	0.42
XYLT2	-1.25	4.91E-05	5.50E-04	0.42
GTPBP3	-1.25	1.47E-04	1.39E-03	0.42
TREML1	-1.25	3.15E-03	1.76E-02	0.42
PPP1R21	-1.25	1.43E-06	2.37E-05	0.42
HERC2P9	-1.25	2.07E-02	7.65E-02	0.42
PPOX	-1.25	1.35E-03	8.88E-03	0.42
TRIM32	-1.25	6.82E-07	1.21E-05	0.42
CNRIP1	-1.25	1.49E-03	9.62E-03	0.42
CNNM3	-1.25	2.24E-07	4.43E-06	0.42

SLCO2B1	-1.26	1.74E-08	4.43E-07	0.42
RPS6KA4	-1.26	1.23E-09	4.09E-08	0.42
ZNF358	-1.26	2.82E-06	4.37E-05	0.42
ARHGAP27	-1.26	4.29E-07	7.99E-06	0.42
MEF2C	-1.26	1.82E-05	2.30E-04	0.42
C7orf43	-1.26	2.41E-09	7.46E-08	0.42
IGFBP4	-1.26	2.92E-05	3.46E-04	0.42
LINC00921	-1.26	1.43E-03	9.25E-03	0.42
FUZ	-1.27	3.07E-04	2.59E-03	0.42
WAS	-1.27	1.39E-10	5.53E-09	0.42
LDB1	-1.27	5.14E-10	1.88E-08	0.42
NUPR1	-1.27	3.74E-09	1.11E-07	0.42
SMPD3	-1.27	1.51E-02	6.05E-02	0.42
NOXA1	-1.27	1.12E-03	7.56E-03	0.41
IL21R	-1.27	2.37E-04	2.09E-03	0.41
A2M-AS1	-1.27	2.14E-02	7.86E-02	0.41
KLHDC3	-1.27	2.55E-09	7.85E-08	0.41
PRX	-1.27	3.51E-03	1.92E-02	0.41
SEMA6B	-1.27	4.14E-05	4.71E-04	0.41
CCDC85B	-1.28	4.43E-06	6.57E-05	0.41
GSDMA	-1.28	4.52E-03	2.34E-02	0.41
FNDC5	-1.28	1.41E-02	5.76E-02	0.41
OSBPL7	-1.28	2.45E-04	2.15E-03	0.41
SSH2	-1.28	5.23E-09	1.50E-07	0.41
SLC1A5	-1.28	7.45E-06	1.03E-04	0.41
NUDT7	-1.28	9.95E-04	6.84E-03	0.41
ZNF503	-1.28	2.36E-05	2.88E-04	0.41
LHX4-AS1	-1.28	1.53E-02	6.09E-02	0.41
ORMDL3	-1.28	4.55E-08	1.04E-06	0.41
LINC00265	-1.29	4.10E-03	2.16E-02	0.41
CCDC125	-1.29	6.90E-05	7.29E-04	0.41
ZNF433	-1.29	1.49E-02	5.98E-02	0.41
AP2A2	-1.29	1.25E-09	4.16E-08	0.41
LOC146880	-1.29	2.47E-07	4.82E-06	0.41
HMHA1	-1.29	2.64E-07	5.10E-06	0.41
SAP25	-1.30	1.74E-03	1.09E-02	0.41

EHD3	-1.30	2.41E-02	8.61E-02	0.41
ANKRD44	-1.30	7.89E-07	1.39E-05	0.41
MKNK2	-1.30	9.75E-08	2.09E-06	0.41
HEXDC	-1.30	1.59E-05	2.04E-04	0.41
TMEM143	-1.30	1.72E-03	1.08E-02	0.41
CLMN	-1.30	5.84E-08	1.31E-06	0.40
RILPL1	-1.31	4.49E-06	6.65E-05	0.40
SPHK2	-1.31	2.58E-08	6.24E-07	0.40
NECAB3	-1.31	1.03E-03	7.04E-03	0.40
WBSCR27	-1.31	9.08E-03	4.10E-02	0.40
CAPS	-1.31	6.82E-04	5.01E-03	0.40
POLB	-1.31	6.63E-05	7.04E-04	0.40
FAM216A	-1.32	1.12E-02	4.82E-02	0.40
PRR12	-1.32	3.69E-08	8.59E-07	0.40
NCKAP5L	-1.32	4.42E-09	1.29E-07	0.40
RDH12	-1.32	1.61E-02	6.34E-02	0.40
SNX18	-1.32	7.44E-11	3.12E-09	0.40
SMYD4	-1.32	1.59E-08	4.10E-07	0.40
INSR	-1.32	1.75E-07	3.54E-06	0.40
PCMTD1	-1.32	1.02E-07	2.18E-06	0.40
HAUS4	-1.32	3.19E-05	3.71E-04	0.40
FLJ20021	-1.32	1.51E-03	9.71E-03	0.40
SPTBN1	-1.33	2.73E-06	4.25E-05	0.40
LOC100132356	-1.33	1.00E-02	4.43E-02	0.40
KANK2	-1.33	3.44E-04	2.84E-03	0.40
KSR1	-1.33	1.56E-02	6.18E-02	0.40
C19orf68	-1.33	1.16E-03	7.76E-03	0.40
PFKFB2	-1.34	1.95E-07	3.92E-06	0.40
SNX21	-1.34	1.81E-04	1.67E-03	0.40
LOC728392	-1.34	2.20E-02	8.04E-02	0.39
SPATA12	-1.34	3.65E-03	1.97E-02	0.39
C14orf159	-1.34	8.95E-06	1.23E-04	0.39
P2RY1	-1.34	2.54E-02	8.97E-02	0.39
TRERF1	-1.34	1.30E-05	1.71E-04	0.39
POLD1	-1.35	3.63E-07	6.90E-06	0.39
AKNA	-1.35	2.23E-08	5.47E-07	0.39

RNASEH2B	-1.35	2.37E-07	4.66E-06	0.39
TMEM180	-1.35	2.22E-10	8.54E-09	0.39
TACC3	-1.35	2.85E-06	4.42E-05	0.39
KBTBD7	-1.35	2.02E-04	1.84E-03	0.39
CNNM2	-1.35	8.73E-05	8.94E-04	0.39
CHTF18	-1.35	1.02E-02	4.48E-02	0.39
LPPR3	-1.35	9.14E-04	6.39E-03	0.39
SYTL3	-1.35	3.09E-05	3.62E-04	0.39
PRIM1	-1.35	1.26E-02	5.26E-02	0.39
TNK2	-1.36	9.19E-10	3.18E-08	0.39
SLC29A1	-1.36	6.09E-10	2.16E-08	0.39
GINS1	-1.37	6.30E-04	4.69E-03	0.39
LINC01504	-1.37	1.21E-03	8.01E-03	0.39
MPST	-1.37	9.41E-09	2.58E-07	0.39
KIZ	-1.37	9.50E-04	6.60E-03	0.39
FYB	-1.38	9.10E-07	1.58E-05	0.38
ZNF788	-1.38	1.75E-03	1.10E-02	0.38
ZNF792	-1.38	1.95E-03	1.19E-02	0.38
SLC36A1	-1.38	4.53E-05	5.10E-04	0.38
MIF4GD	-1.38	1.74E-09	5.60E-08	0.38
LIN7B	-1.39	2.01E-02	7.50E-02	0.38
HMGB2	-1.39	1.11E-08	3.00E-07	0.38
EVA1B	-1.39	2.94E-10	1.12E-08	0.38
HEXIM2	-1.39	4.19E-04	3.34E-03	0.38
ARHGAP9	-1.39	4.53E-08	1.04E-06	0.38
PHF21A	-1.39	8.41E-10	2.92E-08	0.38
ING4	-1.40	1.16E-08	3.11E-07	0.38
CYP2U1	-1.40	4.61E-03	2.37E-02	0.38
ARL4C	-1.40	5.74E-09	1.63E-07	0.38
VSIG10	-1.40	8.58E-05	8.81E-04	0.38
PROCA1	-1.40	1.64E-02	6.42E-02	0.38
MFSD3	-1.40	1.35E-04	1.30E-03	0.38
ARPIN	-1.40	2.30E-07	4.54E-06	0.38
NFIA	-1.40	1.81E-03	1.12E-02	0.38
TMEM129	-1.40	8.27E-10	2.89E-08	0.38
ABCG1	-1.40	5.79E-06	8.31E-05	0.38

FAM117B	-1.41	1.34E-04	1.30E-03	0.38
SNX30	-1.41	2.48E-10	9.49E-09	0.38
PPAPDC2	-1.41	6.74E-05	7.15E-04	0.38
B3GALNT1	-1.42	1.69E-02	6.56E-02	0.37
C15orf62	-1.42	2.09E-02	7.72E-02	0.37
ZHX3	-1.42	1.16E-05	1.55E-04	0.37
ACACB	-1.42	2.63E-05	3.15E-04	0.37
KIAA1147	-1.42	3.19E-07	6.13E-06	0.37
HTRA1	-1.42	9.25E-03	4.15E-02	0.37
AKR1C3	-1.42	3.66E-04	2.98E-03	0.37
HGF	-1.43	1.46E-06	2.42E-05	0.37
CIITA	-1.43	5.52E-07	1.00E-05	0.37
ZNF331	-1.43	2.19E-08	5.39E-07	0.37
ZBTB4	-1.43	6.52E-13	4.04E-11	0.37
TTLL1	-1.43	4.42E-03	2.30E-02	0.37
LOC441081	-1.43	1.05E-09	3.58E-08	0.37
ZFP36L2	-1.44	3.16E-08	7.47E-07	0.37
FBXL20	-1.44	2.62E-11	1.21E-09	0.37
TK2	-1.44	1.83E-08	4.62E-07	0.37
MARVELD1	-1.44	1.78E-09	5.71E-08	0.37
DNASE1	-1.44	2.33E-02	8.37E-02	0.37
ZNF688	-1.44	2.33E-05	2.84E-04	0.37
PYROXD2	-1.45	2.81E-02	9.67E-02	0.37
RAP2B	-1.45	1.28E-13	8.98E-12	0.37
JCHAIN	-1.45	2.92E-02	9.93E-02	0.37
RELL2	-1.45	4.11E-03	2.17E-02	0.36
BRD3	-1.46	8.38E-07	1.47E-05	0.36
CEBPA	-1.46	6.86E-12	3.59E-10	0.36
ELMSAN1	-1.46	1.13E-14	9.28E-13	0.36
SMA5	-1.46	5.19E-05	5.76E-04	0.36
DCANP1	-1.46	1.67E-04	1.56E-03	0.36
ZNF362	-1.46	1.31E-11	6.48E-10	0.36
SPATA7	-1.46	1.38E-05	1.81E-04	0.36
SSH3	-1.46	3.25E-08	7.66E-07	0.36
SHMT1	-1.46	8.05E-05	8.32E-04	0.36
SPATC1L	-1.46	1.42E-04	1.35E-03	0.36

NT5DC2	-1.46	5.28E-07	9.65E-06	0.36
CLHC1	-1.46	2.22E-02	8.08E-02	0.36
SLC35E2B	-1.47	1.92E-07	3.87E-06	0.36
MAF	-1.47	1.86E-11	8.84E-10	0.36
SLC7A8	-1.47	5.83E-06	8.35E-05	0.36
KLHL24	-1.47	2.61E-11	1.21E-09	0.36
LOC100272216	-1.47	6.05E-03	2.95E-02	0.36
MRO	-1.48	6.20E-03	3.00E-02	0.36
TESK2	-1.48	2.14E-07	4.24E-06	0.36
RCBTB2	-1.48	5.41E-09	1.55E-07	0.36
ASIP	-1.48	1.42E-02	5.77E-02	0.36
FBXO36	-1.49	4.16E-03	2.19E-02	0.36
TSPAN15	-1.49	4.80E-04	3.70E-03	0.36
FAT2	-1.49	1.72E-03	1.08E-02	0.36
SRP14-AS1	-1.49	2.81E-03	1.61E-02	0.36
FLVCR1-AS1	-1.49	6.47E-03	3.10E-02	0.36
RERE	-1.50	2.07E-12	1.18E-10	0.35
LOC101927070	-1.50	1.81E-03	1.12E-02	0.35
ZBED3	-1.50	1.06E-05	1.43E-04	0.35
STARD13	-1.50	2.59E-08	6.26E-07	0.35
YPEL2	-1.50	1.99E-08	4.97E-07	0.35
SARM1	-1.51	5.88E-03	2.89E-02	0.35
KAT2A	-1.51	1.04E-06	1.78E-05	0.35
FAM13A	-1.51	7.80E-05	8.11E-04	0.35
OXER1	-1.51	6.09E-05	6.58E-04	0.35
SUOX	-1.52	1.75E-08	4.46E-07	0.35
MXD4	-1.52	8.09E-15	6.76E-13	0.35
LINC01094	-1.52	5.59E-07	1.01E-05	0.35
PLEKHA7	-1.52	4.37E-03	2.28E-02	0.35
SLC45A3	-1.52	6.36E-06	9.02E-05	0.35
IRS2	-1.53	2.10E-07	4.18E-06	0.35
NISCH	-1.53	2.77E-13	1.84E-11	0.35
CLEC10A	-1.53	4.07E-07	7.63E-06	0.35
LOC100288152	-1.54	1.41E-02	5.75E-02	0.35
SELPLG	-1.54	8.45E-11	3.51E-09	0.34
MFNG	-1.54	9.61E-12	4.90E-10	0.34

GLI4	-1.54	3.17E-05	3.70E-04	0.34
PSCA	-1.54	6.84E-03	3.24E-02	0.34
SDCCAG3	-1.55	4.31E-11	1.91E-09	0.34
SLC25A35	-1.55	6.86E-04	5.02E-03	0.34
NBPF1	-1.55	8.48E-06	1.17E-04	0.34
ARRDC2	-1.55	2.45E-08	5.96E-07	0.34
TRPV1	-1.55	3.83E-05	4.38E-04	0.34
EEPD1	-1.56	2.82E-08	6.77E-07	0.34
ZCCHC24	-1.56	1.91E-10	7.47E-09	0.34
ABHD15	-1.56	1.17E-08	3.14E-07	0.34
GPAT2	-1.56	2.43E-02	8.67E-02	0.34
EPOR	-1.56	1.15E-05	1.53E-04	0.34
RAB11FIP1	-1.56	1.24E-09	4.11E-08	0.34
ZNF519	-1.56	2.76E-02	9.54E-02	0.34
CREBRF	-1.56	5.88E-08	1.31E-06	0.34
SOWAHD	-1.56	6.64E-06	9.35E-05	0.34
GGA2	-1.56	7.92E-12	4.10E-10	0.34
MIS18BP1	-1.57	2.05E-07	4.10E-06	0.34
SAC3D1	-1.57	2.04E-04	1.85E-03	0.34
SUZ12P1	-1.57	1.63E-02	6.39E-02	0.34
LTC4S	-1.57	4.61E-04	3.59E-03	0.34
KBTBD3	-1.57	1.90E-03	1.17E-02	0.34
M1AP	-1.57	3.17E-04	2.66E-03	0.34
PITHD1	-1.58	1.64E-09	5.32E-08	0.33
FES	-1.59	2.57E-11	1.19E-09	0.33
IQSEC2	-1.59	2.38E-12	1.34E-10	0.33
ATG16L2	-1.59	7.80E-12	4.06E-10	0.33
DHRS3	-1.59	8.97E-09	2.47E-07	0.33
EDA	-1.59	1.93E-02	7.27E-02	0.33
ARMC7	-1.59	5.30E-12	2.82E-10	0.33
PIPOX	-1.59	2.81E-02	9.66E-02	0.33
KIAA0513	-1.59	1.08E-17	1.46E-15	0.33
MPO	-1.59	1.53E-04	1.45E-03	0.33
SLC40A1	-1.60	9.48E-10	3.27E-08	0.33
NR1D1	-1.60	3.28E-05	3.81E-04	0.33
FAM212B	-1.60	1.17E-09	3.95E-08	0.33

FOXRED2	-1.60	2.99E-11	1.36E-09	0.33
WDR91	-1.60	4.96E-16	5.01E-14	0.33
CEBPD	-1.60	5.37E-05	5.91E-04	0.33
TSHZ1	-1.60	1.34E-10	5.40E-09	0.33
LRRK2	-1.61	3.52E-09	1.07E-07	0.33
ZBTB12	-1.61	4.47E-03	2.32E-02	0.33
NAIP	-1.62	7.34E-09	2.04E-07	0.33
TBC1D14	-1.62	6.98E-11	2.94E-09	0.33
ZNF815P	-1.62	8.50E-03	3.90E-02	0.33
APBA1	-1.62	1.73E-14	1.38E-12	0.32
ULK1	-1.63	2.56E-08	6.21E-07	0.32
MAP3K12	-1.63	1.23E-08	3.29E-07	0.32
ABHD8	-1.63	3.08E-08	7.30E-07	0.32
BOLA1	-1.64	7.92E-04	5.69E-03	0.32
ARHGAP6	-1.64	2.97E-05	3.51E-04	0.32
KRBA1	-1.64	6.13E-04	4.58E-03	0.32
FAM105A	-1.64	3.48E-14	2.61E-12	0.32
GREB1	-1.65	7.71E-03	3.59E-02	0.32
P2RY6	-1.65	8.62E-05	8.84E-04	0.32
OSGEPL1	-1.65	3.45E-04	2.85E-03	0.32
MBP	-1.65	2.38E-13	1.61E-11	0.32
PAQR8	-1.66	1.67E-10	6.54E-09	0.32
BRSK1	-1.66	1.31E-02	5.42E-02	0.32
PEX11G	-1.66	1.95E-04	1.78E-03	0.32
HDAC5	-1.66	4.16E-11	1.85E-09	0.32
ADCK1	-1.66	6.33E-08	1.40E-06	0.32
MMP15	-1.66	1.46E-02	5.89E-02	0.32
RAB42	-1.67	1.31E-08	3.46E-07	0.31
ZNF835	-1.67	6.66E-03	3.18E-02	0.31
NATD1	-1.67	2.96E-08	7.06E-07	0.31
HS3ST2	-1.67	1.68E-12	9.66E-11	0.31
SMAD6	-1.68	7.92E-08	1.73E-06	0.31
IFFO1	-1.68	1.79E-17	2.24E-15	0.31
SLC22A18AS	-1.68	2.39E-04	2.11E-03	0.31
BLNK	-1.69	6.17E-11	2.64E-09	0.31
UNC5B	-1.69	3.34E-06	5.08E-05	0.31

WIPF3	-1.69	1.55E-02	6.14E-02	0.31
FGD3	-1.70	6.91E-09	1.92E-07	0.31
LINC00910	-1.70	7.67E-03	3.58E-02	0.31
FUT7	-1.70	1.24E-02	5.22E-02	0.31
PIK3C2B	-1.71	1.45E-10	5.75E-09	0.31
FAXDC2	-1.71	4.69E-04	3.64E-03	0.31
DIRC2	-1.71	4.92E-11	2.15E-09	0.31
DYRK1B	-1.71	1.06E-09	3.61E-08	0.31
INPP5D	-1.72	1.31E-16	1.42E-14	0.30
FBXL8	-1.72	2.87E-03	1.63E-02	0.30
ZMYM3	-1.72	5.24E-13	3.30E-11	0.30
PAQR7	-1.72	1.63E-07	3.33E-06	0.30
LRMP	-1.72	1.54E-05	1.98E-04	0.30
ENDOG	-1.72	3.33E-04	2.77E-03	0.30
EPHB2	-1.72	1.16E-16	1.27E-14	0.30
RGS12	-1.72	2.76E-11	1.27E-09	0.30
ANXA9	-1.73	1.02E-03	7.00E-03	0.30
SH3PXD2A	-1.74	2.57E-07	4.99E-06	0.30
RAB3D	-1.74	3.92E-09	1.16E-07	0.30
ANGPTL2	-1.74	3.64E-03	1.97E-02	0.30
ID3	-1.74	1.26E-11	6.29E-10	0.30
GRAMD1B	-1.75	1.63E-07	3.33E-06	0.30
RASGRP4	-1.75	5.02E-14	3.72E-12	0.30
BBC3	-1.75	4.16E-06	6.22E-05	0.30
PLEKHH3	-1.75	3.16E-03	1.76E-02	0.30
WDR81	-1.75	1.62E-15	1.49E-13	0.30
TFCP2L1	-1.75	1.67E-05	2.13E-04	0.30
RAP1GAP2	-1.76	8.44E-08	1.83E-06	0.30
TMIGD3	-1.76	7.81E-05	8.11E-04	0.30
CARD9	-1.76	6.15E-09	1.74E-07	0.30
RASL10A	-1.76	5.73E-03	2.83E-02	0.30
REEP4	-1.77	3.46E-13	2.26E-11	0.29
ZNF589	-1.77	1.27E-08	3.36E-07	0.29
LOC100506585	-1.77	1.61E-11	7.84E-10	0.29
HTR7	-1.78	4.14E-06	6.18E-05	0.29
EHMT2	-1.78	1.93E-16	2.06E-14	0.29

CCDC170	-1.78	9.52E-04	6.61E-03	0.29
ENC1	-1.78	6.55E-08	1.44E-06	0.29
RPP25	-1.79	6.62E-11	2.81E-09	0.29
MBNL2	-1.79	1.27E-08	3.36E-07	0.29
C16orf86	-1.79	3.27E-04	2.73E-03	0.29
GRAMD4	-1.80	6.84E-21	1.30E-18	0.29
FFAR4	-1.80	2.31E-03	1.36E-02	0.29
LINC00174	-1.80	4.26E-04	3.38E-03	0.29
TYSND1	-1.80	1.28E-10	5.20E-09	0.29
PANK1	-1.81	5.17E-03	2.60E-02	0.29
ARRDC5	-1.82	1.13E-02	4.85E-02	0.28
GATS	-1.82	1.83E-05	2.31E-04	0.28
RCN3	-1.83	9.68E-08	2.08E-06	0.28
PRAM1	-1.83	1.45E-10	5.75E-09	0.28
RHOBTB1	-1.83	5.97E-11	2.56E-09	0.28
CALCRL	-1.83	4.71E-04	3.65E-03	0.28
CRHBP	-1.84	8.30E-04	5.91E-03	0.28
CLCN4	-1.84	3.23E-07	6.20E-06	0.28
LRRC45	-1.85	3.15E-05	3.68E-04	0.28
CREB3L4	-1.85	2.63E-03	1.52E-02	0.28
KHK	-1.85	4.52E-05	5.09E-04	0.28
ATG4C	-1.85	1.95E-09	6.16E-08	0.28
MERTK	-1.86	5.62E-07	1.02E-05	0.28
RGS14	-1.86	5.58E-05	6.09E-04	0.28
LINC00324	-1.86	4.93E-04	3.79E-03	0.28
RNF44	-1.86	2.08E-15	1.89E-13	0.27
ZNF763	-1.88	2.99E-04	2.54E-03	0.27
THEM6	-1.88	1.92E-08	4.84E-07	0.27
PDK2	-1.89	1.90E-08	4.80E-07	0.27
ABCC5	-1.89	8.33E-11	3.47E-09	0.27
LOC100129550	-1.89	1.91E-09	6.05E-08	0.27
TLR3	-1.89	9.06E-04	6.35E-03	0.27
PRR34	-1.91	8.86E-03	4.02E-02	0.27
SETDB2	-1.92	3.20E-16	3.31E-14	0.26
HVCN1	-1.92	1.31E-10	5.31E-09	0.26
SLC16A5	-1.93	2.77E-15	2.47E-13	0.26

SUFU	-1.93	2.43E-15	2.19E-13	0.26
KLF1	-1.93	3.64E-03	1.97E-02	0.26
SOX12	-1.94	3.53E-05	4.08E-04	0.26
NLRP12	-1.94	5.84E-08	1.31E-06	0.26
CYGB	-1.95	1.11E-06	1.89E-05	0.26
RASAL1	-1.95	5.57E-11	2.40E-09	0.26
CCNG2	-1.95	4.88E-17	5.66E-15	0.26
PCED1B	-1.95	1.17E-08	3.14E-07	0.26
FRAT1	-1.96	1.25E-04	1.22E-03	0.26
C9orf139	-1.96	1.18E-02	4.98E-02	0.26
SLC27A1	-1.97	1.78E-22	4.04E-20	0.26
KIAA1107	-1.97	2.20E-03	1.32E-02	0.26
GPR34	-1.97	1.44E-07	2.96E-06	0.25
CELF6	-1.98	6.30E-09	1.77E-07	0.25
ST5	-1.98	1.17E-05	1.56E-04	0.25
SCAMP5	-1.99	2.42E-07	4.74E-06	0.25
FBXO32	-1.99	3.14E-06	4.80E-05	0.25
C1orf127	-1.99	2.20E-06	3.48E-05	0.25
B4GAT1	-2.00	5.42E-07	9.89E-06	0.25
LPAR6	-2.01	1.27E-12	7.41E-11	0.25
LOC102723701	-2.01	1.78E-03	1.11E-02	0.25
LOC153684	-2.01	5.25E-06	7.63E-05	0.25
SH2D3C	-2.02	1.13E-09	3.79E-08	0.25
EYA2	-2.02	1.15E-02	4.90E-02	0.25
ADCK3	-2.02	8.72E-16	8.53E-14	0.25
TLR7	-2.02	1.29E-06	2.16E-05	0.25
AP3S2	-2.03	1.66E-02	6.47E-02	0.25
ACSS1	-2.03	3.37E-10	1.27E-08	0.24
DAB2IP	-2.08	4.65E-04	3.61E-03	0.24
MGAT4A	-2.08	3.82E-23	9.57E-21	0.24
SCARF2	-2.08	1.31E-02	5.41E-02	0.24
LINC00865	-2.10	6.37E-03	3.06E-02	0.23
LPAR5	-2.12	1.57E-12	9.11E-11	0.23
TRPS1	-2.12	4.84E-10	1.78E-08	0.23
FLI1	-2.13	2.54E-16	2.69E-14	0.23
JMY	-2.13	1.01E-11	5.07E-10	0.23

CACNA2D3	-2.14	1.77E-06	2.89E-05	0.23
TTC7A	-2.16	5.86E-23	1.41E-20	0.22
MUC6	-2.17	3.54E-03	1.93E-02	0.22
FOS	-2.17	2.03E-02	7.53E-02	0.22
FAM53B	-2.19	2.92E-20	4.88E-18	0.22
RNF125	-2.19	1.07E-13	7.55E-12	0.22
PPAPDC3	-2.20	9.42E-04	6.55E-03	0.22
S1PR1	-2.21	1.55E-13	1.07E-11	0.22
CHST13	-2.22	5.07E-19	7.66E-17	0.21
KCNMB4	-2.22	2.27E-06	3.58E-05	0.21
LRRC4	-2.24	1.12E-06	1.91E-05	0.21
TLR5	-2.25	1.29E-21	2.52E-19	0.21
APPL2	-2.26	7.44E-27	2.59E-24	0.21
HSPBAP1	-2.27	1.25E-15	1.18E-13	0.21
CEBPA-AS1	-2.27	1.53E-06	2.53E-05	0.21
CEMP1	-2.28	1.34E-08	3.50E-07	0.21
RPS6KA5	-2.28	7.47E-06	1.04E-04	0.21
YPEL4	-2.29	2.46E-12	1.38E-10	0.20
CELSR2	-2.30	2.01E-05	2.51E-04	0.20
NCK1-AS1	-2.31	4.33E-08	9.93E-07	0.20
STOX2	-2.32	1.10E-02	4.74E-02	0.20
PALD1	-2.33	7.75E-05	8.07E-04	0.20
GPR150	-2.34	5.46E-04	4.15E-03	0.20
LGR4	-2.34	1.15E-07	2.42E-06	0.20
PTCH2	-2.35	7.27E-06	1.01E-04	0.20
RGS18	-2.35	7.06E-10	2.49E-08	0.20
SERPINF2	-2.37	1.43E-09	4.68E-08	0.19
PSRC1	-2.40	1.51E-03	9.71E-03	0.19
CD1D	-2.41	5.37E-05	5.91E-04	0.19
GAPT	-2.42	2.96E-08	7.06E-07	0.19
SEMA6C	-2.44	6.43E-04	4.76E-03	0.18
TMEM37	-2.48	1.09E-16	1.21E-14	0.18
ZNF467	-2.54	9.56E-16	9.29E-14	0.17
GPBAR1	-2.57	1.05E-07	2.25E-06	0.17
SLC46A2	-2.58	1.70E-07	3.47E-06	0.17
OPRL1	-2.59	3.29E-10	1.24E-08	0.17

TBX1	-2.60	2.12E-04	1.91E-03	0.17
FZD2	-2.61	3.73E-13	2.42E-11	0.16
PIK3IP1	-2.61	3.83E-20	6.31E-18	0.16
CABLES1	-2.64	8.03E-11	3.35E-09	0.16
KIAA1211L	-2.68	2.83E-11	1.29E-09	0.16
P2RY13	-2.70	4.88E-08	1.10E-06	0.15
LRP5	-2.70	5.32E-11	2.31E-09	0.15
SLC46A1	-2.72	2.52E-14	1.96E-12	0.15
C15orf52	-2.77	1.63E-17	2.07E-15	0.15
ALK	-2.79	2.16E-17	2.65E-15	0.15
LOC101929331	-2.81	6.91E-10	2.45E-08	0.14
CD200R1	-2.82	3.43E-10	1.29E-08	0.14
TRIM58	-2.83	1.32E-09	4.35E-08	0.14
YPEL3	-2.87	5.22E-17	5.95E-15	0.14
F2RL3	-2.89	3.75E-04	3.06E-03	0.14
TMEM86A	-2.94	6.41E-26	2.01E-23	0.13
GATSL2	-2.98	6.65E-12	3.53E-10	0.13
SLC45A4	-3.04	7.01E-38	5.16E-35	0.12
UCP3	-3.15	8.55E-05	8.79E-04	0.11
PDK4	-3.17	2.14E-39	1.79E-36	0.11
NYNRIN	-3.23	8.84E-04	6.22E-03	0.11
C19orf35	-3.24	1.10E-10	4.54E-09	0.11
GPRC5B	-3.26	9.11E-13	5.54E-11	0.10
NHSL2	-3.26	1.26E-28	4.93E-26	0.10
DBP	-3.35	9.30E-28	3.33E-25	0.10
PROC	-3.42	4.55E-09	1.32E-07	0.09
DEPTOR	-3.43	4.91E-10	1.81E-08	0.09
BMF	-3.45	8.76E-21	1.64E-18	0.09
KLHDC8B	-3.46	5.45E-23	1.34E-20	0.09
IQCD	-3.63	2.20E-06	3.47E-05	0.08
ADORA3	-3.72	2.68E-26	8.61E-24	0.08
GAL3ST4	-3.83	5.82E-30	2.43E-27	0.07
LINGO3	-4.07	6.99E-16	6.90E-14	0.06
CTTNBP2	-4.22	1.68E-11	8.08E-10	0.05

Differentially expressed genes (padj < 0.1, log2FC ≤ - 1) in the anti-CCL2 Ab 4h vs nil 4h comparison (dataset 1)
