# Supplementary material for: Transcriptome Profiling of Human Monocyte-Derived Macrophages Upon CCL2 Neutralization Reveals an Association Between Activation of Innate Immune Pathways and Restriction of HIV-1 Gene Expression
Source: Front Immunol. 2020 Sep 18;11:2129. doi: 10.3389/fimmu.2020.02129 (PMC7531389; doi:10.3389/fimmu.2020.02129)
Supplement: Supplementary file 1 [file Data_Sheet_1.zip › Supplementary tables/Covino et al_Supplementary Table 6.pdf]

Supplementary Table 6. List of differentially expressed genes with padj ≤ 0.1 in HIV-1 versus nil at day 1 p.i. (dataset 3).

GENE	*log2FC*	*pvalue*	*padj*	*FC*
Upregulated				
NLRP12	1.52	4.81E-07	4.55E-04	2.86
PLA2G16	1.51	3.07E-04	5.01E-02	2.86
ANKRD22	1.48	1.46E-03	1.36E-01	2.78
GGT5	1.35	9.40E-08	2.14E-04	2.56
STAC	1.19	6.08E-05	1.69E-02	2.28
HSD11B1	1.09	5.45E-11	6.20E-07	2.13
CCL24	1.06	8.70E-07	6.60E-04	2.09
FPR2	1.02	1.39E-04	3.04E-02	2.03
C1RL	0.95	3.38E-05	1.13E-02	1.93
EXT1	0.94	2.32E-04	4.27E-02	1.92
CD300E	0.91	4.58E-05	1.38E-02	1.88
NCR3LG1	0.90	3.09E-04	5.01E-02	1.86
FPR1	0.84	2.07E-10	1.18E-06	1.79
VCAM1	0.83	8.34E-04	9.20E-02	1.77
CXCL11	0.83	3.24E-04	5.11E-02	1.77
MLLT4	0.70	2.01E-05	7.88E-03	1.62
P2RY13	0.69	2.17E-05	8.01E-03	1.61
SUCNR1	0.68	1.16E-04	2.70E-02	1.60
ALOX5AP	0.67	9.23E-05	2.38E-02	1.60
IL21R	0.66	2.83E-06	1.53E-03	1.59
IL7R	0.65	1.16E-06	7.78E-04	1.57
VCAN	0.64	1.39E-03	1.33E-01	1.56
CCL13	0.64	7.10E-04	8.40E-02	1.56
IDO1	0.63	7.84E-04	8.88E-02	1.55
FN1	0.63	2.46E-06	1.40E-03	1.55
APOL4	0.63	1.52E-03	1.38E-01	1.54
ARNTL2	0.61	5.04E-05	1.47E-02	1.52
ANKRD36BP1	0.60	1.55E-04	3.21E-02	1.52
MYOF	0.60	1.11E-06	7.78E-04	1.52
CXCL10	0.60	4.47E-08	1.27E-04	1.52

LAMB3	0.55	1.50E-04	3.16E-02	1.47
ENPP2	0.54	7.95E-04	8.88E-02	1.46
ALDH18A1	0.54	1.11E-03	1.16E-01	1.45
PRKAR2A	0.54	4.52E-04	6.27E-02	1.45
ACVRL1	0.53	1.19E-03	1.23E-01	1.45
SULF2	0.51	4.56E-05	1.38E-02	1.42
PTPRM	0.50	6.28E-04	7.60E-02	1.42
GBP4	0.49	2.98E-04	4.99E-02	1.41
SIGLEC10	0.49	4.55E-07	4.55E-04	1.41
OLR1	0.49	1.05E-05	4.41E-03	1.40
OAS3	0.49	7.20E-05	1.95E-02	1.40
QPCT	0.49	1.07E-04	2.57E-02	1.40
IL27RA	0.47	5.44E-04	6.98E-02	1.39
HIPK2	0.47	2.63E-04	4.60E-02	1.39
IL1B	0.47	1.79E-04	3.57E-02	1.39
ECE1	0.47	1.38E-03	1.33E-01	1.39
MT1G	0.46	2.19E-05	8.01E-03	1.37
ITGB7	0.46	1.57E-03	1.40E-01	1.37
SH3PXD2B	0.45	1.86E-04	3.65E-02	1.37
MT1F	0.44	4.49E-04	6.27E-02	1.36
ADGRE2	0.44	1.21E-03	1.24E-01	1.35
HIVEP3	0.44	2.23E-04	4.22E-02	1.35
MIR6087	0.44	5.52E-04	6.98E-02	1.35
NID1	0.43	1.37E-04	3.04E-02	1.35
CRIM1	0.43	5.84E-04	7.27E-02	1.34
SPP1	0.42	3.75E-04	5.58E-02	1.34
FASN	0.42	6.66E-06	3.15E-03	1.34
AGRN	0.40	7.17E-06	3.26E-03	1.32
SLC7A1	0.40	1.48E-03	1.36E-01	1.32
POLR1A	0.40	5.88E-04	7.27E-02	1.32
MRC2	0.40	5.51E-04	6.98E-02	1.32
MT2A	0.40	5.15E-06	2.54E-03	1.32
SPN	0.39	2.45E-04	4.36E-02	1.31
RTN1	0.39	8.78E-04	9.42E-02	1.31
GPR84	0.39	1.47E-04	3.15E-02	1.31

FAM129A	0.38	6.47E-04	7.74E-02	1.30
PFKFB2	0.38	7.63E-04	8.83E-02	1.30
GBP5	0.38	1.67E-03	1.45E-01	1.30
GBP1	0.38	1.08E-04	2.57E-02	1.30
ARHGAP31	0.37	1.62E-04	3.29E-02	1.30
FLVCR2	0.37	1.26E-03	1.26E-01	1.29
CCL5	0.37	7.95E-05	2.10E-02	1.29
BCOR	0.36	7.97E-04	8.88E-02	1.29
SERPINA1	0.36	4.60E-05	1.38E-02	1.28
ADAMTSL4	0.35	4.97E-04	6.54E-02	1.27
C15orf48	0.35	1.31E-04	2.99E-02	1.27
EHD1	0.34	9.86E-04	1.05E-01	1.26
SLC8A1	0.33	1.41E-03	1.34E-01	1.26
HCFC1	0.33	2.40E-04	4.34E-02	1.26
FNDC3B	0.33	1.23E-03	1.25E-01	1.26
ACE	0.32	3.31E-04	5.16E-02	1.25
SLC2A6	0.32	7.58E-04	8.83E-02	1.25
ARSB	0.32	7.69E-04	8.83E-02	1.25
CPD	0.32	1.70E-03	1.47E-01	1.25
IL4I1	0.32	2.33E-04	4.27E-02	1.24
DOCK8	0.31	1.66E-03	1.45E-01	1.24
MMP14	0.29	3.78E-04	5.58E-02	1.22
STAT1	0.28	4.41E-04	6.27E-02	1.22
ICAM1	0.28	1.65E-03	1.45E-01	1.21
Downregulated				
RCN3	-1.1	1.48E-06	9E-04	0.5
CCDC152	-1.0	1.29E-03	1E-01	0.5
CXCL12	-0.8	3.88E-07	4E-04	0.6
F13A1	-0.6	6.16E-07	5E-04	0.6
DHRS9	-0.6	5.36E-05	2E-02	0.7
LOC100129550	-0.6	9.53E-05	2E-02	0.7
RGS2	-0.6	4.15E-04	6E-02	0.7
EGR1	-0.6	2.15E-06	1E-03	0.7
RNASE1	-0.5	1.91E-08	7E-05	0.7
SEPP1	-0.5	8.64E-07	7E-04	0.7

SAT1	-0.5	2.24E-07	4E-04	0.7
HS3ST2	-0.5	1.35E-07	3E-04	0.7
YPEL3	-0.5	4.78E-04	6E-02	0.7
LGMN	-0.5	3.62E-07	4E-04	0.7
TMEM119	-0.5	1.57E-03	1E-01	0.7
CD163L1	-0.5	3.02E-07	4E-04	0.7
PDK4	-0.5	7.55E-06	3E-03	0.7
STAB1	-0.4	2.69E-05	1E-02	0.7
CEBPD	-0.4	4.15E-05	1E-02	0.7
STMN1	-0.4	1.99E-04	4E-02	0.7
GAS6	-0.4	4.44E-04	6E-02	0.7
SLC7A8	-0.4	3.36E-06	2E-03	0.7
FABP4	-0.4	2.93E-05	1E-02	0.7
MALAT1	-0.4	1.04E-03	1E-01	0.7
GSDMA	-0.4	1.27E-03	1E-01	0.8
NUPR1	-0.4	2.84E-04	5E-02	0.8
PIK3IP1	-0.4	1.49E-03	1E-01	0.8
SLC40A1	-0.4	3.54E-04	5E-02	0.8
IER2	-0.4	2.73E-04	5E-02	0.8
GPNMB	-0.3	1.39E-05	6E-03	0.8
RARRES1	-0.3	1.08E-04	3E-02	0.8
IFI30	-0.3	3.58E-04	5E-02	0.8
LILRB5	-0.3	3.14E-04	5E-02	0.8
BTG2	-0.3	5.01E-04	7E-02	0.8
FUCA1	-0.3	1.26E-03	1E-01	0.8
APPL2	-0.3	1.39E-03	1E-01	0.8
MPP1	-0.3	8.69E-04	9E-02	0.8
EVI2A	-0.3	1.44E-03	1E-01	0.8
MCOLN1	-0.3	8.76E-04	9E-02	0.8
AIF1	-0.3	4.79E-04	6E-02	0.8
ITM2B	-0.3	4.69E-04	6E-02	0.8
CTSZ	-0.3	5.95E-04	7E-02	0.8
CTSS	-0.2	1.56E-03	1E-01	0.8
