# Supplementary material for: Transcriptome Profiling of Human Monocyte-Derived Macrophages Upon CCL2 Neutralization Reveals an Association Between Activation of Innate Immune Pathways and Restriction of HIV-1 Gene Expression
Source: Front Immunol. 2020 Sep 18;11:2129. doi: 10.3389/fimmu.2020.02129 (PMC7531389; doi:10.3389/fimmu.2020.02129)
Supplement: Supplementary file 1 [file Data_Sheet_1.zip › Supplementary tables/Covino et al_Supplementary Table 7.pdf]

Supplementary Table 7. List of differentially expressed genes with padj ≤ 0.1 and FC ≥ 2 in HIV-1+anti-CCL2 Ab vs HIV at day 1 p.i. (dataset 3).

GENE	*log2FC*	*pvalue*	*padj*	*FC*
Upregulated				
CXCL13	4.35	1.46E-03	3.0E-02	20.42
CHI3L2	2.93	4.74E-26	3.1E-23	7.63
IFI44L	2.85	3.23E-11	6.3E-09	7.19
IFI27	2.25	1.52E-03	3.1E-02	4.77
PDPN	2.09	5.58E-05	2.4E-03	4.26
MX1	1.98	5.01E-48	2.6E-44	3.94
IFIT3	1.98	1.75E-73	1.8E-69	3.93
COL1A1	1.92	1.06E-08	1.6E-06	3.79
IFIT1	1.87	2.46E-44	8.6E-41	3.67
RSAD2	1.78	3.26E-39	4.9E-36	3.44
OASL	1.78	1.52E-21	7.3E-19	3.44
CCL8	1.72	7.91E-32	6.9E-29	3.29
XAF1	1.72	1.15E-38	1.5E-35	3.28
USP18	1.67	3.41E-23	1.8E-20	3.17
CCL7	1.65	2.71E-03	4.8E-02	3.14
OAS2	1.65	2.41E-39	4.2E-36	3.13
MX2	1.64	2.16E-27	1.5E-24	3.12
OAS3	1.64	7.56E-34	7.2E-31	3.11
IFI44	1.63	1.52E-34	1.6E-31	3.09
LY6E	1.60	4.08E-07	3.8E-05	3.03
IFITM3	1.58	2.69E-04	8.2E-03	2.98
ISG15	1.53	8.53E-35	9.9E-32	2.89
CMPK2	1.52	2.60E-28	1.9E-25	2.87
MT1H	1.50	3.92E-04	1.1E-02	2.84
IFIT2	1.50	9.25E-32	7.5E-29	2.83
XIST	1.49	2.37E-05	1.2E-03	2.82
IFI6	1.44	1.42E-05	8.0E-04	2.72
HERC6	1.43	1.62E-15	5.0E-13	2.69
MT1G	1.42	1.54E-04	5.2E-03	2.68
DDX60	1.41	8.09E-23	4.0E-20	2.65
TIMP1	1.35	8.36E-41	1.8E-37	2.56

EIF2AK2	1.35	3.77E-20	1.6E-17	2.55
HELZ2	1.35	2.05E-25	1.3E-22	2.54
MARCO	1.32	9.57E-03	1.2E-01	2.49
TMEM26	1.28	3.09E-11	6.2E-09	2.43
OAS1	1.26	1.48E-24	8.6E-22	2.40
KITLG	1.26	6.34E-06	4.1E-04	2.39
MT1M	1.24	4.60E-07	4.2E-05	2.36
EPSTI1	1.17	2.72E-04	8.2E-03	2.25
IRF7	1.16	8.17E-19	3.4E-16	2.24
S100A8	1.14	8.62E-18	3.2E-15	2.21
MT1F	1.14	2.13E-03	4.0E-02	2.20
ENC1	1.14	5.24E-11	9.8E-09	2.20
HERC5	1.13	9.66E-09	1.4E-06	2.19
CLEC5A	1.13	5.24E-03	7.5E-02	2.19
SAMD9	1.12	8.03E-17	2.7E-14	2.17
CCL2	1.11	2.44E-17	8.5E-15	2.16
SAMD9L	1.09	3.09E-20	1.4E-17	2.14
PARP9	1.08	2.17E-23	1.2E-20	2.11
STON2	1.04	5.93E-08	7.2E-06	2.05
VSIG4	1.02	3.42E-03	5.6E-02	2.03
SERPINB2	1.01	1.62E-03	3.2E-02	2.02
FCGR1A	1.00	4.92E-10	8.3E-08	2.00
CTSL	0.99	3.00E-18	1.2E-15	1.98
FFAR2	0.98	2.12E-05	1.1E-03	1.97
GAL3ST4	0.98	6.15E-13	1.5E-10	1.97
BATF2	0.97	2.97E-07	2.9E-05	1.96
RCN3	0.97	3.88E-05	1.8E-03	1.96
ATP10A	0.96	1.07E-05	6.2E-04	1.95
B3GNT7	0.96	7.34E-07	6.3E-05	1.95
DHRS9	0.96	8.88E-03	1.1E-01	1.95
Downregulated				
STEAP4	-2.40	1.47E-14	3.9E-12	0.19
VCAM1	-2.36	6.17E-15	1.7E-12	0.19
PTPRF	-2.09	2.41E-10	4.1E-08	0.23
HIC1	-1.78	1.61E-06	1.3E-04	0.29
TNFRSF4	-1.75	1.11E-07	1.2E-05	0.30

UBD	-1.66	4.16E-06	2.8E-04	0.32
GFRA2	-1.58	4.12E-15	1.2E-12	0.34
FCN1	-1.51	2.35E-43	6.2E-40	0.35
GP1BA	-1.46	1.95E-06	1.5E-04	0.36
PID1	-1.40	4.19E-07	3.8E-05	0.38
PALD1	-1.36	7.99E-16	2.5E-13	0.39
SLC2A6	-1.35	1.83E-17	6.6E-15	0.39
SLC35F2	-1.29	2.18E-07	2.2E-05	0.41
CD244	-1.28	7.53E-07	6.4E-05	0.41
FZD7	-1.26	4.55E-06	3.0E-04	0.42
VILL	-1.25	3.14E-06	2.3E-04	0.42
TNFRSF18	-1.14	8.53E-04	2.0E-02	0.45
DGAT2	-1.12	7.81E-05	3.1E-03	0.46
DCANP1	-1.11	9.75E-14	2.4E-11	0.46
CFP	-1.09	1.52E-11	3.2E-09	0.47
TIFAB	-1.08	3.68E-12	8.2E-10	0.47
NCF1B	-1.07	7.33E-08	8.6E-06	0.48
HSPG2	-1.07	5.67E-05	2.4E-03	0.48
COL9A2	-1.06	5.49E-04	1.4E-02	0.48
MMP15	-1.05	1.80E-04	5.9E-03	0.48
CHST2	-1.02	4.25E-06	2.9E-04	0.49
P2RY13	-1.00	5.28E-08	6.6E-06	0.50
MARCKSL1	-0.99	8.55E-11	1.5E-08	0.50
FUT7	-0.99	1.86E-04	6.0E-03	0.50
ANTXR1	-0.97	5.77E-03	8.0E-02	0.51
MYCL	-0.97	2.67E-07	2.6E-05	0.51
NCF1C	-0.96	3.70E-06	2.5E-04	0.51
