# Supplementary material for: Transcriptome Profiling of Human Monocyte-Derived Macrophages Upon CCL2 Neutralization Reveals an Association Between Activation of Innate Immune Pathways and Restriction of HIV-1 Gene Expression
Source: Front Immunol. 2020 Sep 18;11:2129. doi: 10.3389/fimmu.2020.02129 (PMC7531389; doi:10.3389/fimmu.2020.02129)
Supplement: Supplementary file 1 [file Data_Sheet_1.zip › Supplementary tables/Covino et al_Supplementary Table 8.pdf]

**Supplementary Table 8.** List of differentially expressed genes with padj ≤ 0.1 and FC ≥ 2 in HIV-1+anti-CCL2 Ab at day 4 p.i. (dataset 3).

| *GENE* | *log2FC* | *pvalue* | *padj* | *FC* |
|---|---|---|---|---|
| *Upregulated* | | | | |
| ANKRD22 | 3.50 | 1.26E-04 | 8.1E-03 | 11.32 |
| CCL8 | 3.08 | 2.86E-04 | 1.5E-02 | 8.43 |
| EDN1 | 2.81 | 1.58E-06 | 3.0E-04 | 7.00 |
| TREM1 | 2.74 | 1.13E-03 | 3.8E-02 | 6.67 |
| ZBED2 | 2.72 | 3.42E-07 | 8.6E-05 | 6.59 |
| PDPN | 2.60 | 2.36E-19 | 9.1E-16 | 6.07 |
| CASP5 | 2.54 | 3.55E-06 | 5.4E-04 | 5.81 |
| IFI27 | 2.50 | 3.66E-04 | 1.8E-02 | 5.67 |
| AIM2 | 2.45 | 9.03E-06 | 1.2E-03 | 5.45 |
| FFAR2 | 2.19 | 1.65E-10 | 9.5E-08 | 4.57 |
| VSIG4 | 2.14 | 2.87E-03 | 7.1E-02 | 4.40 |
| MARCO | 2.13 | 6.70E-04 | 2.6E-02 | 4.36 |
| TNIP3 | 2.09 | 1.06E-05 | 1.3E-03 | 4.25 |
| ITGB7 | 2.01 | 8.77E-13 | 1.1E-09 | 4.04 |
| CCL23 | 1.95 | 1.44E-05 | 1.7E-03 | 3.85 |
| IFI44L | 1.91 | 1.30E-03 | 4.1E-02 | 3.76 |
| ITLN1 | 1.80 | 8.78E-05 | 6.4E-03 | 3.49 |
| MGAM | 1.78 | 1.22E-03 | 3.9E-02 | 3.44 |
| MT1M | 1.77 | 6.85E-04 | 2.7E-02 | 3.42 |
| IL6 | 1.75 | 5.47E-04 | 2.3E-02 | 3.37 |
| CCL2 | 1.74 | 9.33E-12 | 8.3E-09 | 3.33 |
| OLR1 | 1.73 | 3.25E-11 | 2.3E-08 | 3.31 |
| MACC1 | 1.73 | 6.33E-05 | 5.1E-03 | 3.31 |
| IFIT3 | 1.70 | 3.55E-21 | 4.1E-17 | 3.25 |
| GAL3ST4 | 1.68 | 3.84E-08 | 1.1E-05 | 3.21 |
| APOBEC3A | 1.61 | 8.92E-07 | 1.9E-04 | 3.05 |
| CREB5 | 1.61 | 2.18E-05 | 2.3E-03 | 3.04 |
| TNFAIP6 | 1.60 | 3.18E-06 | 5.0E-04 | 3.03 |
| XAF1 | 1.56 | 5.82E-13 | 9.6E-10 | 2.96 |
| IL1A | 1.55 | 5.64E-03 | 1.1E-01 | 2.92 |
| RSAD2 | 1.53 | 1.98E-19 | 9.1E-16 | 2.89 |

| | | | | |
|---|---|---|---|---|
| KIF15 | 1.52 | 3.94E-05 | 3.5E-03 | 2.87 |
| PROCR | 1.52 | 2.24E-05 | 2.3E-03 | 2.87 |
| NUF2 | 1.51 | 8.54E-05 | 6.2E-03 | 2.84 |
| ETV7 | 1.48 | 8.41E-04 | 3.1E-02 | 2.79 |
| IL2RA | 1.48 | 6.91E-10 | 3.5E-07 | 2.79 |
| MC1R | 1.48 | 1.97E-03 | 5.6E-02 | 2.79 |
| FCGR1C | 1.48 | 3.03E-05 | 3.0E-03 | 2.78 |
| MX1 | 1.47 | 5.28E-10 | 2.8E-07 | 2.77 |
| IFI6 | 1.46 | 2.13E-09 | 9.1E-07 | 2.74 |
| GCH1 | 1.46 | 7.29E-07 | 1.6E-04 | 2.74 |
| APOBEC3B | 1.45 | 1.08E-04 | 7.3E-03 | 2.74 |
| OSM | 1.44 | 6.52E-07 | 1.5E-04 | 2.71 |
| CCL18 | 1.39 | 1.06E-08 | 3.9E-06 | 2.63 |
| E2F8 | 1.39 | 5.50E-04 | 2.3E-02 | 2.63 |
| LOC100130872 | 1.38 | 1.17E-03 | 3.9E-02 | 2.61 |
| MAOA | 1.37 | 7.84E-09 | 3.0E-06 | 2.58 |
| C1R | 1.36 | 7.39E-03 | 1.4E-01 | 2.57 |
| SKA1 | 1.35 | 7.06E-05 | 5.5E-03 | 2.56 |
| ASPM | 1.34 | 1.02E-06 | 2.1E-04 | 2.52 |
| FCGR3A | 1.31 | 3.43E-11 | 2.3E-08 | 2.48 |
| MYO1G | 1.31 | 6.74E-15 | 1.6E-11 | 2.48 |
| CXCL10 | 1.30 | 7.38E-07 | 1.6E-04 | 2.46 |
| ISG15 | 1.30 | 3.00E-07 | 7.7E-05 | 2.46 |
| SYT17 | 1.29 | 3.31E-03 | 7.9E-02 | 2.44 |
| CDCA7 | 1.28 | 2.19E-03 | 6.0E-02 | 2.44 |
| TPX2 | 1.28 | 1.81E-05 | 2.0E-03 | 2.43 |
| NEURL1B | 1.27 | 4.57E-03 | 9.7E-02 | 2.42 |
| MAP3K7CL | 1.27 | 6.68E-04 | 2.6E-02 | 2.42 |
| NEK2 | 1.27 | 1.25E-03 | 4.0E-02 | 2.41 |
| CEP55 | 1.27 | 6.34E-06 | 8.9E-04 | 2.41 |
| GTSE1 | 1.27 | 8.55E-06 | 1.1E-03 | 2.40 |
| NDC80 | 1.26 | 1.75E-04 | 1.1E-02 | 2.40 |
| FCGR1A | 1.26 | 2.48E-11 | 1.9E-08 | 2.40 |
| NCAPG | 1.25 | 3.43E-05 | 3.2E-03 | 2.37 |
| CMPK2 | 1.25 | 4.71E-09 | 1.9E-06 | 2.37 |
| IFITM3 | 1.24 | 4.74E-06 | 6.9E-04 | 2.36 |

| | | | | |
|---|---|---|---|---|
| CENPF | 1.24 | 5.57E-07 | 1.3E-04 | 2.36 |
| FCGR3B | 1.24 | 2.01E-03 | 5.7E-02 | 2.36 |
| FCGR1B | 1.23 | 2.25E-04 | 1.3E-02 | 2.35 |
| DLGAP5 | 1.23 | 3.52E-05 | 3.2E-03 | 2.35 |
| MELK | 1.23 | 8.92E-05 | 6.4E-03 | 2.35 |
| TROAP | 1.23 | 5.13E-03 | 1.1E-01 | 2.34 |
| IFIT2 | 1.22 | 1.61E-09 | 7.4E-07 | 2.34 |
| BUB1B | 1.22 | 2.37E-04 | 1.3E-02 | 2.32 |
| OAS2 | 1.21 | 3.10E-12 | 3.0E-09 | 2.32 |
| HMMR | 1.21 | 3.42E-05 | 3.2E-03 | 2.31 |
| RND3 | 1.21 | 1.43E-03 | 4.4E-02 | 2.31 |
| CIT | 1.21 | 5.16E-05 | 4.3E-03 | 2.31 |
| SERPINA1 | 1.20 | 4.62E-07 | 1.1E-04 | 2.30 |
| ANLN | 1.19 | 3.05E-05 | 3.0E-03 | 2.29 |
| LAMB3 | 1.19 | 2.43E-05 | 2.5E-03 | 2.28 |
| TNF | 1.18 | 5.93E-05 | 4.8E-03 | 2.26 |
| DIAPH3 | 1.18 | 4.97E-03 | 1.0E-01 | 2.26 |
| USP18 | 1.17 | 2.06E-06 | 3.6E-04 | 2.25 |
| KIF20A | 1.17 | 2.16E-04 | 1.2E-02 | 2.25 |
| HJURP | 1.16 | 5.16E-04 | 2.2E-02 | 2.24 |
| UBE2C | 1.15 | 4.19E-05 | 3.6E-03 | 2.22 |
| ABCG1 | 1.15 | 4.90E-07 | 1.2E-04 | 2.22 |
| CASC5 | 1.14 | 9.86E-04 | 3.5E-02 | 2.21 |
| KIF14 | 1.14 | 4.43E-03 | 9.5E-02 | 2.21 |
| TYMS | 1.14 | 1.94E-04 | 1.2E-02 | 2.20 |
| MT1X | 1.14 | 2.13E-04 | 1.2E-02 | 2.20 |
| CXCR2P1 | 1.14 | 4.62E-04 | 2.1E-02 | 2.20 |
| CCNB2 | 1.13 | 4.16E-04 | 1.9E-02 | 2.19 |
| FAM111B | 1.13 | 3.81E-04 | 1.8E-02 | 2.18 |
| OAS3 | 1.12 | 4.47E-11 | 2.9E-08 | 2.18 |
| LY6E | 1.12 | 1.42E-06 | 2.8E-04 | 2.17 |
| B3GNT5 | 1.11 | 2.86E-05 | 2.8E-03 | 2.17 |
| IL7R | 1.11 | 1.56E-05 | 1.8E-03 | 2.16 |
| GPSM2 | 1.11 | 5.23E-03 | 1.1E-01 | 2.15 |
| TTK | 1.10 | 1.44E-03 | 4.4E-02 | 2.15 |
| CCNA2 | 1.10 | 7.07E-07 | 1.6E-04 | 2.15 |

| | | | | |
|---|---|---|---|---|
| DEPDC1 | 1.10 | 7.57E-03 | 1.4E-01 | 2.14 |
| OASL | 1.10 | 1.91E-04 | 1.1E-02 | 2.14 |
| TMEM52B | 1.10 | 5.51E-03 | 1.1E-01 | 2.14 |
| SHCBP1 | 1.09 | 1.22E-03 | 3.9E-02 | 2.13 |
| CD226 | 1.09 | 2.34E-04 | 1.3E-02 | 2.12 |
| CDK1 | 1.09 | 4.90E-04 | 2.1E-02 | 2.12 |
| CDC45 | 1.08 | 7.78E-03 | 1.4E-01 | 2.11 |
| SAMD9L | 1.08 | 2.56E-09 | 1.1E-06 | 2.11 |
| RMI2 | 1.07 | 7.93E-03 | 1.5E-01 | 2.10 |
| KIF4A | 1.07 | 1.47E-03 | 4.5E-02 | 2.10 |
| GGH | 1.07 | 4.83E-04 | 2.1E-02 | 2.10 |
| MKI67 | 1.06 | 7.00E-06 | 9.4E-04 | 2.09 |
| MT1E | 1.06 | 3.56E-04 | 1.8E-02 | 2.08 |
| VDR | 1.06 | 1.12E-03 | 3.8E-02 | 2.08 |
| AURKB | 1.06 | 4.91E-04 | 2.1E-02 | 2.08 |
| PLK1 | 1.06 | 1.87E-03 | 5.4E-02 | 2.08 |
| CLSPN | 1.05 | 2.29E-04 | 1.3E-02 | 2.07 |
| KIAA0101 | 1.05 | 6.03E-04 | 2.4E-02 | 2.07 |
| IFIT1 | 1.05 | 1.78E-04 | 1.1E-02 | 2.07 |
| C15orf48 | 1.04 | 2.95E-04 | 1.5E-02 | 2.06 |
| KIF18B | 1.04 | 1.87E-03 | 5.4E-02 | 2.06 |
| DTL | 1.04 | 4.90E-03 | 1.0E-01 | 2.05 |
| TOP2A | 1.03 | 1.20E-06 | 2.4E-04 | 2.05 |
| POLQ | 1.03 | 7.66E-03 | 1.4E-01 | 2.04 |
| JAKMIP2 | 1.03 | 1.36E-07 | 3.8E-05 | 2.04 |
| DDX60 | 1.03 | 1.78E-06 | 3.2E-04 | 2.04 |
| BRIP1 | 1.02 | 2.21E-03 | 6.0E-02 | 2.03 |
| FOXM1 | 1.02 | 4.59E-04 | 2.1E-02 | 2.03 |
| CD28 | 1.02 | 2.81E-04 | 1.5E-02 | 2.03 |
| NUSAP1 | 1.02 | 4.49E-06 | 6.7E-04 | 2.03 |
| MAD2L1 | 1.02 | 5.28E-04 | 2.2E-02 | 2.02 |
| EPSTI1 | 1.01 | 8.14E-05 | 6.1E-03 | 2.02 |
| TRPV4 | 1.01 | 4.04E-04 | 1.9E-02 | 2.02 |
| CDC20 | 1.01 | 6.44E-04 | 2.5E-02 | 2.02 |
| WEE1 | 1.01 | 8.76E-04 | 3.2E-02 | 2.01 |
| SMPDL3A | 1.01 | 2.63E-03 | 6.7E-02 | 2.01 |

| | | | | |
|---|---|---|---|---|
| IL15RA | 1.01 | 3.73E-04 | 1.8E-02 | 2.01 |
| LAIR2 | 1.00 | 3.00E-03 | 7.3E-02 | 2.00 |
| PARP9 | 1.00 | 2.51E-08 | 8.1E-06 | 2.00 |
| IFI44 | 0.99 | 4.97E-06 | 7.2E-04 | 1.99 |
| KIF2C | 0.98 | 3.98E-03 | 8.8E-02 | 1.98 |
| ANOS1 | 0.98 | 1.39E-05 | 1.7E-03 | 1.98 |
| PTTG1 | 0.98 | 2.08E-03 | 5.9E-02 | 1.97 |
| SPAG5 | 0.97 | 1.22E-03 | 3.9E-02 | 1.96 |
| LGALS3BP | 0.97 | 1.97E-04 | 1.2E-02 | 1.96 |
| MFGE8 | 0.97 | 2.97E-04 | 1.5E-02 | 1.96 |
| RRM2 | 0.97 | 5.68E-04 | 2.4E-02 | 1.96 |
| EIF2AK2 | 0.96 | 4.31E-06 | 6.5E-04 | 1.95 |
| ATP10A | 0.96 | 2.92E-03 | 7.2E-02 | 1.94 |
| RNASE2 | 0.95 | 3.40E-03 | 8.0E-02 | 1.94 |
| FPR2 | 0.95 | 1.78E-03 | 5.3E-02 | 1.93 |
| KIF11 | 0.95 | 1.65E-05 | 1.8E-03 | 1.93 |
| *Downregulated* | | | | |
| GFRA2 | -3.27 | 2.74E-05 | 2.8E-03 | 0.10 |
| ROR2 | -2.57 | 3.97E-05 | 3.5E-03 | 0.17 |
| F2RL3 | -2.34 | 3.57E-04 | 1.8E-02 | 0.20 |
| SERPINB2 | -2.31 | 2.44E-03 | 6.4E-02 | 0.20 |
| COL4A1 | -2.27 | 1.28E-05 | 1.6E-03 | 0.21 |
| CFP | -2.18 | 5.29E-18 | 1.5E-14 | 0.22 |
| WFIKKN2 | -2.15 | 1.29E-03 | 4.1E-02 | 0.23 |
| RNF128 | -2.04 | 3.24E-04 | 1.7E-02 | 0.24 |
| GJA5 | -2.00 | 2.52E-06 | 4.2E-04 | 0.25 |
| THBS1 | -1.85 | 2.18E-04 | 1.2E-02 | 0.28 |
| TMEM114 | -1.81 | 2.40E-06 | 4.1E-04 | 0.29 |
| CD163L1 | -1.78 | 1.91E-12 | 2.2E-09 | 0.29 |
| GPER1 | -1.76 | 2.73E-04 | 1.5E-02 | 0.29 |
| ITGAD | -1.76 | 3.96E-05 | 3.5E-03 | 0.29 |
| SPTBN2 | -1.74 | 8.08E-05 | 6.1E-03 | 0.30 |
| LRP5 | -1.73 | 1.96E-03 | 5.6E-02 | 0.30 |
| VCAM1 | -1.73 | 1.10E-09 | 5.3E-07 | 0.30 |
| APLN | -1.73 | 1.36E-03 | 4.2E-02 | 0.30 |
| SIX5 | -1.70 | 2.81E-03 | 7.1E-02 | 0.31 |

| | | | | |
|---|---|---|---|---|
| TPD52L1 | -1.67 | 5.75E-04 | 2.4E-02 | 0.31 |
| TMEM151A | -1.65 | 1.28E-03 | 4.1E-02 | 0.32 |
| FABP4 | -1.64 | 2.52E-11 | 1.9E-08 | 0.32 |
| LTC4S | -1.64 | 2.76E-03 | 7.0E-02 | 0.32 |
| CYGB | -1.63 | 8.07E-08 | 2.3E-05 | 0.32 |
| C10orf105 | -1.63 | 8.92E-04 | 3.2E-02 | 0.32 |
| PELI2 | -1.63 | 4.14E-05 | 3.6E-03 | 0.32 |
| BCAM | -1.62 | 1.89E-04 | 1.1E-02 | 0.32 |
| DACT1 | -1.62 | 4.62E-03 | 9.8E-02 | 0.32 |
| CD300LB | -1.62 | 3.75E-08 | 1.1E-05 | 0.32 |
| GALNT14 | -1.62 | 2.65E-04 | 1.4E-02 | 0.33 |
| PTPRF | -1.61 | 2.19E-03 | 6.0E-02 | 0.33 |
| SPOCD1 | -1.58 | 1.08E-08 | 3.9E-06 | 0.34 |
| CKB | -1.57 | 2.31E-12 | 2.4E-09 | 0.34 |
| UTS2 | -1.57 | 3.81E-03 | 8.6E-02 | 0.34 |
| SDC1 | -1.57 | 2.60E-06 | 4.3E-04 | 0.34 |
| GUCY1A2 | -1.56 | 7.11E-04 | 2.7E-02 | 0.34 |
| SCG5 | -1.55 | 3.70E-03 | 8.4E-02 | 0.34 |
| SPIC | -1.54 | 3.26E-03 | 7.8E-02 | 0.34 |
| TPM2 | -1.54 | 4.76E-03 | 1.0E-01 | 0.34 |
| DRAXIN | -1.51 | 3.05E-04 | 1.6E-02 | 0.35 |
| HTR7 | -1.50 | 5.15E-06 | 7.4E-04 | 0.35 |
| CLMP | -1.49 | 1.59E-05 | 1.8E-03 | 0.36 |
| HTRA3 | -1.49 | 3.45E-03 | 8.1E-02 | 0.36 |
| PID1 | -1.48 | 1.43E-03 | 4.4E-02 | 0.36 |
| PIGR | -1.48 | 7.28E-03 | 1.4E-01 | 0.36 |
| PDK4 | -1.44 | 4.62E-05 | 4.0E-03 | 0.37 |
| MYO1A | -1.44 | 3.62E-03 | 8.3E-02 | 0.37 |
| FAT1 | -1.43 | 5.30E-03 | 1.1E-01 | 0.37 |
| TACC2 | -1.42 | 2.19E-03 | 6.0E-02 | 0.37 |
| FCN1 | -1.42 | 4.16E-13 | 8.0E-10 | 0.37 |
| TMEM119 | -1.39 | 7.39E-05 | 5.7E-03 | 0.38 |
| TIMP3 | -1.38 | 7.04E-13 | 1.0E-09 | 0.38 |
| CCDC152 | -1.37 | 6.39E-04 | 2.5E-02 | 0.39 |
| TNFRSF4 | -1.37 | 1.17E-03 | 3.8E-02 | 0.39 |
| HTR2B | -1.36 | 1.09E-03 | 3.7E-02 | 0.39 |

| | | | | |
|---|---|---|---|---|
| COBLL1 | -1.36 | 3.48E-05 | 3.2E-03 | 0.39 |
| DNASE2B | -1.36 | 1.51E-06 | 2.9E-04 | 0.39 |
| KAZN | -1.34 | 2.16E-03 | 5.9E-02 | 0.40 |
| MTSS1L | -1.34 | 1.01E-03 | 3.5E-02 | 0.40 |
| RGS20 | -1.33 | 1.06E-04 | 7.3E-03 | 0.40 |
| ALDH1A2 | -1.33 | 4.55E-03 | 9.7E-02 | 0.40 |
| TM4SF19 | -1.33 | 1.69E-03 | 5.1E-02 | 0.40 |
| ITGA3 | -1.33 | 2.73E-06 | 4.4E-04 | 0.40 |
| GAL | -1.32 | 2.13E-09 | 9.1E-07 | 0.40 |
| OSBP2 | -1.30 | 8.40E-04 | 3.1E-02 | 0.41 |
| ADRA2B | -1.30 | 1.65E-06 | 3.1E-04 | 0.41 |
| ASPHD1 | -1.30 | 6.17E-04 | 2.5E-02 | 0.41 |
| FCMR | -1.29 | 1.13E-08 | 4.0E-06 | 0.41 |
| DMWD | -1.25 | 3.65E-04 | 1.8E-02 | 0.42 |
| LINGO1 | -1.25 | 6.21E-03 | 1.2E-01 | 0.42 |
| KCNAB1 | -1.24 | 3.39E-05 | 3.2E-03 | 0.42 |
| USP2 | -1.22 | 2.50E-08 | 8.1E-06 | 0.43 |
| AFAP1L1 | -1.21 | 3.87E-07 | 9.5E-05 | 0.43 |
| GPD1 | -1.20 | 3.46E-06 | 5.3E-04 | 0.43 |
| DUSP13 | -1.19 | 1.08E-03 | 3.7E-02 | 0.44 |
| MYL9 | -1.19 | 3.53E-08 | 1.1E-05 | 0.44 |
| ANTXR1 | -1.18 | 2.84E-03 | 7.1E-02 | 0.44 |
| ARFGEF3 | -1.17 | 5.81E-03 | 1.2E-01 | 0.44 |
| TESC | -1.17 | 3.33E-04 | 1.7E-02 | 0.44 |
| DGAT2 | -1.17 | 9.31E-04 | 3.4E-02 | 0.44 |
| CPAMD8 | -1.17 | 7.54E-04 | 2.8E-02 | 0.44 |
| RASAL1 | -1.17 | 5.40E-05 | 4.5E-03 | 0.45 |
| PDLIM4 | -1.17 | 1.04E-05 | 1.3E-03 | 0.45 |
| GDF15 | -1.16 | 2.07E-06 | 3.6E-04 | 0.45 |
| DEPTOR | -1.15 | 1.35E-03 | 4.2E-02 | 0.45 |
| CCL22 | -1.15 | 9.88E-11 | 6.0E-08 | 0.45 |
| PAQR7 | -1.15 | 3.36E-04 | 1.7E-02 | 0.45 |
| HES2 | -1.14 | 8.48E-05 | 6.2E-03 | 0.45 |
| SCIN | -1.14 | 1.09E-04 | 7.3E-03 | 0.46 |
| LINC00520 | -1.13 | 3.36E-03 | 8.0E-02 | 0.46 |
| OBSL1 | -1.13 | 8.18E-04 | 3.1E-02 | 0.46 |

| | | | | |
|---|---|---|---|---|
| SLAMF9 | -1.13 | 7.98E-04 | 3.0E-02 | 0.46 |
| GPC4 | -1.12 | 2.57E-10 | 1.4E-07 | 0.46 |
| SLC2A4 | -1.12 | 4.01E-03 | 8.9E-02 | 0.46 |
| CD320 | -1.11 | 1.29E-04 | 8.3E-03 | 0.46 |
| ASB2 | -1.11 | 3.93E-03 | 8.8E-02 | 0.46 |
| MMP15 | -1.10 | 1.09E-03 | 3.7E-02 | 0.47 |
| ALPK3 | -1.09 | 5.68E-05 | 4.7E-03 | 0.47 |
| RAPGEF3 | -1.09 | 3.81E-04 | 1.8E-02 | 0.47 |
| TSPAN13 | -1.09 | 6.14E-03 | 1.2E-01 | 0.47 |
| MATK | -1.08 | 2.35E-08 | 8.0E-06 | 0.47 |
| MMP25 | -1.08 | 1.08E-04 | 7.3E-03 | 0.47 |
| CXCL2 | -1.08 | 2.19E-05 | 2.3E-03 | 0.47 |
| DUSP1 | -1.08 | 9.55E-05 | 6.8E-03 | 0.47 |
| KCNJ1 | -1.08 | 2.74E-03 | 7.0E-02 | 0.47 |
| DPYSL3 | -1.08 | 7.20E-06 | 9.6E-04 | 0.47 |
| SPON2 | -1.07 | 2.92E-05 | 2.9E-03 | 0.48 |
| CHIT1 | -1.07 | 3.12E-06 | 5.0E-04 | 0.48 |
| PDE6G | -1.06 | 3.27E-04 | 1.7E-02 | 0.48 |
| RASGRF1 | -1.06 | 7.85E-05 | 5.9E-03 | 0.48 |
| NR1D1 | -1.05 | 3.75E-03 | 8.5E-02 | 0.48 |
| AOC1 | -1.05 | 4.87E-04 | 2.1E-02 | 0.48 |
| DCANP1 | -1.05 | 4.19E-05 | 3.6E-03 | 0.48 |
| COL6A1 | -1.05 | 2.92E-07 | 7.7E-05 | 0.48 |
| PODXL | -1.05 | 2.63E-03 | 6.7E-02 | 0.48 |
| CCDC28B | -1.03 | 1.39E-03 | 4.3E-02 | 0.49 |
| NES | -1.03 | 4.18E-05 | 3.6E-03 | 0.49 |
| FCGR2B | -1.02 | 9.55E-05 | 6.8E-03 | 0.49 |
| CSPG4 | -1.02 | 6.17E-04 | 2.5E-02 | 0.49 |
| MFI2 | -1.02 | 7.47E-04 | 2.8E-02 | 0.49 |
| TNFRSF10D | -1.02 | 1.74E-04 | 1.1E-02 | 0.49 |
| MAP3K14 | -1.01 | 2.20E-07 | 5.9E-05 | 0.50 |
| BCAR3 | -1.01 | 4.78E-05 | 4.1E-03 | 0.50 |
| RAB6B | -1.01 | 5.57E-04 | 2.3E-02 | 0.50 |
| OCSTAMP | -1.01 | 1.69E-04 | 1.0E-02 | 0.50 |
| ZNF704 | -1.00 | 7.16E-04 | 2.8E-02 | 0.50 |
| DHRS11 | -1.00 | 1.54E-04 | 9.7E-03 | 0.50 |

| | | | | |
|---|---|---|---|---|
| IQCK | -1.00 | 2.54E-03 | 6.6E-02 | 0.50 |
| SLC30A3 | -1.00 | 2.53E-03 | 6.6E-02 | 0.50 |
| TTYH2 | -1.00 | 9.74E-05 | 6.8E-03 | 0.50 |
| HSPG2 | -1.00 | 2.06E-03 | 5.8E-02 | 0.50 |
| GOLGA7B | -1.00 | 4.20E-03 | 9.2E-02 | 0.50 |
| ZNF395 | -1.00 | 4.41E-04 | 2.0E-02 | 0.50 |
| CRABP2 | -0.99 | 1.23E-07 | 3.5E-05 | 0.50 |
| SRPX | -0.98 | 5.26E-03 | 1.1E-01 | 0.51 |
| TIFAB | -0.98 | 1.18E-04 | 7.8E-03 | 0.51 |
| SMPD3 | -0.97 | 7.36E-03 | 1.4E-01 | 0.51 |
| CACNA2D3 | -0.97 | 4.40E-03 | 9.4E-02 | 0.51 |
| STEAP4 | -0.97 | 4.07E-03 | 9.0E-02 | 0.51 |
| SYNGR1 | -0.97 | 1.88E-04 | 1.1E-02 | 0.51 |
| FAM13A | -0.97 | 1.03E-03 | 3.6E-02 | 0.51 |
| DUSP2 | -0.96 | 1.84E-05 | 2.0E-03 | 0.51 |
| SEMA4C | -0.96 | 2.01E-03 | 5.7E-02 | 0.52 |
| FHL1 | -0.96 | 1.14E-03 | 3.8E-02 | 0.52 |
