# Supplementary material for: Transcriptome Profiling of Human Monocyte-Derived Macrophages Upon CCL2 Neutralization Reveals an Association Between Activation of Innate Immune Pathways and Restriction of HIV-1 Gene Expression
Source: Front Immunol. 2020 Sep 18;11:2129. doi: 10.3389/fimmu.2020.02129 (PMC7531389; doi:10.3389/fimmu.2020.02129)
Supplement: Supplementary file 2 [file Data_Sheet_2.zip › Supplementary Figure S1.PDF]

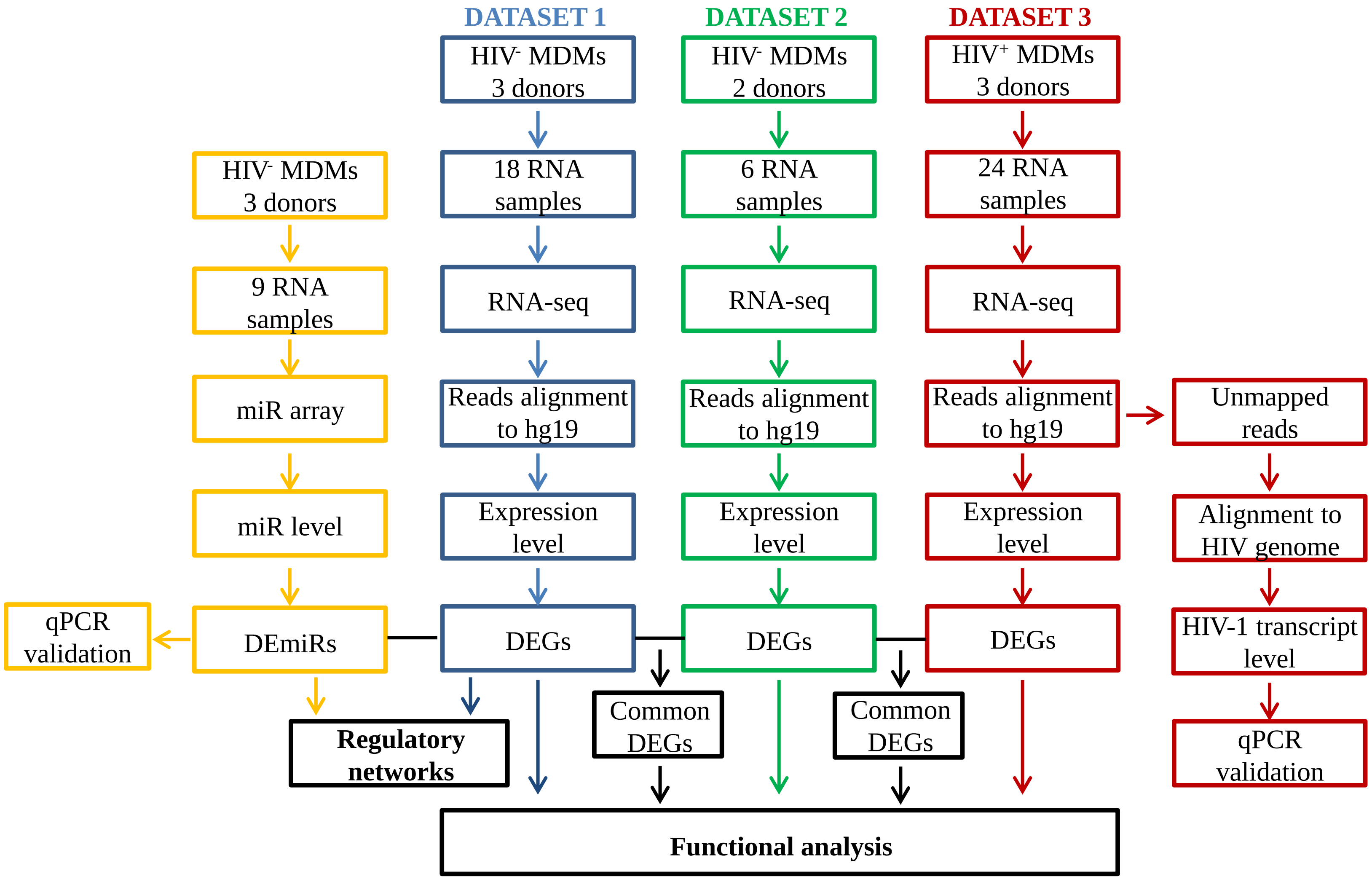

Supplementary Figure 1. Schematic illustration of the overall study design, data generation and processing. Three RNA-seq datasets were generated from monocyte-derived macrophages (MDMs) obtained from 8 donors. Cells were treated or not with anti-CCL2 or control Ab for 4 or 20 h (dataset 1), 4 h (dataset 2), 4 h before HIV-1 infection for 1 or 4 days (dataset 3). Total RNA was extracted and deep sequenced using next-generation sequencing platforms. Reads were aligned to hg19 and the differentially expressed genes (DEGs) were identified using DESeq2. Functional analysis was performed using DAVID and TRRUST. Reads which failed to align to hg19 were mapped to the HIV-1BaL genome to quantify viral transcripts. MDMs obtained from three additional donors were treated or not with anti-CCL2 or control Ab for 4 h, and the total RNA extracted was used to perform miRNA expression profiling and identify the differentially expressed miRNAs (DEmiRs). Regulatory networks among DEGs and DEmiRs were identified by bioinformatics/literature search. The expression profile of selected HIV-1 transcripts and miRs was confirmed by quantitative RT-PCR (qPCR) on MDM samples used in the RNA-seq/array experiments or obtained from 5 additional donors.
