# Supplementary material for: Transcriptome Profiling of Human Monocyte-Derived Macrophages Upon CCL2 Neutralization Reveals an Association Between Activation of Innate Immune Pathways and Restriction of HIV-1 Gene Expression
Source: Front Immunol. 2020 Sep 18;11:2129. doi: 10.3389/fimmu.2020.02129 (PMC7531389; doi:10.3389/fimmu.2020.02129)
Supplement: Supplementary file 2 [file Data_Sheet_2.zip › Supplementary Figure S2.PDF]

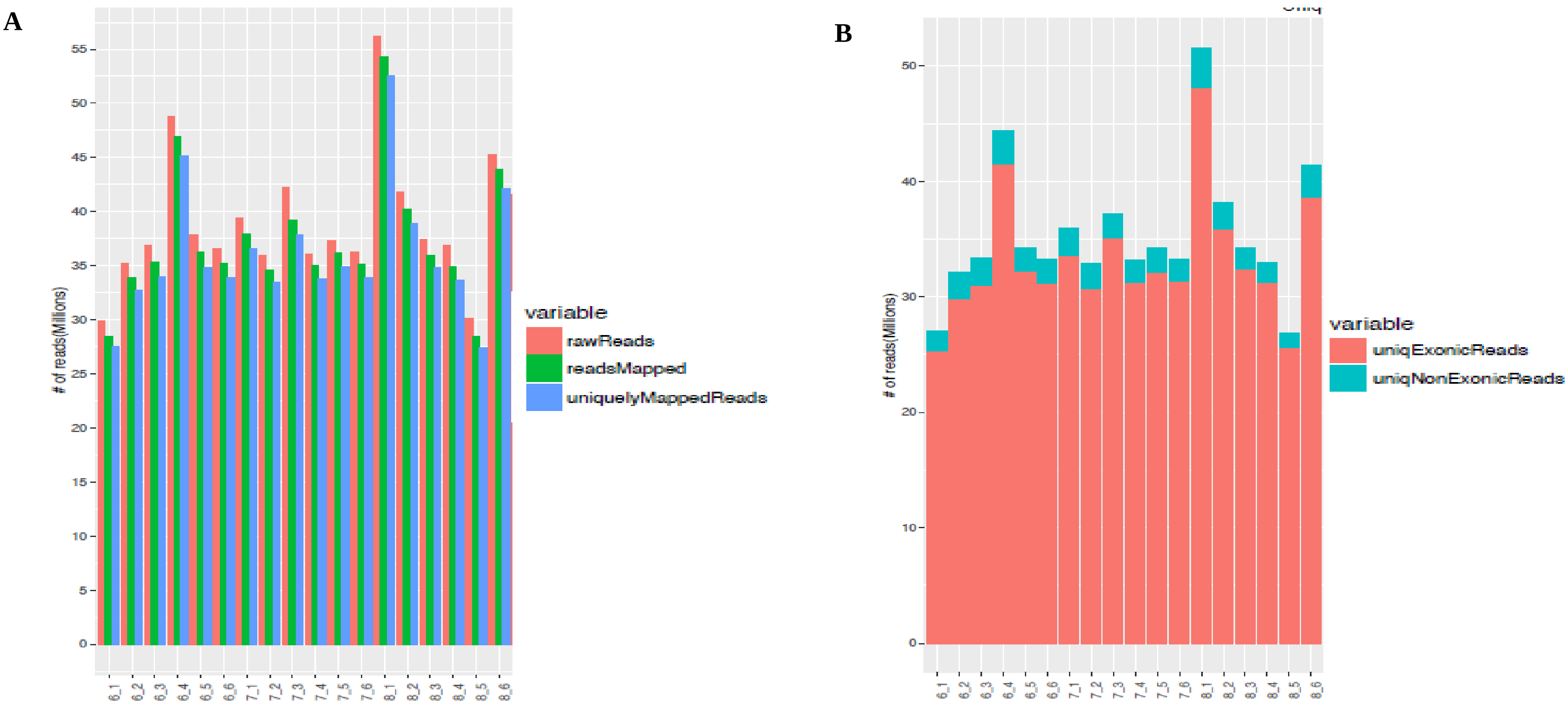

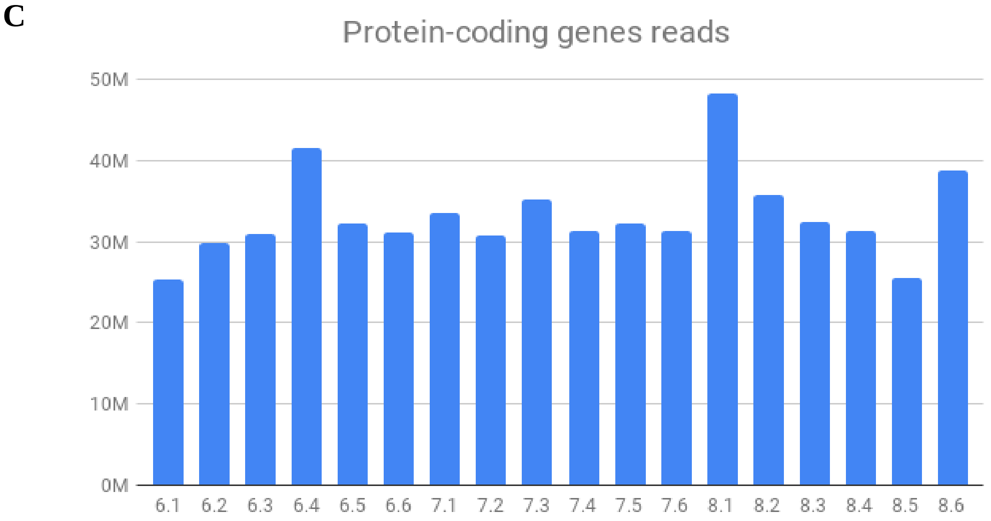

Supplementary Figure 2. Descriptive statistics of RNA-seq dataset 1. MDMs obtained from 3 different donors were treated or not with anti-CCL2 or control Ab (2.5 µg/ml) for 4 or 20 h. Total RNA was extracted, subjected to poly(A) selection followed by reverse transcription, generation of cDNA libraries, and sequencing. (A) Raw, mapped and uniquely mapped reads per sample. (B) Uniquely exonic and non-exonic reads per sample. (C) Protein-coding genes reads per sample. The samples numeric codes in the x axis identify the different donors (first number) and conditions (second number; 1 = nil 4 h, 2 = anti-CCL2 Ab 4 h, 3 = control Ab 4 h, 4 = nil 20 h, 5 = anti-CCL2 Ab 20 h, 6 = control Ab 20 h).
