# Supplementary material for: Transcriptome Profiling of Human Monocyte-Derived Macrophages Upon CCL2 Neutralization Reveals an Association Between Activation of Innate Immune Pathways and Restriction of HIV-1 Gene Expression
Source: Front Immunol. 2020 Sep 18;11:2129. doi: 10.3389/fimmu.2020.02129 (PMC7531389; doi:10.3389/fimmu.2020.02129)
Supplement: Supplementary file 2 [file Data_Sheet_2.zip › Supplementary Figure S3.PDF]

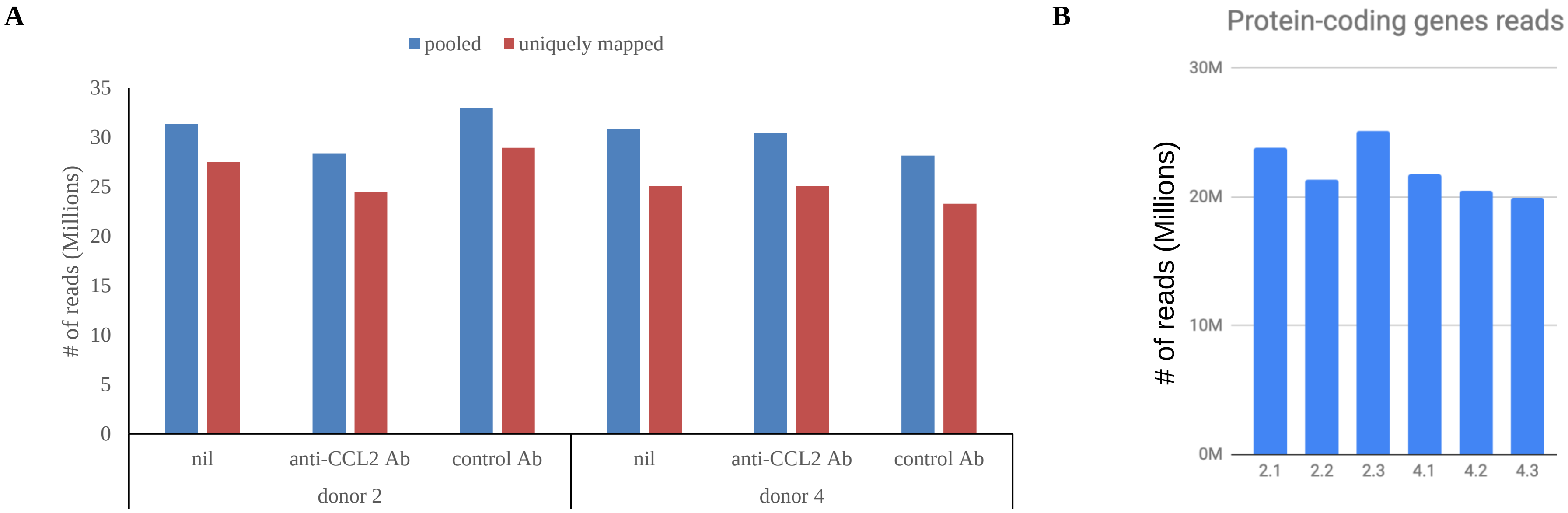

Supplementary Figure 3. Descriptive statistics of RNA-seq dataset 2. MDMs obtained from 2 different donors were treated or not with anti-CCL2 or control Ab (2.5 µg/ml) for 4 h. Total RNA was extracted, subjected to poly(A) selection followed by reverse transcription, generation of cDNA libraries, and sequencing. (A) Raw and uniquely mapped reads per sample. (B) Protein-coding genes reads per sample. The numeric codes in the x axis identify the different donors (first number) and conditions (second number; 1 = nil 4 h, 2 = anti-CCL2 Ab 4 h, 3 = control Ab 4 h).
