# Supplementary material for: Transcriptome Profiling of Human Monocyte-Derived Macrophages Upon CCL2 Neutralization Reveals an Association Between Activation of Innate Immune Pathways and Restriction of HIV-1 Gene Expression
Source: Front Immunol. 2020 Sep 18;11:2129. doi: 10.3389/fimmu.2020.02129 (PMC7531389; doi:10.3389/fimmu.2020.02129)
Supplement: Supplementary file 2 [file Data_Sheet_2.zip › Supplementary Figure S4.PDF]

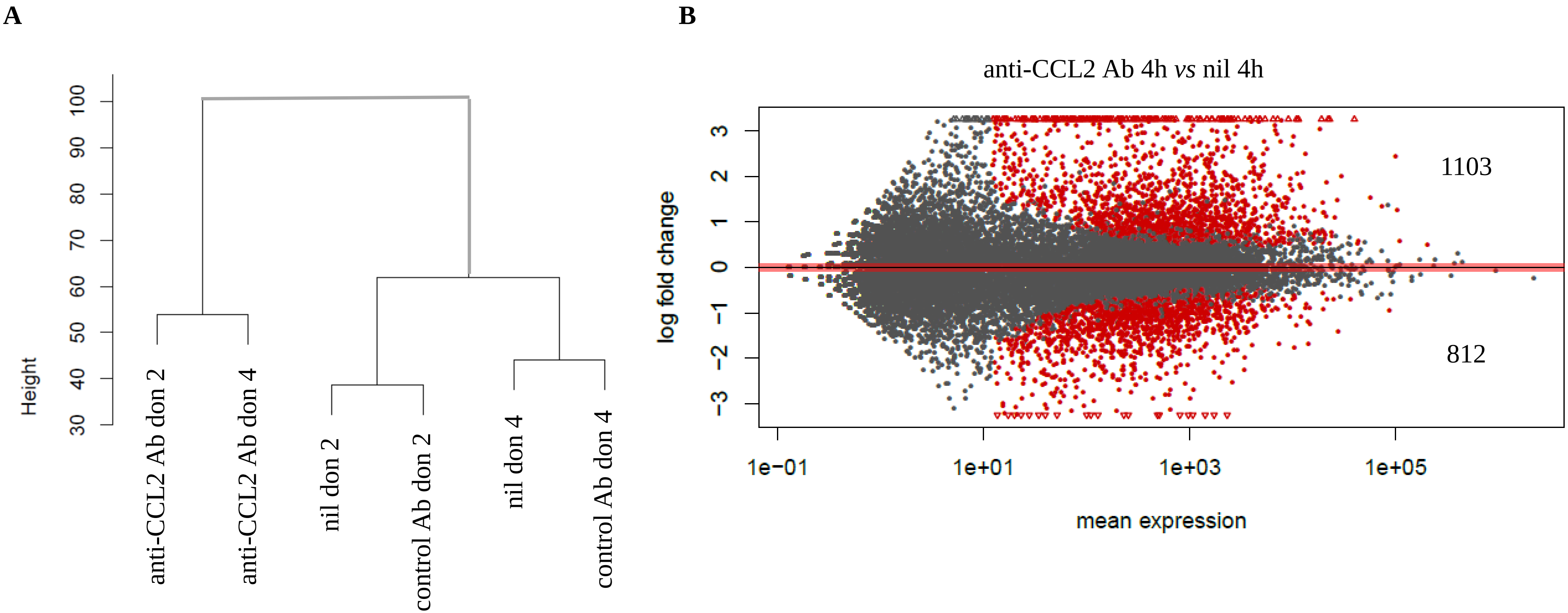

Supplementary Figure 4. Hierarchical clustering and differential expression analysis of dataset 2. MDMs obtained from 2 different donors were treated or not with anti-CCL2 or control Ab (2.5 µg/ml) for 4 h. Total RNA was extracted, subjected to poly(A) selection followed by reverse transcription, generation of cDNA libraries, and sequencing. (A) Hierarchical clustering of RNA-seq data. (B) Volcano plot representation of differential expression analysis of genes in the anti-CCL2 Ab 4h versus nil 4h comparison. A total of 1,915 transcripts (of whom 1103 and 812 were respectively up- and down-regulated) had a threshold adjusted p-value < 0.1 and log2 fold change ≥ 1 or ≤ -1.
