# Supplementary material for: Transcriptome Profiling of Human Monocyte-Derived Macrophages Upon CCL2 Neutralization Reveals an Association Between Activation of Innate Immune Pathways and Restriction of HIV-1 Gene Expression
Source: Front Immunol. 2020 Sep 18;11:2129. doi: 10.3389/fimmu.2020.02129 (PMC7531389; doi:10.3389/fimmu.2020.02129)
Supplement: Supplementary file 2 [file Data_Sheet_2.zip › Supplementary Figure S5.PDF]

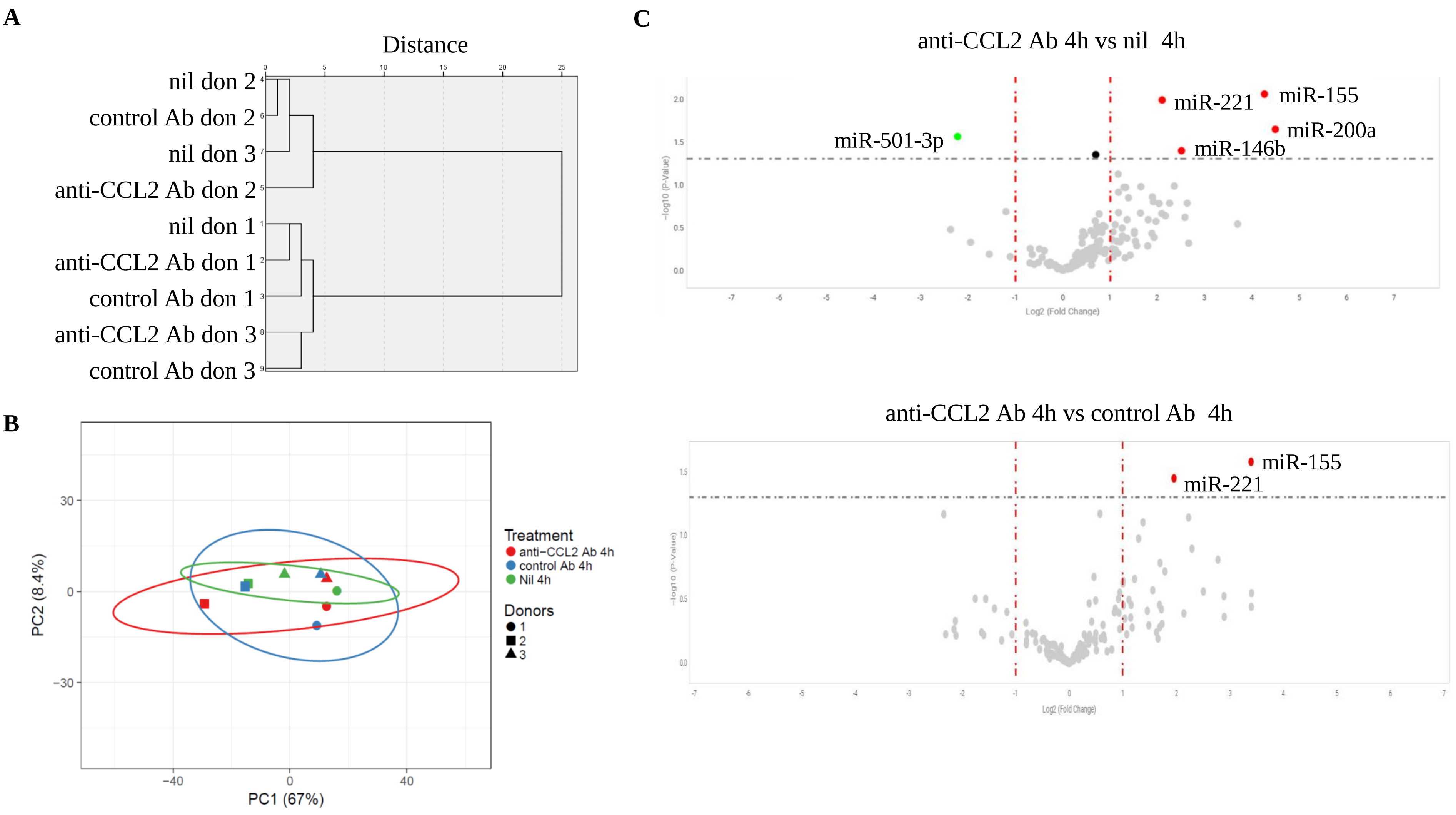

Supplementary Figure 5. Clustering and differential expression analysis of miRNA array data. MDMs obtained from 3 different donors were treated or not with anti-CCL2 or control Ab (2.5 μg/ml) for 4 h. Total RNA was extracted and subjected to reverse transcription and miRNA microarray analysis. (A) Cluster analysis dendrogram of array data. (B) Principal component analysis of array data. Unit variance scaling was applied to rows; SVD with imputation was used to calculate principal components; n = 24 data points. Prediction ellipses are such that with probability 0.95, a new observation from the same group will fall inside the ellipse. (C) Volcano plot representation of differential expression analysis (p value < 0.05 and FC > 2) of miRNAs in the anti-CCL2 Ab 4h versus nil 4h and anti-CCL2 Ab 4h versus control Ab 4h comparisons.
