# Supplementary material for: Transcriptome Profiling of Human Monocyte-Derived Macrophages Upon CCL2 Neutralization Reveals an Association Between Activation of Innate Immune Pathways and Restriction of HIV-1 Gene Expression
Source: Front Immunol. 2020 Sep 18;11:2129. doi: 10.3389/fimmu.2020.02129 (PMC7531389; doi:10.3389/fimmu.2020.02129)
Supplement: Supplementary file 2 [file Data_Sheet_2.zip › Supplementary Figure S6.PDF]

A

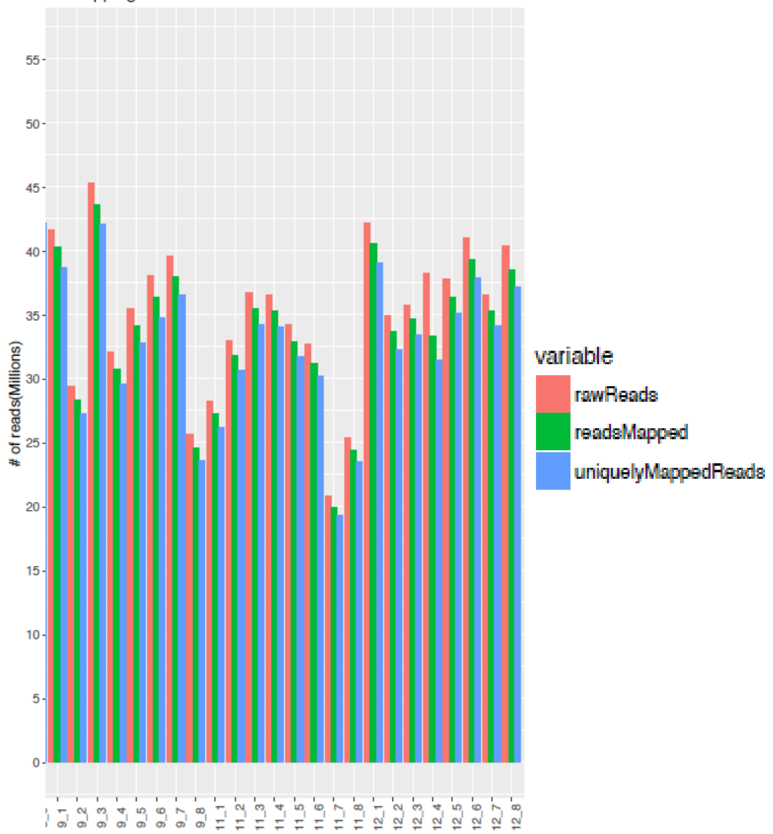

B

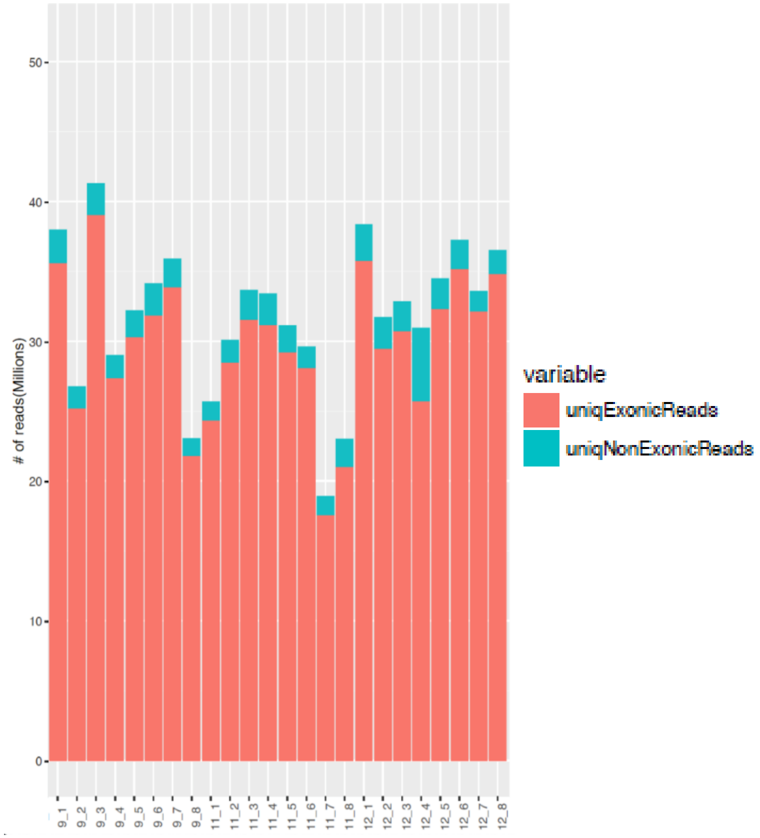

C

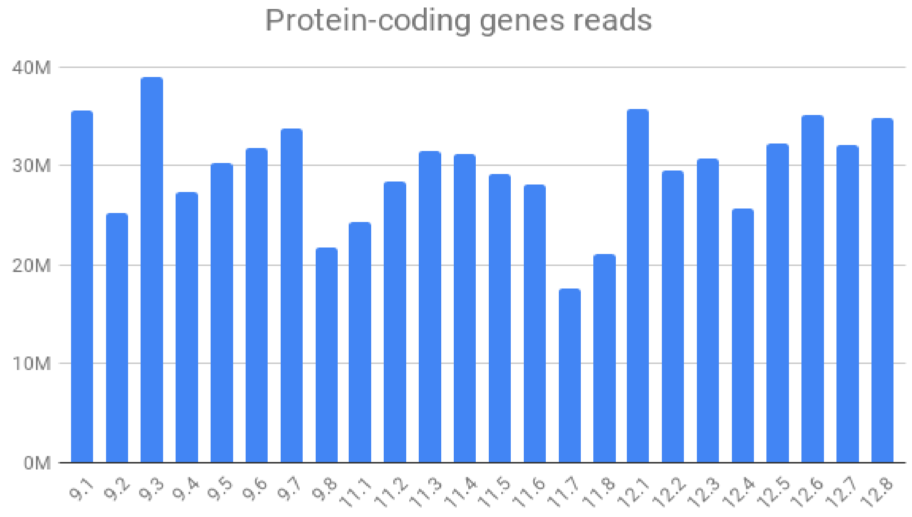

Supplementary Figure 6. Descriptive statistics of RNA-seq dataset 3. MDMs obtained from 3 different donors were treated or not with anti-CCL2 or control Ab (2.5 μg/ml) for 4 h and then infected with HIV-1. Total RNA was extracted at day 1 and 4 p.i., subjected to poly(A) selection followed by reverse transcription, generation of cDNA libraries, and sequencing. (A) Raw, mapped and uniquely mapped reads per sample. (B) Uniquely exonic and non-exonic reads per sample. (C) Protein-coding genes reads per sample. The samples numeric codes in the x axis identify the different donors (first number) and conditions (second number; 1 = nil day 1 p.i., 2 = HIV-1 day 1 p.i., 3 = HIV-1 + anti-CCL2 Ab day 1 p.i., 4 = HIV-1 + control Ab day 1 p.i., 5 = nil day 4 p.i., 6 = HIV-1 day 4 p.i., 7 = HIV-1 + anti-CCL2 Ab day 4 p.i., 8 = HIV-1 + control Ab day 4 p.i.).
