# Supplementary material for: Transcriptome Profiling of Human Monocyte-Derived Macrophages Upon CCL2 Neutralization Reveals an Association Between Activation of Innate Immune Pathways and Restriction of HIV-1 Gene Expression
Source: Front Immunol. 2020 Sep 18;11:2129. doi: 10.3389/fimmu.2020.02129 (PMC7531389; doi:10.3389/fimmu.2020.02129)
Supplement: Supplementary file 2 [file Data_Sheet_2.zip › Supplementary Figure S7.PDF]

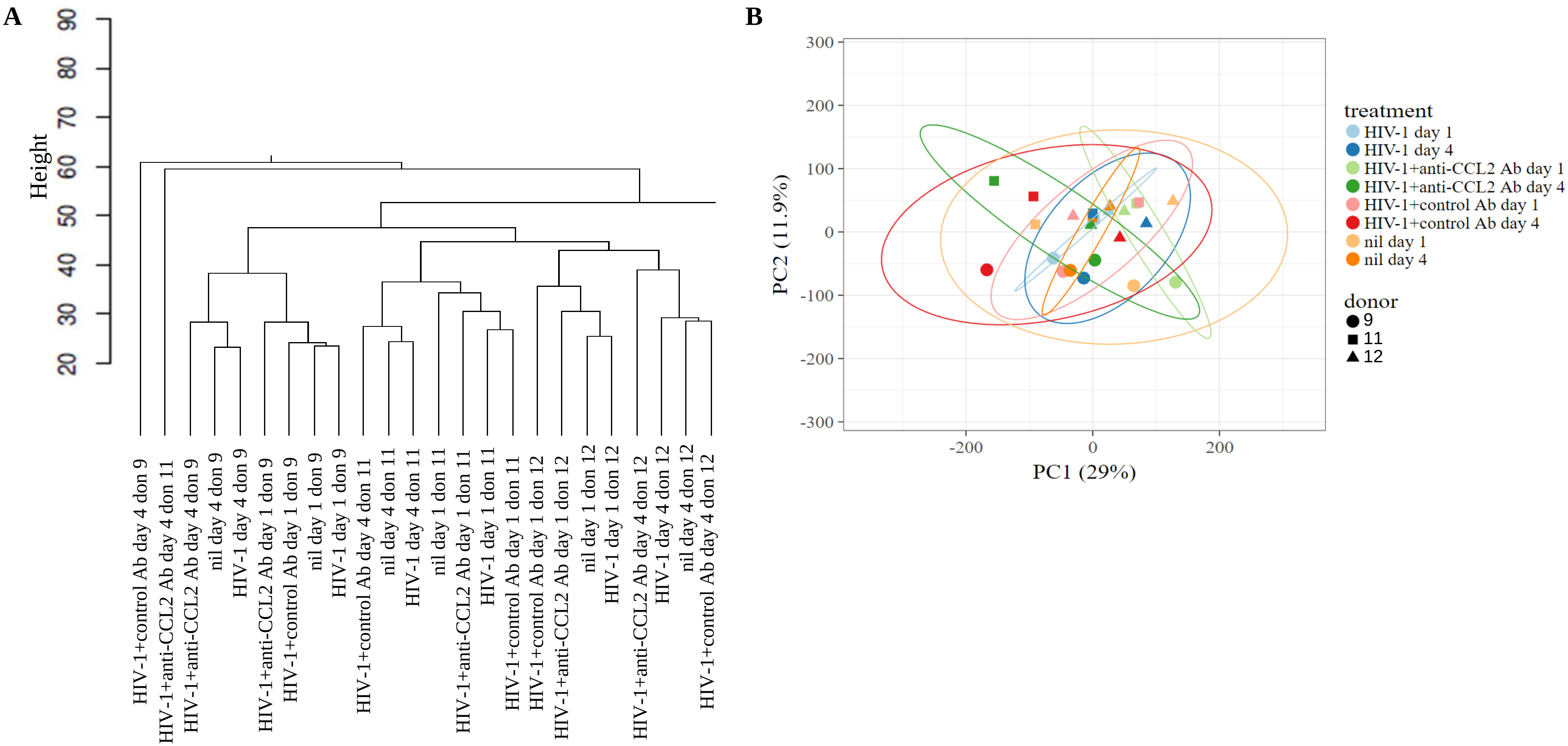

Supplementary Figure 7. Clustering of RNA-seq dataset 3. MDMs obtained from 3 different donors were treated or not with anti-CCL2 or control Ab (2.5 µg/ml) for 4 h and then infected with HIV-1. Total RNA was extracted at day 1 and 4 p.i., subjected to poly(A) selection followed by reverse transcription, generation of cDNA libraries, and sequencing. (A) Hierarchical clustering of RNA-seq data. (B) Principal component analysis of RNA-seq data. Unit variance scaling was applied to rows; SVD with imputation was used to calculate principal components; n = 24 data points. Prediction ellipses are such that with probability 0.95, a new observation from the same group will fall inside the ellipse.
